# Ascorbyl Radicals as Reducing Agents in Copper-Catalyzed Redox Reactions

**DOI:** 10.1021/acs.inorgchem.5c00934

**Published:** 2025-09-12

**Authors:** Caio Bezerra de Castro, Gabrielle Conciani, Everton M. da Silva, Joao Honorato, Walber Gonçalves Guimarães Júnior, André Farias de Moura, Javier Ellena, Arlene G. Corrêa, Otaciro Rangel Nascimento, Caterina G.C. Marques Netto

**Affiliations:** † 67828 Universidade Federal de São Carlos (UFSCar), Rod. Washington Luiz, km 235, São Carlos, São Paulo CEP 13565-905, Brazil; ‡ Instituto de Química, Departamento de Química Fundamental, Universidade de São Paulo(USP), Av.Prof.Dr.Lineu Prestes, 748, São Paulo, São Paulo CEP 05513-970, Brazil; § Instituto de Física de São Carlos 117186 Universidade de São Paulo (USP) Av. Joao Dagnone, 1100, São Carlos, São Paulo CEP 13563-120, Brazil

## Abstract

Radical-based redox reactions are greatly influenced by their surrounding environment, with the solvent playing a pivotal yet sometimes underestimated role. In this study, we examined how copper catalysts and the choice of solvent impact the reductive power of ascorbyl radicals. Our study used the reduction of 4-nitrophenyl azide as a model and further extended it to other azides and aldehydes. The results reveal a striking difference in radical stability and reductive efficiency, with higher conversions in methanolic solutions compared to acetonitrile. This difference was attributed to the formation and persistence of ascorbyl radicals in methanolic solutions as in acetonitrile; the copper complexes were fully reduced to their copper­(I) forms, and the ascorbyl radicals were barely detectable via EPR spectroscopy. Conversely, in methanol, DMPO-trapped ascorbyl radicals persisted for extended periods, indicating that these radicals were the primary reducing agents. Theoretical calculations supported this hypothesis, indicating that these findings suggest that optimizing solvent and copper catalyst selection is crucial for enhancing the reductive power of ascorbyl radicals, with implications for other metal-mediated reductions.

## Graphical abstract

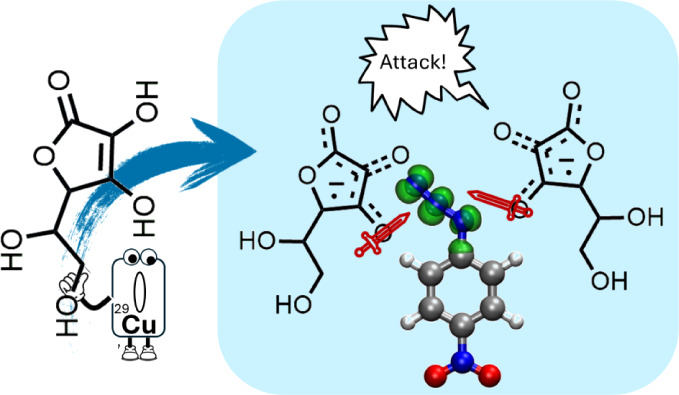

## Introduction

Radical-based chemistry has long been known to be an essential foundation in organic synthesis,^1^ where the effective use of radicals in redox reactions is strongly influenced by the reducing power and concentration of the active species, such as the one-electron-reduced molecule (R•^–^).^2^ Given that these reactions are typically carried out in homogeneous media, predominantly in solutions,^3^ the role of the solvent, as a major component of the reaction solution, cannot be overlooked.^3–8^ Solvents have been shown to significantly affect rate accelerations,^9,10^ product selectivity,^11–14^ and reaction mechanisms.^15–20^ In redox reactions, both entropic and enthalpic factors contribute to the solvation free energy of the solute, and the hydrogen bond donor ability of the solvent can notably influence the reaction’s entropy.^21^ Additionally, solvent effects on radical reactions become particularly important after radical formation.^22^ For example, polar solvents can accelerate reactions if dipolarity increases along the reaction coordinate.^23^ Moreover, the complexation of phenoxyl or alkoxyl radicals by metals or hydrogen bonds greatly enhances their persistence by reducing their involvement in disproportionation reactions.^24–26^

An illustrative example of the solvent influence in reactions is the copper-mediated reduction of azides to amines.^27^ The earliest reports of this process highlighted the critical role of water in determining the reaction’s outcome.^28^ According to the proposed mechanism, the reaction begins with the coordination of the azide to copper, followed by nitrogen extrusion and the formation of a transient nitrene species.^28–30^ Nitrenes, key intermediates in nitrogen atom transfer reactions, play a crucial role in this process.^29,31–35^ Lancaster, Betley, and colleagues^32^ isolated a copper-nitrene complex, providing evidence of radical formation during azide reduction. This resulted in a mixture of configurations: Cu­(I)-triplet nitrene, Cu­(II)–^2^NR charge transfer, and Cu­(III)–imido. The Cu­(I)-triplet nitrene configuration is preferred due to the significant N 2p character in the singly occupied molecular orbitals (SOMOs), creating a highly reactive nitrogen center due to its electron deficiency.^36^ The high reactivity of copper-nitrenes necessitates anaerobic and anhydrous conditions for isolation, as the presence of water leads to amine formation instead.^37^ Since nitrenes are unstable in aqueous environments, their involvement in the reaction mechanism under such conditions is supported primarily by DFT calculations.^37^ However, the choice of solvent in copper-mediated azide reductions, such as ethanol/water,^38^ DMSO/water,^27,39^ toluene,^40^ THF,^41^ and THF/water,^42^ suggests different operative mechanisms influenced by the solvent. For example, DMSO forms a DMSO-adduct in the reaction, while *t*-butanol generates *t*-butoxy radicals.^28^ Toluene interacts with copper intermediates via π-interactions, transferring a sp^3^ hydrogen to the amido ligand^37^ (Figure 1). These examples underscore the critical role of radical species in copper-catalyzed azide reductions and the importance of solvent selection in these processes.

**Figure 1:**
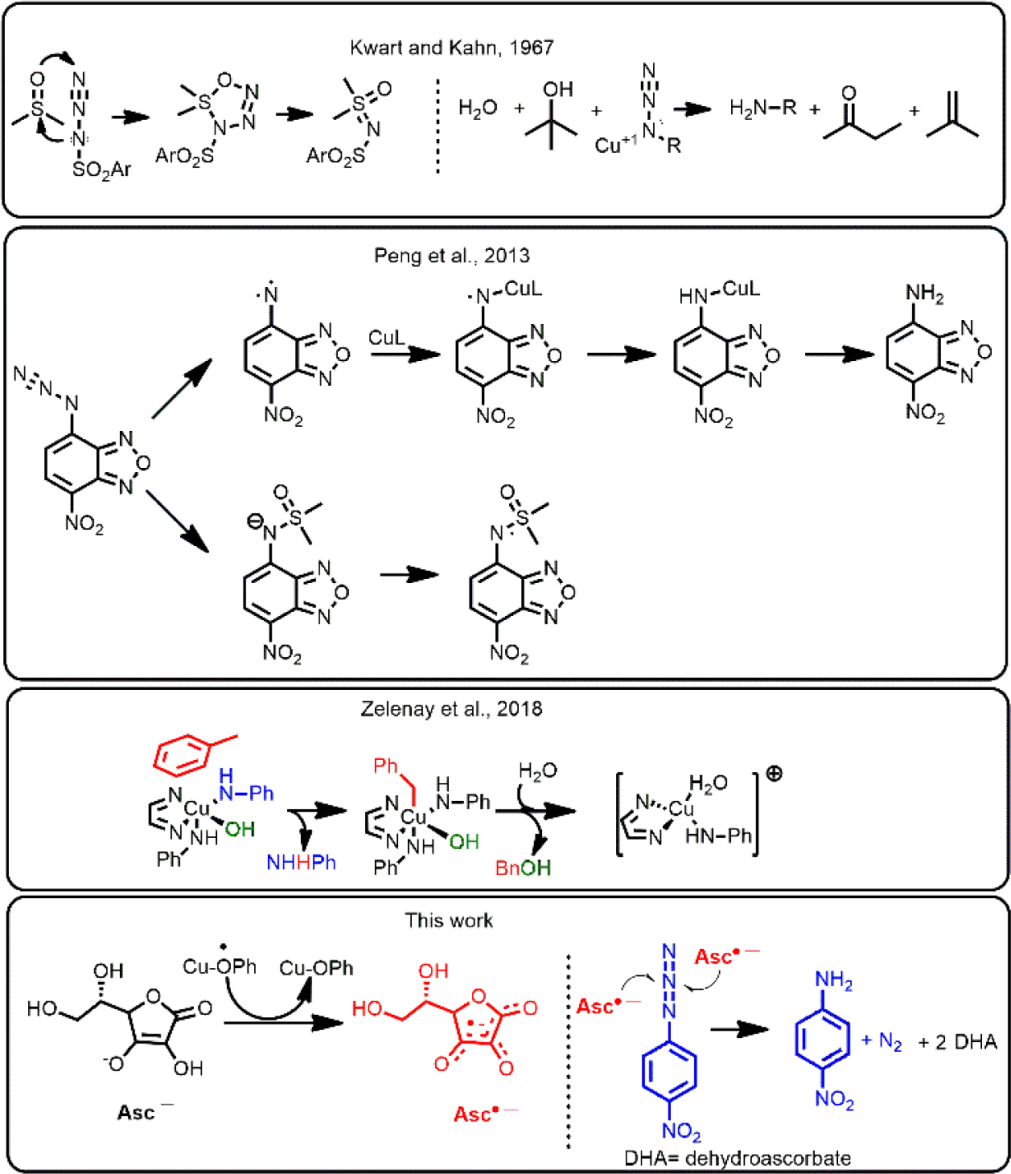
Examples of the influence of the solvent in the mechanism proposed for copper-mediated azide-to-amine reductions.

Interestingly, radicals have been shown to promote azide reductions, even in the absence of copper, highlighting their important role in these reactions. For instance, stannanes and silanes have been reported to operate through radical-based mechanisms.^43–45^ Another example is ascorbate, a reducing agent that proceeds via a radical intermediate and has been shown to reduce azides in electrochemical reactions.^46^ Although ascorbate is often considered a two-electron reducing agent, this process occurs through two consecutive one-electron oxidations, involving the formation of the ascorbyl radical (Asc•−) followed by dehydroascorbate.^47^ The involvement of ascorbyl radicals in side reactions during cycloadditions for the formation of amines from azides has received less attention.^38,39,42^ Given the relative instability of the ascorbyl radical, which often undergoes disproportionation into dehydroascorbate and ascorbate,^48^ it is challenging to directly observe this species and its role in redox reactions. Nevertheless, under anhydrous conditions, the ascorbyl radical can persist for several hours when generated from ascorbate by phenoxyl radicals.^49^

Motivated by the potential role of ascorbate in copper-catalyzed reductions and the intriguing influence of solvents on these reactions, we synthesized two novel series of copper complexes and evaluated their redox catalytic activity in two distinct solvent systems. Remarkably, these complexes stabilize phenoxyl radicals through coordination with the copper center, facilitating the generation of stable ascorbyl radicals. In these systems, the persistence of ascorbyl radicals proved instrumental in enabling their application as reducing agents for azide and aldehyde reduction, revealing an unexpected reaction mechanism.

## Results

### Complex Synthesis and Characterization

The complexes in this study were designed with distinct differences in their first and second coordination spheres. Ligands L1H and L2H include different donor atoms to coordinate to the metal center, such as a nitrogen atom derived from a pyrrolidine group, a nitrogen atom from an imine, and an oxygen atom from a phenolate. In contrast, ligands L3H and L4H were synthesized with an additional nitrogen donor atom from a pendent pyridine group (Scheme 1). All ligands were synthesized following established protocols previously described by our group^20,50^ and characterizations such as NMR (^1^H, ^13^C, COSY, HMBC, and HSQC) and HRMS confirmed their structures. The detailed synthesis and characterization procedures for these ligands are provided in Figures S1–S15. The reaction of the ligands with copper perchlorate resulted in the formation of complexes **1a**–**b** and **2a**–**b**. Complexes **1c** and **2c** were synthesized from copper nitrate on a small scale (10 mg), as shown in Scheme 1. These nitrate complexes were used exclusively for single-crystal X-ray diffraction studies to obtain structural evidence of the coordination, as the corresponding perchlorate analogues did not yield suitable crystals for analysis, with the exception of **2a**. Thus, all catalytic procedures were performed using perchlorate complexes (**1a**–**b** and **2a**–**b**).

**Scheme 1:**
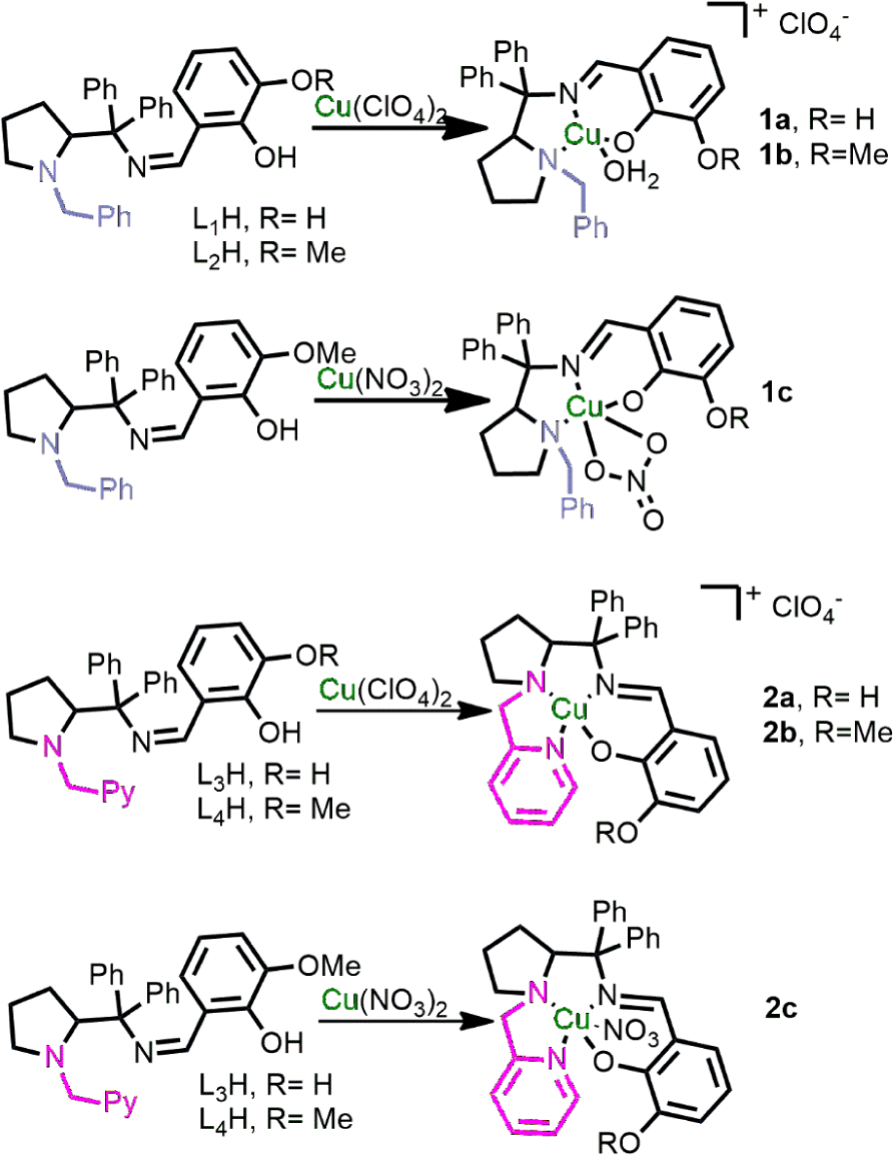
Synthesis of Complexes **1a**–**c** and **2a**–**c** from the Schiff-Base Ligands

All complexes were fully characterized by high-resolution mass spectrometry (HRMS), satisfactory microanalysis, EPR, FTIR, cyclic voltammetry, and electronic spectroscopy (Figures S16–S24). Single-crystal X-ray diffraction of complexes **1c** and **2c** (Figure 2A,B) revealed different coordination modes of nitrate depending on the ligand (L2H or L4H). For instance, a chelate coordination mode was observed for **1c**, whereas in **2c**, nitrate was observed as a monodentate ligand. The structure of **2a** (Figure 2C) provides evidence of the coordination of one solvent molecule in a similar position to the nitrate coordination in **2c**, revealing that the nitrate coordination mode could be used to assume the number of labile positions present in the complexes of this study. Therefore, complexes from series **1** have two labile positions, whereas complexes from series **2** have one.

**Figure 2:**
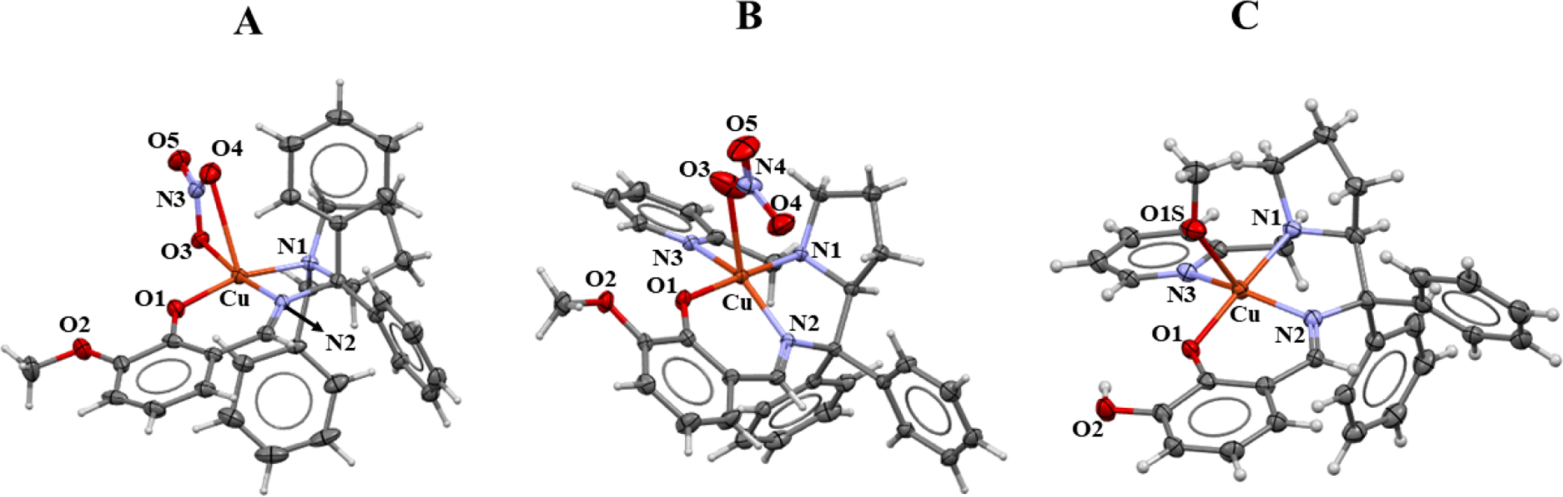
ORTEP representation of **1c** (A), **2c** (B), and **2a** (C) with ellipsoids shown at 30% probability.

For a better comparison of the catalysis based on the complexes’ structural differences, we chose complexes **1b** and **2b** to serve as models of both series. An initial assessment of the redox potentials of the complexes was performed by cyclic voltammetry (CV). To ensure the reliability of the redox potentials in our analysis, we performed a CV of ferrocene under similar experimental conditions to those of the copper complexes, observing a ferrocene/ferrocenium couple at 400 mV (vs. Ag/AgCl (3.5 mol L^–1^)), in agreement with the literature (Figure S22).^51^ Complex **1b** was evaluated in both methanol and acetonitrile, and in both solvents, no reversible processes were observed in the range of 465–795 mV. In acetonitrile, **1b** presented two reduction processes centered at −300 mV and −710 mV (vs. Ag/AgCl (3.5 mol L^–1^), Figure 3A). The first one was ascribed to the reduction of Cu­(II) to Cu­(I), whereas the second one was ascribed to a ligand moiety. In methanol, only one redox process was observed at −570 mV (vs. Ag/AgCl (3.5 mol L^–1^), Figure 3A), ascribed to the reduction of the metallic center. The non-reversibility of the processes might be associated with an EC (electrochemical-chemical) mechanism,^52^ such that once the copper center is reduced, the electron is transferred to a ligand moiety, and the copper is reoxidized in this process. To verify this possibility, we performed a spectroelectrochemical measurement of complex **1b** in acetonitrile and observed a blueshift of the LMCT band centered at 390 to 376 nm (Figure S25). These results, together with the CV data, suggest the presence of a ligand-based moiety capable of accepting electrons at the observed potentials. This behavior is consistent with the reduction or consumption of a phenoxyl radical within the ligand framework. Complex **2b**, on the other hand, presents a quasi-reversible redox process at *E*1/2 of −500 mV (vs. Ag/AgCl (3.5 mol L^–1^), Figure 3B) and −560 mV (vs. Ag/AgCl (3.5 mol L^–1^), Figure 3B) in methanol and acetonitrile, respectively, ascribed to the Cu­(II)/Cu­(I) redox pair.

**Figure 3:**
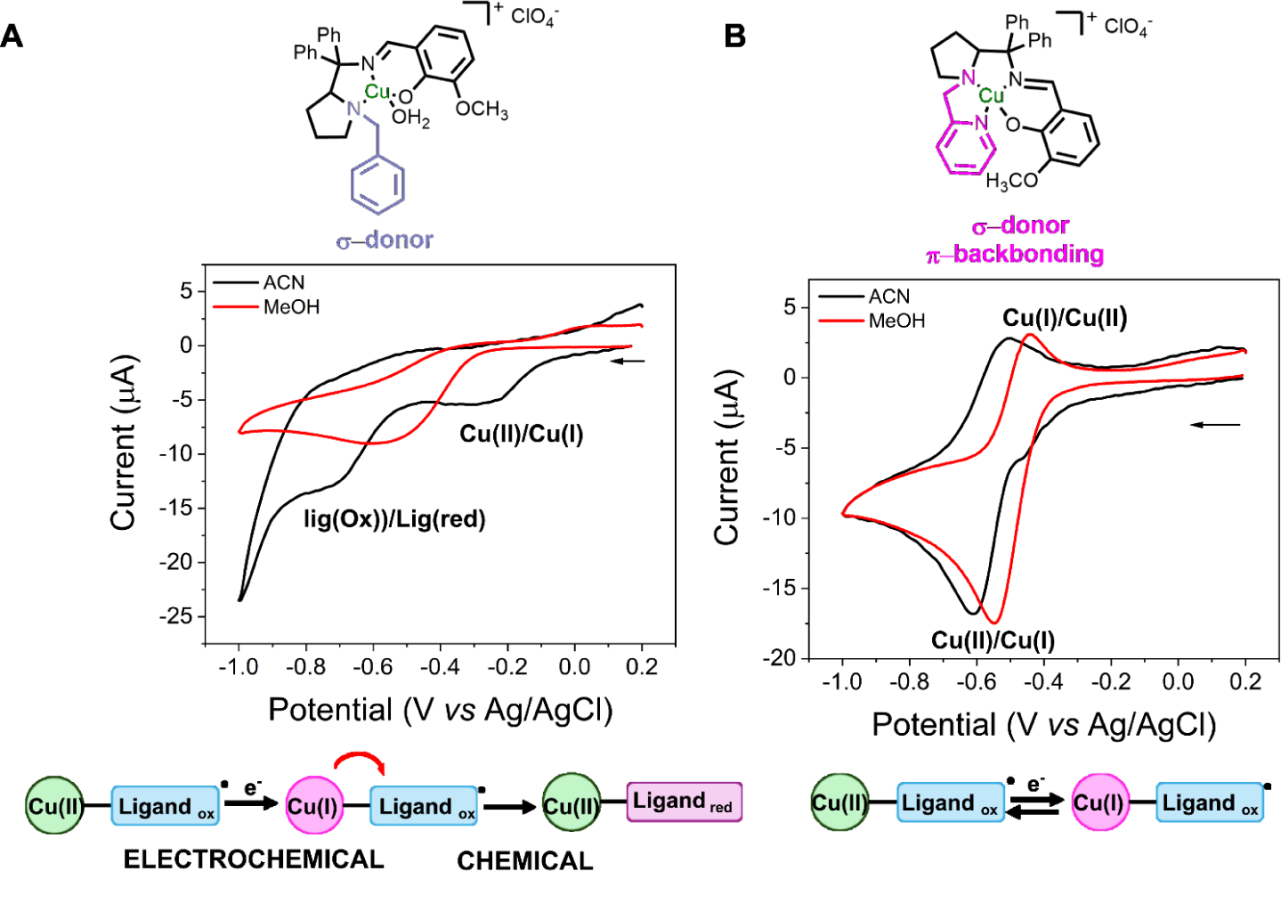
Cyclic voltammetry in methanol and acetonitrile of complexes **1b** (A) and **2b** (B), and schemes of the proposed mechanisms associated with the observed cyclic voltammograms. Assays were recorded at 100 mVs^–1^ using 0.1 mol L^–1^ of tetrabutylammonium perchlorate (TBAP) as an electrolyte, vitreous carbon as the working electrode, Pt as the counter electrode, and Ag/AgCl (3.5 mol L^–1^) as reference.

The difference between the CV spectra of **1b** and **2b** could be attributed to the presence of a pyridine-ligand moiety in **2b**, which, due to its π-back-bonding properties, stabilizes the Cu­(I) state, whereas the benzyl group in **1b** is non-coordinating and donates electron density to the pyrrolidine ring, enabling the chemical electron transfer from Cu­(I) to an oxidized ligand moiety. Another possible explanation is the lability of water molecules, which can be replaced by solvent molecules, resulting in solvent-coordinated species. In this scenario, the methanolic complex might differ from the acetonitrile-solvated counterpart. However, complex **2b** also has a labile position, and its redox potential shift is low when compared to **1b**. Moreover, no major spectroscopic changes were observed in the UV–vis spectra of these complexes in these solvents, indicating that the geometries of the complexes should remain the same. Thus, the difference between the cyclic voltammograms of complexes **1b** and **2b** in different solvents is most probably associated with the different mechanisms of redox reactions rather than different solvated species.

Ligand-centered redox events may alter the initial composition of the solution. For example, the observed reduction or consumption of a phenoxyl radical within the ligand framework suggests the presence of a phenoxyl species in solution. This has also been reported by other groups, either through the direct oxidation of a Cu­(II)-phenolate complex to a Cu­(II)-phenoxyl species^53^ or via tautomerization to a Cu­(I)-phenoxyl species followed by oxidation of the Cu­(I) center.^54^ While phenol oxidation is a two-electron process that occurs at high positive potentials, the one-electron oxidation of phenolate to phenoxyl radical occurs at more negative potentials.^55,56^ Therefore, considering that the driving force for valence tautomerization between Cu­(II)–phenolate and Cu­(I)–phenoxyl species depends on the Δ*E* (V) value between the phenolate/phenoxyl and Cu­(I)/Cu­(II) couples, we focused our attention on identifying a redox process that could support such a transformation. The redox potentials for phenolate/phenoxyl radical couples typically fall within the range of 0.17 to 0.56 V vs. Fc^0/+^;^57^ thus, we performed CV measurements over a broader potential window, from −2.0 V to +0.8 V vs. Ag/AgCl, as shown in Figure S26A. Under these conditions, a quasi-reversible redox couple is observed at approximately −1.7 V in all complexes. To assess whether this redox process is ligand-centered, we also recorded the CV of the free ligands from **series 2** and confirmed that this process is indeed associated with the ligand (Figure S26B). Therefore, we ascribe this pair as the phenolate-phenoxyl radical, which in turn makes it thermodynamically feasible to obtain a Cu­(I)-phenoxyl species. This assignment is further supported by DMPO-trapping EPR spectroscopy of the free ligand L3H, following its reaction with potassium ferricyanide in alkaline acetonitrile.^58^ Under these conditions, a mixture of hydroxyl- and carbon-centered radicals was detected (Figure S26C). In acetonitrile, a [Fe^III/II^(CN)_6_]^3–/4–^ redox couple is reported at −1.4 V vs. Fc^0/+^.^59^ This redox potential is thermodynamically compatible with the proposed phenolate/phenoxyl couple, supporting feasible electron transfer from phenolate to [Fe^III/II^(CN)_6_]^3–^ and thereby confirming our initial assignment. Thus, once Cu­(I)-phenoxyl can be formed spontaneously, the oxidation of Cu­(I) to Cu­(II) by air is straightforward. However, while it is thermodynamically possible, the transformation of the Cu­(II) species into the Cu­(I) species faces a kinetic barrier associated with geometry conformation changes.^57^ For instance, a coordination environment of the Cu center close to a four-coordinate tetrahedral geometry stabilizes the Cu­(I)–phenoxyl radical complex, but our complexes bear a distorted square-pyramidal structure (Figure 2), which supports a Cu­(II) oxidation state. A distorted square-pyramidal complex able to perform this interconversion into a tetrahedral geometry was reported to bear the phenolate/phenoxyl couple at −0.41 V vs. Fc^0/+^.^54^ Therefore, only a minor portion of the solution was observed in the Cu­(I) state. As can be observed in Figure 3, a minor redox process at −460 mV is detected in the CV experiments of complex **2b**. Considering that a tautomerization followed by oxidation would lead to two different Cu­(II) species in solution (Cu­(II)-phenolate and Cu­(II)-phenoxyl), we ascribe this minor process to the Cu­(II)-phenoxyl/Cu­(I)-phenoxyl redox couple.

What is interesting to note from these wider redox window experiments is that when complex **2a** is fully reduced, an additional oxidation process at −270 mV can be observed (Figure S26A). This process is also observed if 1 equiv of ascorbate is added to the solution (Figure S26D), indicating that this could be due to the generation of Cu­(II)-phenoxyl to Cu­(I)-phenoxyl as a result of chemical reduction. The presence of hydrogen bonds in ligand L3H might stabilize the phenoxyl moieties by hydrogen bonds,^26^ enabling the persistence of Cu­(I)-phenoxyl in solution for longer periods and allowing for the visualization of the Cu­(I)-phenoxyl/Cu­(II)-phenoxyl redox pair.

### Reaction with Ascorbate

Considering the redox potentials of complexes **1b** and **2b**, along with the redox potential of ascorbate (Asc•/Asc^–^ potential at −300 mV vs. Ag/AgCl (3.5 mol L^–1^)),^60^ it can be assumed that the use of ascorbate as a reducing agent to reduce the copper centers will only be efficient for complexes of series **1** in acetonitrile (**1a** and **1b**).

As expected from the redox potential, complex **2b** was not reduced by sodium ascorbate in neither solvent, as evidenced by the EPR measurement (Figure S27). However, the addition of sodium ascorbate to **1b** in an acetonitrile solution enabled the reduction of **1b** to **1b’** (Figure 4A) as observed by the disappearance of the d–d band centered at 598 nm and the red shift of the LMCT band from 388 to 420 nm (Figure 4B). EPR analysis of **1b’** in an acetonitrile solution also confirmed the reduction of Cu­(II) to Cu­(I) upon ascorbate addition (Figure 4D). For instance, in Figure 4D, it is evident that the four-line Hallmark pattern resulting from the hyperfine coupling of the electron spin of Cu­(II) with the *I* = 3/2 nuclear spin of the Cu^63/65^ isotopes (100% natural abundance) is not present in **1b’**. On the other hand, the behavior of complex **1b** in methanolic solutions upon the addition of ascorbate was different. As can be seen in Figure 4C, the addition of sodium ascorbate to a methanolic solution of **1b** resulted in a broadening of the d–d band and a slight blue shift of the LMCT band from 388 to 382 nm. The fact that the d–d band was still present after ascorbate addition reveals that Cu­(II) was not reduced to Cu­(I), which was confirmed by EPR spectroscopy (Figure 4E), with the copper center before and after ascorbate addition exhibiting similar g_II_ and g_⊥_ parameters (see Table S1). Spin quantification of the EPR spectra reveals that the experimental spins relative to Cu­(II) were about 81% of the expected value, which remained the same after the addition of ascorbate in a methanolic solution of **1b**. Therefore, the metallic center of complex **1b** is not reduced by ascorbate in methanol.

**Figure 4:**
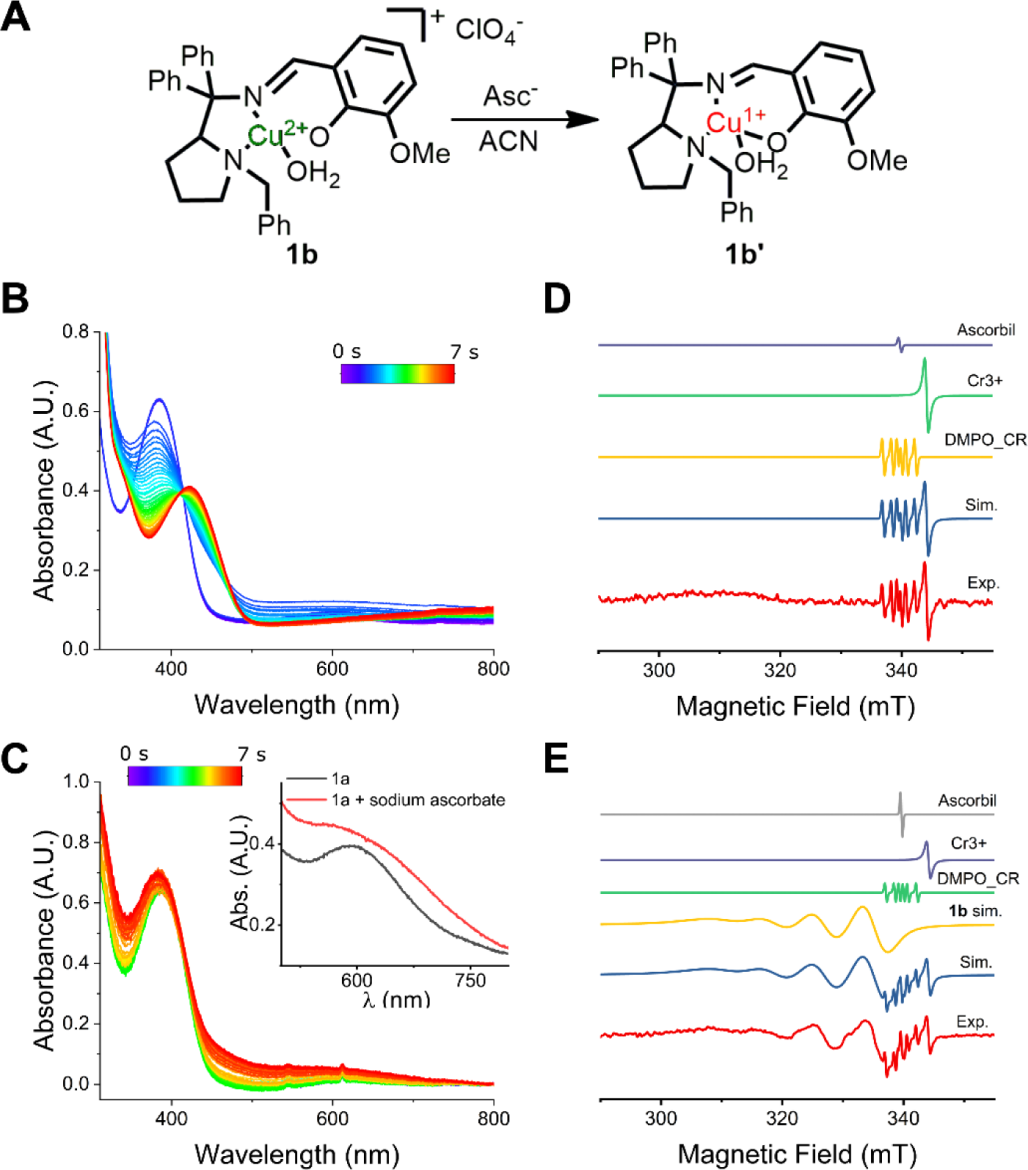
Effect of sodium ascorbate addition on complex **1b** in different solvents. (A) Proposed reaction in acetonitrile, (B) UV–vis monitoring of the reaction between **1b** and ascorbate in acetonitrile (C) and in methanol. Inset of the d–d band region before and after sodium ascorbate addition. (D) EPR spectra of the reaction mixture in acetonitrile (red) with the simulated Cr­(III), DMPO-O-ascorbyl, and DMPO-C-ascorbyl, evidencing the reduction of copper in acetonitrile. (E) EPR spectra of the reaction mixture in methanol (red) with the simulated Cr­(III), DMPO-O-ascorbyl, DMPO-C-ascorbyl, and Cu­(II), evidencing that Cu­(II) remains intact.

### Azide-to-Amine Reduction

Considering the redox states of the complexes in both solvents, we decided to evaluate their ability to act as catalysts in reducing reactions. Our initial substrate was chosen based on the ability of Cu­(I) to reduce azides, where we expected to observe higher conversions of azide reduction into amine in acetonitrile in the presence of complexes from series **1**. In these reactions, sodium ascorbate was used as a sacrificial electron donor, aiming to reduce the copper center and generate the proposed copper-nitrene intermediate.^61^ Protic solvents (methanol, water, and 2,2,2-trifluoroethanol (TFE)) were used to verify if they could influence the reaction.^62,63^ Therefore, we explored the reduction of 4-nitrophenylazide in different solvent mixtures: methanol/water, methanol/TFE, acetonitrile/water, and acetonitrile/TFE in the presence of sodium ascorbate (Table 1). Control reactions without the copper complexes did not form the amine product (Table 1, entries 1, 2, 15, and 16). Experiments using copper perchlorate and a free ligand resulted in 15% and 27% conversions (Table 1, entries 3 and 4), respectively, indicating that a small conversion was obtained even without the complex. Moreover, it also points to a possible strong role of the ligand in the catalysis.

**Table 1:**
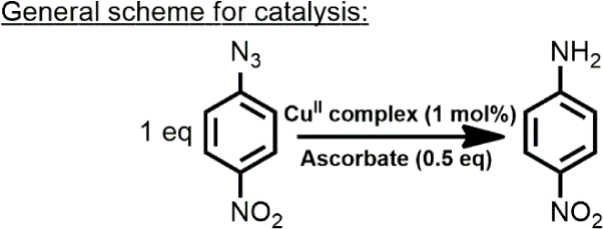
Reduction of *p*-Nitrophenylazide under Different Conditions^a^

Entry^a^	Catalyst	Solvent mixture	Conversion (%)^b^
1	-	CH_3_CN/TFE	0
2	-	CH_3_OH/TFE	0
3	**L2H**	CH_3_OH/TFE	27
4	Cu(ClO_4_)_2_	CH_3_OH/TFE	15
5	**1a**	CH_3_CN/TFE	2
6	**1a**	CH_3_OH/TFE	60
7	**1a** ^c^	CH_3_OH/TFE	90
8	**1b**	CH_3_CN/TFE	6
9	**1b**	CH_3_OH/TFE	41
10	**2a**	CH_3_CN/TFE	4
11	**2a**	CH_3_OH/TFE	66
12	**2b**	CH_3_CN/TFE	3
13	**2b**	CH_3_OH/TFE	68
14	**2a** ^c^	CH_3_OH/TFE	85/91^d^
15	-	CH_3_CN/H_2_O	0
16	-	CH_3_OH/H_2_O	0
17	**1a**	CH_3_CN/H_2_O	5
18	**1a**	CH_3_OH/H_2_O	57
19	**1b**	CH_3_CN/H_2_O	<1
20	**1b**	CH_3_OH/H_2_O	54
21	**2a**	CH_3_CN/H_2_O	1
22	**2a**	CH_3_OH/H_2_O	22
23	**2b**	CH_3_CN/H_2_O	2
24	**2b**	CH_3_OH/H_2_O	18
25	**2b(red)** ^e^	CH_3_OH/H_2_O	0

a All reactions were performed at 80 °C with 60 min of reaction.

b determined by ^1^H NMR.

c Sodium ascorbate was used in equimolar concentration to the substrate.

d The yield, determined after product isolation and purification.

e **2b** (red) is complex **2b** with a Cu­(I) center. The solvent mixuture used in these reactions involved a 96/4 (v/v) proportion of solvents.

The reactions using our copper complexes as catalysts revealed that the solvent mixture had a stronger impact on the reaction conversion than the difference between complex series (Table 1). For instance, both series presented a higher production of *p*-nitroaniline in methanol/TFE and methanol/H_2_O mixtures than in acetonitrile/TFE (or H_2_O) mixtures. For comparison, entries 5, 6, 10, and 11 can be taken as examples, where **1a** (entries 5 and 6) and **2a** (entries 10 and 11) complexes were employed in acetonitrile/TFE and methanol/TFE mixtures, resulting in low conversions of *p*-nitrophenylazide in acetonitrile mixtures (2–4%) and higher conversions in methanolic mixtures (60–66%). Interestingly, TFE had no impact on the reaction conversion for complexes from series **1** (Table 1, entries 5–9 and 17–20). However, an increase in conversion from ≈20% to ≈67% was observed for series **2** upon the change from water to TFE (Table 1, entries 10–14 and 21–24). These differences between series **1** and **2** reveal that the proton transfer might have a different mechanism in complexes **2a** and **2b** than in complexes **1a** and **1b**.

To establish why the solvent mixture was important to improve the reaction conversion, we monitored the reaction by ^1^H NMR (Figure S28), observing that in an acetonitrile/water mixture, the reaction stopped the amine production after half an hour, whereas in methanol/water, the reaction proceeded. This could indicate that either the complex was poisoned in acetonitrile or it was degraded during the reaction. HRMS analysis of the reaction in these solvent mixtures using complex **1a** as a catalyst revealed that degradation was the main issue. For example, in methanol and in the presence of sodium ascorbate, **1a** was mostly present as the intact complex (calc. for C_31_H_29_CuN_2_O_2_ *m*/*z* 524.15204, found 524.15250). However, signals referring to the free ligand and the interaction between **1a** and sodium ascorbate were also present (Figure S29A). The addition of azide to this reaction mixture increased the formation of the free ligand, indicative of labilization. Concomitant to labilization was the emergence of a signal at *m*/*z* 986.38319, attributed to the interaction between **1a** and the free ligand (Figure S29B). Even with the formation of these other species, the signal of the initial complex was still strong in the HRMS spectrum in methanolic solutions. On the other hand, in acetonitrile, a much stronger labilization of the ligand was observed even in the absence of azide (Figure S29C,D). Beyond that, a peak at *m*/*z* corresponding to **1a’+H** ^**+**^ (calc. for C_31_H_30_CuN_2_O_2_ *m*/*z* 525.16032, found 525.15964) was observed, evidencing the reduction of the copper center in this solvent mixture. Therefore, we can assume that the reduction of the copper center from Cu­(II) to Cu­(I) labilizes the ligand, destroying the complex and decreasing the reaction conversion. This is in agreement with a control reaction performed in the absence of ascorbate but in the presence of the reduced species of **2a** (obtained from coordination with CuI), where we did not observe any amine production (Table 1, entry 25). With these results, we can infer that the best conversions for the reduction of *p*-nitrophenyl azide were obtained when complexes **1a**–**b** and **2a**–**b** were in the Cu­(II) state, since the complexes remained mostly intact. For example, HRMS of **2a** in the presence of ascorbate/azide did not evidence labilization (Figure S30), and as demonstrated by EPR and CV, complexes from series **2** were not reduced under these conditions.

These results indicate that the mechanism of the reduction of azide in our system differs from the expected copper­(I)-nitrene mechanism.^37^ Our suspicion was that ascorbate could be playing a major role in the reaction, as it can donate two electrons,^64^ and all reactions using 0.5 equiv of sodium ascorbate per azide exhibited lower conversions than 100% (complex **2b** exhibited 68% in a methanol/TFE mixture – Table 1, entry 13). Interestingly, the increase in sodium ascorbate concentration from 0.5 equiv to 1 equiv resulted in an increased conversion of azide into amine from 60% to 90% in the catalysis performed by **1a** in the methanol/TFE system (Table 1, entries 6 and 7). To assess the reliability of the conversions determined by ^1^H NMR of the crude reaction mixture, we compared these values with the isolated yield from a reaction conducted under optimized conditions (1 equiv of sodium ascorbate, 1 h reflux in a MeOH/TFE mixture). Under these conditions, the product was successfully isolated in 91% yield (Table 1, entry 24), closely matching the conversion estimated by ^1^H NMR analysis of the crude mixture (Table 1, entry 24), thereby validating the accuracy of the NMR-based quantification.

Based on these results, we hypothesized that ascorbate may donate only one electron during the reaction, leading to the formation of ascorbyl radicals. However, to rule out a copper-nitrene pathway, it was necessary to investigate the interaction between the copper complexes and the substrates, since the proposed mechanism for azide reduction by copper complexes involves azide coordination to the copper center.^28–30^

### Copper Interaction with Substrates or Products

We studied the complex interaction with the substrates by EPR spectroscopy. In these assays, we added azide and ascorbate to complexes **1b** and **2b** dissolved in methanol at 77 K (Figure 5). Complex **1b** remained unchanged upon azide addition, even after waiting 5 min of reaction, indicating that azide did not coordinate to the copper center (Figure 5A). After the addition of ascorbate, there were spectroscopic changes of the EPR parameters (Table S1) with more well-defined superhyperfine lines. This indicated a conformational change that could be due to azide coordination or other chemical changes in the complex. However, the addition of ascorbate to **1b** without azide produced a similar spectrum (Figure S31), revealing that azide does not coordinate during the reaction and that a chemical reaction likely occurs between **1b** and ascorbate, as already evidenced by the HRMS experiments discussed in the previous section (Figure S29). In the EPR experiments of **1b**, there was a decrease in the superhyperfine lines over time, suggesting that the complex returned to its initial state. In contrast, the EPR parameters of complex **2b** remained largely unchanged upon the addition of either azide or ascorbate, aside from a slight broadening of the g_*z*_. This suggests that if any interaction between **2b** and azide occurs, it is weak or transient in nature (Figures 5B and S27).

**Figure 5:**
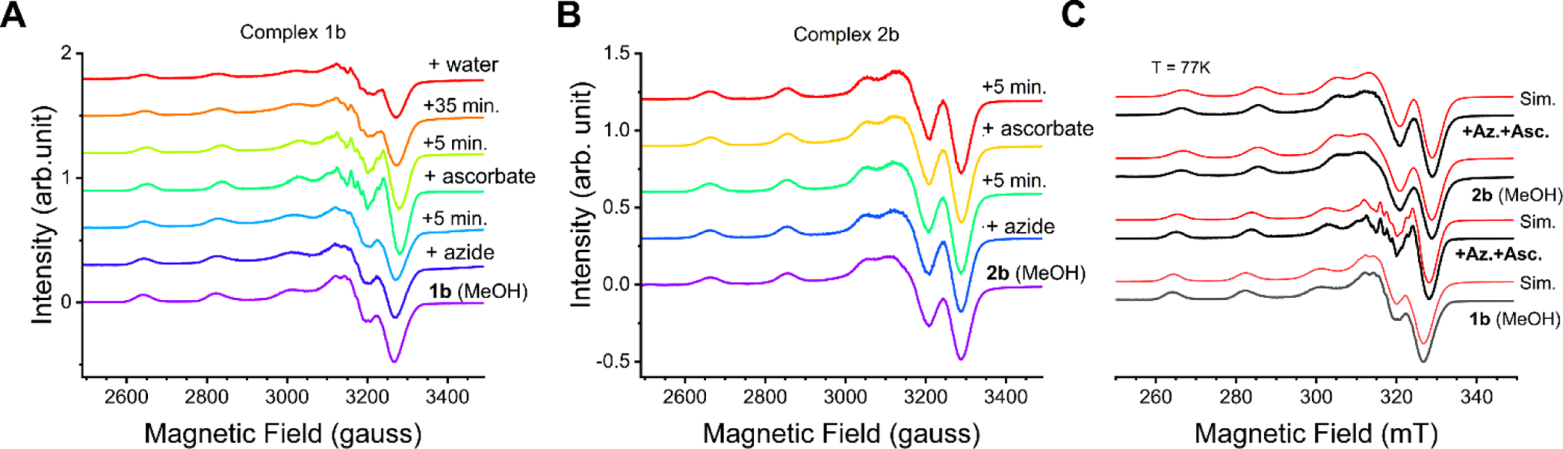
EPR spectra recorded at 77 K of the reaction between **1b** (A) and **2b** (B) with successive additions of azide and ascorbate, and (C) comparison between EPR spectra and simulation.

### Radicals as Catalysts

Intrigued by the lack of interaction between azide and the complexes (Figure 5) and noticing that ascorbic acid could donate only one electron,^65^ we suspected that ascorbate could be cycling between ascorbic acid and the monodehydroascorbyl radical.^66^ Therefore, we decided to evaluate the presence of radical species during the reaction using EPR spectroscopy and DMPO (5,5-dimethyl pyrrolidine oxide) as a radical trapping agent.^67,68^ In these reactions, DMPO was used in a large excess (100 mmol L^–1^) to offset the poor reaction kinetics.^69^ Both complexes (**1b** and **2b**) in the absence of ascorbate and azide revealed the presence of an oxygen-centered radical species in both solvents (methanol and acetonitrile)^70^ (Figure 6A). Spin quantification revealed that these radicals are present in ≈4% of the total spins. Complex **2b** in acetonitrile revealed slightly different EPR spectra, exhibiting 4 lines instead of 6. However, over time, **2b** converted the 4-line spectra into the same 6-line spectra of an oxygen-centered radical trapped by DMPO (aN = 13.87, aH = 7.92) (Figure 6B). Considering this and the structure of the complexes, the most probable source of an oxygen-centered radical would be the phenol moiety, and the 4-line to 6-line change over time was attributed to the detachment of the phenol moiety from the copper center due to the reaction with DMPO, as shown in Figure 6C. The addition of DMPO to these reactions did not change the spectrum of the copper region (Figure S32). Importantly, HRMS of the reaction between **1b** and DMPO (Figure S33C) revealed the expected mass for a DMPO-trapped **1b** radical. Spin quantification at 0 min showed 81% Cu­(II) and 2.5% O-centered radical, while at 14 min, the values shifted to 83% Cu­(II) and 4.2% O-centered radical. This suggests that an initially EPR-silent Cu­(II)-•OPh species may become EPR-active due to an equilibrium shift in the DMPO-trapping reaction (Figure 6C). Consequently, the phenoxyl radical may account for up to 20% of the solution, considering that approximately 80% is attributed to non-silent Cu­(II).^71^

**Figure 6:**
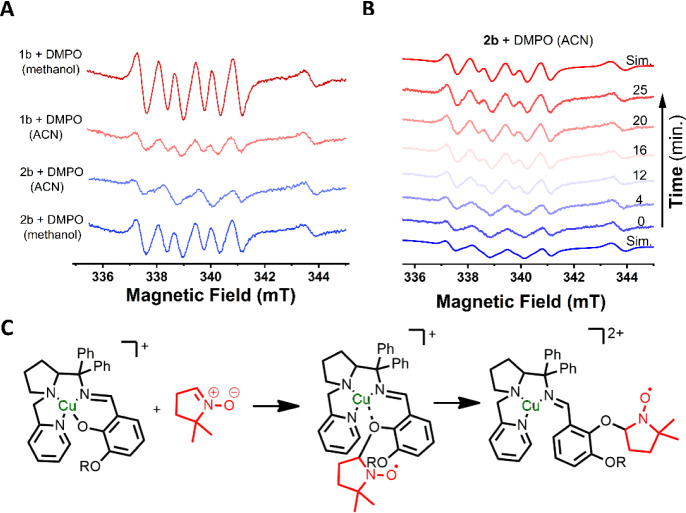
Detection of the phenoxyl radical by spin-trapping. A) EPR spectra of the reaction between DMPO and complexes **1b** and **2b** in acetonitrile and methanol. B) Time-resolved evolution of the EPR of the reaction between DMPO and **2b** in acetonitrile, evidencing the change from 4-line to 6-line spectra over time. C) DMPO spin-trapping proposed mechanism. The baseline of the EPR spectra is the high-field broad Cu­(II) EPR lines, as evidenced in Figure 4(E). The Cu­(II) center was intact after DMPO addition (see Figure S29).

To verify whether the oxygen-centered radical could be phenoxyl, we added ferrocyanide to the complex in the presence of DMPO and analyzed the EPR and UV–vis spectra. Interestingly, we observed the consumption of the oxygen-centered radical upon the addition of ferrocyanide (Figure S33), indicating that a one-electron oxidation of ferrocyanide to ferricyanide accompanied the reduction of the oxygen-centered radical, as would be expected for the phenoxyl radical reduction.^72,73^ Monitoring the reaction of ferrocyanide addition to **1b** by UV–vis spectroscopy resulted in only slight changes (Figure S33), such as a blueshift of the LMCT band centered at 402 to 397 nm. This change can be associated with the consumption of a phenoxyl radical,^74^ since bathochromic shifts and increased intensity of bands are associated with phenolate upon one-electron oxidation.^75^ These changes were already observed in the spectroelectrochemical experiments (Figure S26) and in the reaction between **1b** and sodium ascorbate (Figure 4D). Another piece of evidence for the presence of phenoxyl radicals was obtained by XPS. Comparison of the XPS binding energies (Figure S34) between the free ligand (L3H) and the complex shows a slight increase in the O 1s and C 1s signals associated with C–OH (aromatic) (from 532.2 eV in L3H to 532.4 eV in **2a**), C–C/C–H (from 285.4 eV in L3H to 285.9 eV in **2a**), and C–Py (from 284.5 eV in L3H to 284.8 eV in **2a**) upon complexation. These shifts suggest the formation of a stronger phenoxylic moiety when L3H coordinates to Cu­(II), consistent with the generation of a phenoxyl radical-Cu­(II) species (PhO•–Cu­(II)), due to a stronger C=O character and a lower resonance in the aromatic ring. In contrast, the N 1s binding energies associated with N–pyrrole and N–pyridine decrease upon complexation, shifting from 399.8 to 399.6 eV and from 399.0 to 398.5 eV, respectively. This trend may indicate that following PhO•–Cu­(II) formation, electron density is drawn toward the phenolic group, resulting in a reduced bond energy associated with the nitrogen atoms. These results (XPS, HRMS of **1b**-DMPO, EPR, and reactivity with ferricyanide) indicate the formation of a PhO•–Cu­(II) species in the complex. The presence of non-innocent ligands in these complexes appears to play a key role in maintaining the redox state of the metal center, acting as an electron reservoir in the formation of the phenoxyl radicals.^57,76^

Copper-phenoxyl complexes have been extensively studied in the literature since the discovery of the copper-tyrosyl radical in galactose oxidase enzymes.^77–79^ Their formation can be associated with tautomerism,^54^ loss of coordinated solvent,^80^ or the use of excess copper during synthesis.^79,81^ Considering these possibilities, we propose that tautomerization followed by oxidation is operative in our system, as illustrated in Figure 7A. This mechanism for phenoxyl radical formation has been previously reported, wherein molecular oxygen is reduced by a tautomeric Cu­(I)-phenoxyl radical species, leading to the generation of hydrogen peroxide.^82^ Based on this precedent, we hypothesized that bubbling O_2_ through a methanolic solution of the complex would produce H_2_O_2_ if a similar mechanism happens. Indeed, upon O_2_ bubbling, the absorption band centered at 629 nm decreased, while a new band at 562 nm concurrently increased (Figure 7B), as monitored by UV–vis spectroscopy. These spectral changes were accompanied by the colorimetric detection of hydrogen peroxide using a ferrous ammonium sulfate–thiocyanate assay^83^ (Figure 7C). Blank experiments in a similar setup, using a copper complex that does not bear the Cu­(II)-phenolate/Cu­(I)-phenoxyl tautomerism^50^ form significantly lower levels of H_2_O_2_, as can be seen from the lower intensity of the band centered at 480 nm (Figure S35). Thus, the observed H_2_O_2_ formation is due to the reaction between the Cu­(I)-phenoxyl tautomer and the O_2_, in agreement with the Cu­(I)-phenoxyl tautomer formation. Together, these observations support that tautomerization followed by aerial oxidation is the operative pathway for the formation of the Cu­(II)-phenoxyl radical in our system.

**Figure 7:**
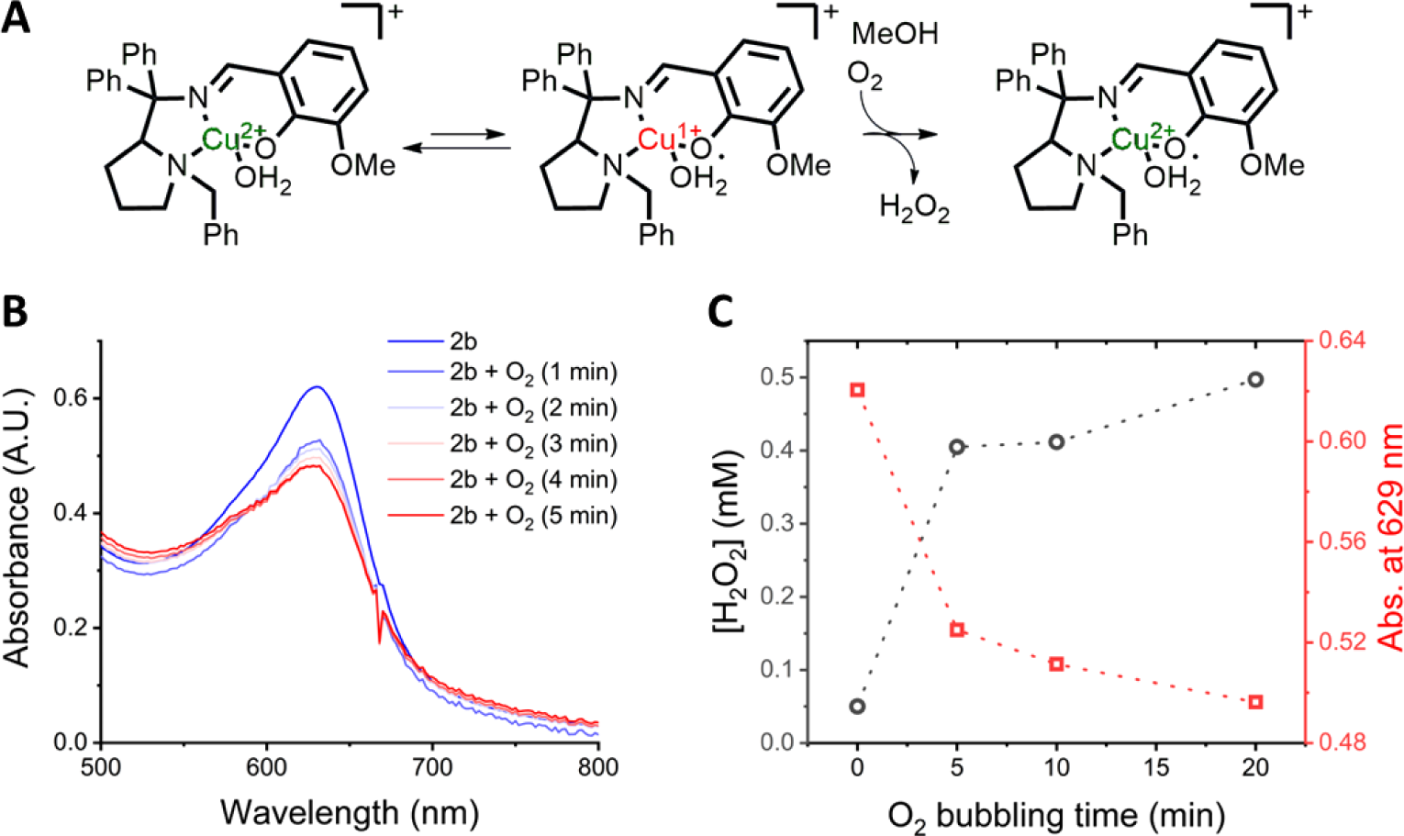
**Copper­(II)-phenoxyl radical formation**. A) Proposed mechanism involving tautomerism followed by aerial oxidation, B) UV–vis spectral changes in the region between 500 and 800 nm upon O_2_ bubbling, and C) relationship between the concentration of peroxide in solution, O_2_ bubbling time, and the absorbance at 629 nm.

Metal-radical species are considered noninnocent,^82,84^ where depending on the relative energies of the redox-active orbitals, these metal complexes can exist in two limiting descriptions: either as a metal–ligand radical (M^*n*^+(L̇)) or as a high-valent metal complex (M­(^*n*^+1)+(L^–^)).^82^ Here, we observed that the copper center remained in its Cu­(II) state, and we were able to spin-trap the oxygen-centered radical using DMPO, observing via EPR and HRMS the copper-phenoxyl DMPO-trapped radical. Thus, we ascribe the copper-phenoxyl as a metal–ligand radical (M^*n*^+(L̇)).

Considering that radicals were present at the start of the reaction, we monitored the azide reduction reaction via EPR, by radical trapping with DMPO. Time-resolution EPR of DMPO-trapped radicals was evaluated in both solvents (acetonitrile and methanol) and is shown in Figure 8. In these experiments, it was possible to observe that in methanol, only ascorbyl radicals could be observed over time (carbon- and oxygen-centered).^85^ These radicals account for ≈1% of the total spins, remaining stable over a long period of time (30 min), representing a persistent fraction of the solution. However, in acetonitrile, the ascorbyl radical was only observed at the beginning of the reaction (Figure 8B, 0 min), being quickly dominated by other carbon-centered radicals, with lower stability, as they started to decay over time. A similar trend was also observed for complex **2b** (Figure S36).

**Figure 8:**
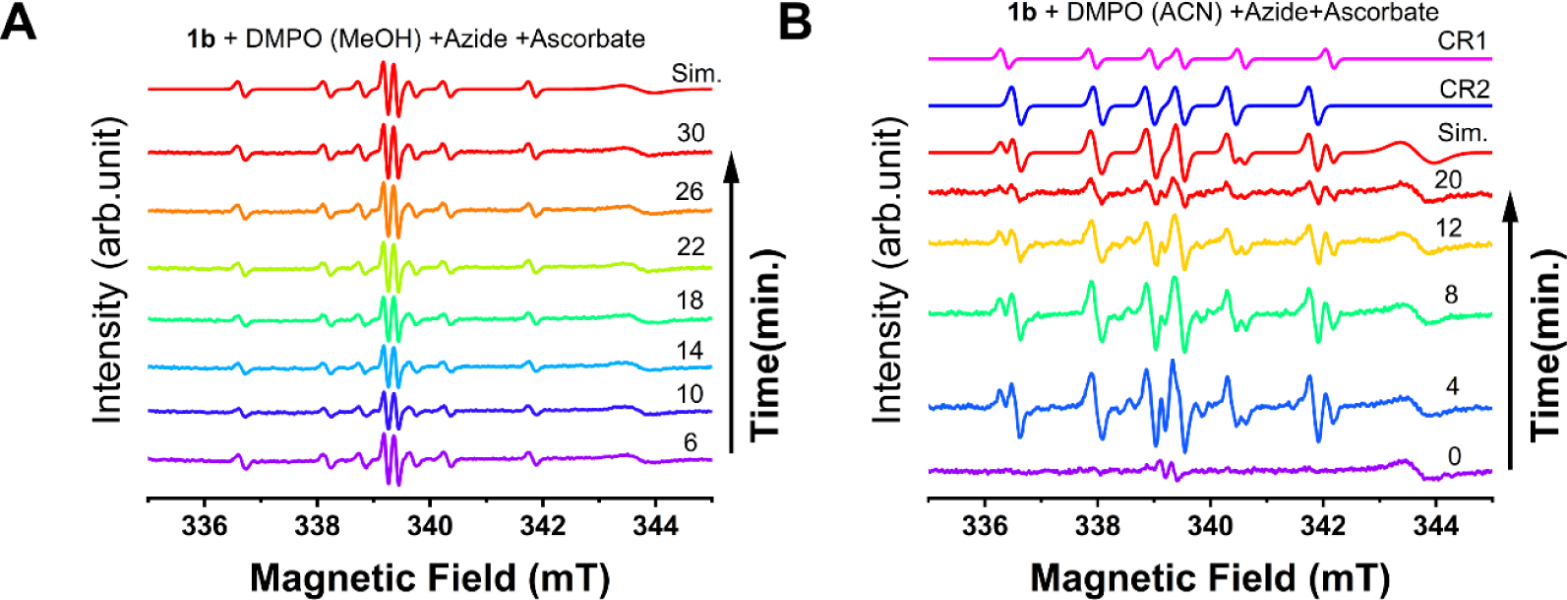
Time-resolved EPR spectra recorded at room temperature of the reaction between **1b**, azide, and ascorbate in the presence of DMPO as a radical trapping agent. (A) Reaction performed in methanol and (B) reaction performed in acetonitrile. The spectrum indicated as Sim. is the simulated spectrum where the O-ascorbyl and C-ascorbyl radicals were added.

It is important to note that no nitrogen-centered radical could be trapped under our experimental conditions, indicating that no copper-nitrene was formed during the reaction. This is in agreement with the EPR analysis, where no azide-copper complex interaction could be observed (Figure 5).

Given that the complexes in solution exhibited phenoxyl radicals and displayed varying behaviors in the presence of ascorbate, depending on the solvent, we suggested two distinct reaction pathways. For instance, the copper center of **1b** was reduced in acetonitrile, revealing that two electrons were involved in the reaction: one to reduce the copper center and the other to reduce the phenoxyl radical. Since in acetonitrile the ascorbyl radical is not stable (Figure 8), we propose that the phenoxyl radical was reduced by the ascorbyl radical. This resulted in a labile Cu­(I) complex that degraded over time, explaining why the complex was not active in acetonitrile. Supporting this hypothesis is the HRMS of the reaction in acetonitrile, where a free ligand was observed (Figure S29). In addition to the HRMS experiment, an ^1^H NMR experiment of **1b** in acetonitrile/water in the presence of sodium ascorbate revealed the presence of O-vanillin (Figure S37). O-vanillin could only be formed by hydrolysis of the ligand. Thus, it was evident that the acetonitrile medium was favoring degradation of the complex. In contrast, in a methanolic medium, ascorbate was not able to reduce the copper center but could reduce the phenoxyl radical, acting as a single-electron donor^64^ generating ascorbyl radicals (Figure 8). Additionally, HRMS analysis of the reaction in methanol revealed a high abundance of the intact complex (Figure S29). Since no nitrogen-centered radical was detected, we propose that the reduction of the azide to the corresponding amine likely occurs via a concerted electron and hydrogen transfer from two ascorbyl molecules, as illustrated in Scheme 2. Thus, the proposed reaction steps for the catalysis involve the generation of the catalytically active species Cu­(II)-phenoxyl radical via tautomerism followed by oxidation (Scheme 2A, step 1), reaction between the Cu­(II)-phenoxyl radical and ascorbate to generate Cu­(II)-phenolate and the ascorbyl radical (Scheme 2A, step 2), and the reaction between two ascorbyl radicals and one azide (Scheme 2A, step 3), enabling the catalytic cycle depicted in Scheme 2B. This hypothesis might seem odd as the ascorbyl radical is not stable and cannot be isolated in the solid state. However, a higher persistence of alkoxyl radicals was observed when these radicals were coordinated to metals.^24–26^ In fact, we observed signals referring to the interaction between **1a** and sodium ascorbate (Figure S29A), indicating the coordination of ascorbate to the metal center, which might enable its use as a reductant.

**Scheme 2:**
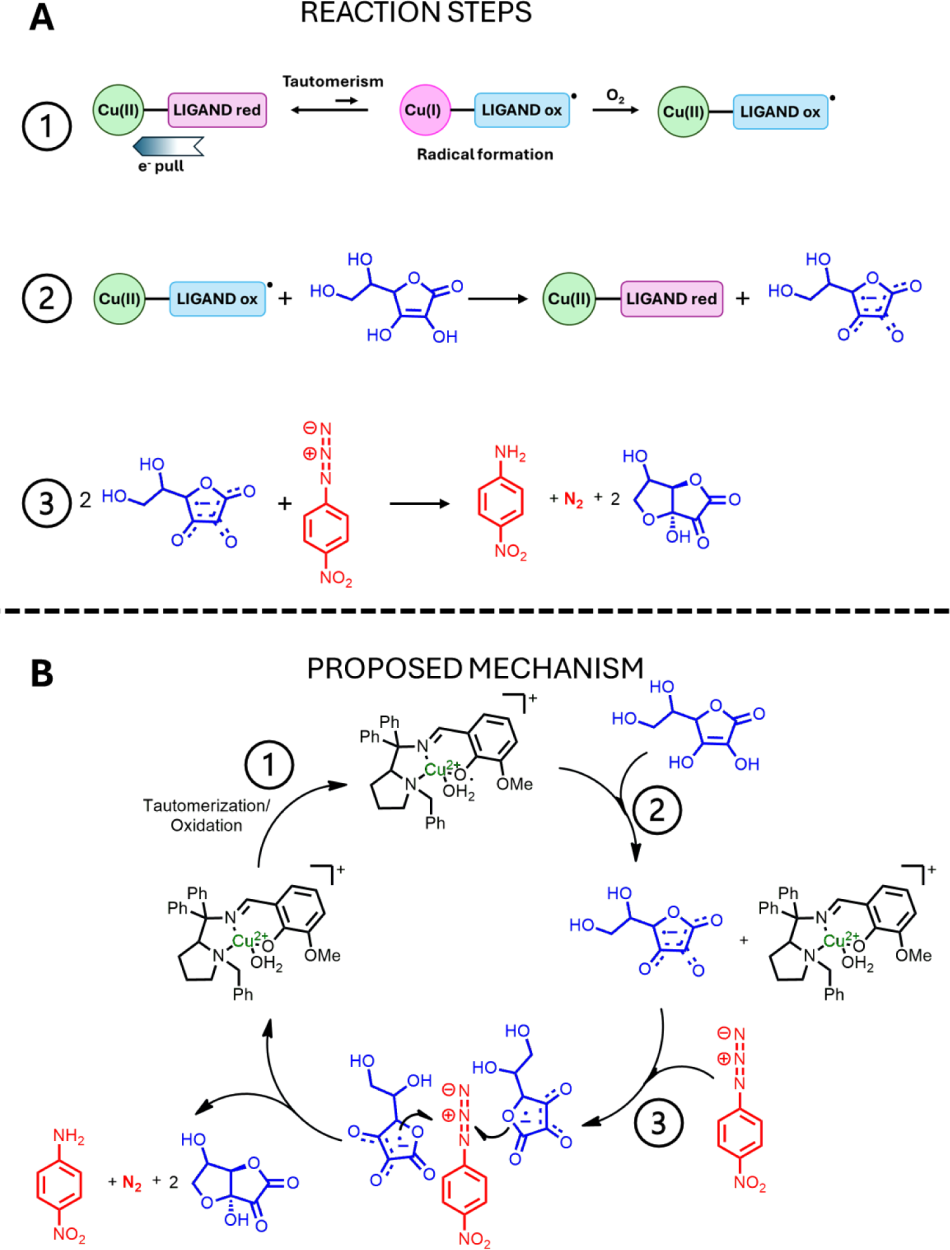
A) Proposed Reaction Steps of the Azide Reduction: 1) Generation of the Catalytic Active Species Cu­(II)-Phenoxyl Radical via Tautomerism Followed by Oxidation, 2) Reaction between Cu­(II)-Phenoxyl Radical and Ascorbate to Generate Cu­(II)-Phenolate and Ascorbyl Radical, and 3) Reaction between Ascorbyl Radicals and Azide; B) Proposed Catalytic Reaction Cycle of Azide Reduction by the Copper Complexes of This Work; the First Step Is the Generation of Ascorbyl Radicals by the Phenoxyl Radical Consumption with Ascorbate; the Second Step Is the Electron Transfer from the Radical to the Azide, Resulting in Dinitrogen Extrusion and Amine Synthesis; Regeneration of the Phenoxyl Radical Is Proposed to Pass through a Tautomerization Followed by an Oxidation Step

For complexes from series 2, the mechanism should be slightly different, as the interaction between **2a** and ascorbate was not observed either by EPR or HRMS. Under these conditions, ascorbyl radicals would be expected to react with protons (H^+^) in the solution more rapidly than with azide. Curiously, complexes from series 2 exhibit higher reaction conversions in TFE/methanol mixtures in comparison to those in H_2_O/methanol. This enhanced reactivity is likely attributed to differences in the interaction between the complexes and the substrate, particularly in the proton transfer step between the solvent and the azide. A faster proton transfer between the solvent and azide would prevent the reprotonation of the ascorbyl radical, thereby avoiding its disproportionation. Considering the p*K*_a_ values of the solvents (methanol: 15.4; water: 14; TFE: 12.6), the higher acidity of TFE may facilitate this more efficient proton transfer. Solvent effects have also been shown to influence electron transfer in other ascorbate-mediated reactions.^86,87^ For example, in the electron transfer from ascorbate to ferricyanide, a proton-coupled electron transfer (PCET) mechanism is operative, and the addition of organic cosolvents to the aqueous medium enhances hydrogen tunneling contributions, particularly at higher cosolvent concentrations.^88^ Therefore, it is possible that proton transfer from ascorbate to azide could also involve tunneling mechanisms.^89,90^

Interestingly, azide reduction has been observed during azide–alkyne cycloadditions using copper and sodium ascorbate, particularly specific toward the ortho-azido substitution of the polycyclic system.^38,39,42^ In these reactions, the solvent and temperature were shown to influence the reaction pathway, with the addition of water enhancing the reduction.^39^ This was attributed to the formation of a nitrene intermediate that rapidly reacted with water to generate the corresponding amine. However, under our reaction conditions, we did not detect a nitrene. Importantly, the best reaction conditions in our study involved the copper center in a 2+ oxidation state. This oxidation state hinders azide coordination and the formation of a copper-nitrene intermediate, as Cu­(I)-nitrene is the preferred species formed. Therefore, the lack of nitrogen-centered radicals in the EPR is not surprising. Moreover, the most stable radical in the reaction was the DMPO-trapped ascorbyl radical, indicating its significant role in the mechanism.

### Mechanism Evaluation

Computational assessment was employed to examine the proposed mechanism. Three primary hypotheses regarding the reaction of ascorbate with copper complexes were investigated, as shown in Figure 8A. The first one involves a one-electron transfer from ascorbate to the phenoxyl moiety (R1). In the second hypothesis, two electrons are transferred: one to the copper center, reducing it to Cu­(I), and the other to the phenoxyl moiety (R2). The third hypothesis entails a one-electron transfer solely to the Cu center (R3). In both solvents, R1 was the most favorable energetically, exhibiting Δ*G* values between −15 and −30 kJ mol^–1^ depending on the starting complex (Figure 8B). R2 and R3 exhibited positive Δ*G* values, being nonspontaneous. We also evaluated the coordination of the azide to the copper center, observing positive Δ*G* values, indicating that azide does not coordinate to the copper center. This is in agreement with the EPR experiment, where no spectroscopic changes were observed upon azide addition to the complex (Figure 5). Considering the experimental evidence and the computational results, we postulate that the ascorbyl radical is formed from the reaction between ascorbate and the phenoxyl radical. The ascorbyl radical should interact with the azide to enable electron transfer. This reaction would need two electrons to be transferred, and for that, a stable conformational state between ascorbate/ascorbyl and azide/amine was calculated in both solvents by CREST (Figures 9C and S38). Interestingly, in both solvents, spontaneous reactions (Δ*G* values of −45 and −54 kJ mol^–1^, respectively, for acetonitrile and methanol) were obtained upon the interaction of ascorbyl and azide. Remarkably, all systems minimize energy by maximizing geometry with the greatest number of interactions possible. The ensemble between ascorbate and *p*-nitrophenyl azide has a preferential positioning of the ascorbate molecules over azide, due to the localization of the LUMO, as shown in Figure 9D. Overall, the proposed mechanism is energetically favored in methanol rather than in acetonitrile (Figure 9E and Table S2). For instance, the Δ*G*° using **1b** as a catalyst in methanol, in the reaction scheme shown in Figure 8F, was calculated to be −65.3 kJ mol^–1^, whereas in acetonitrile, this value rises to −12.1 kJmol^–1^. This indicates that the proposed mechanism is energetically feasible and agrees with our experimental findings.

**Figure 9:**
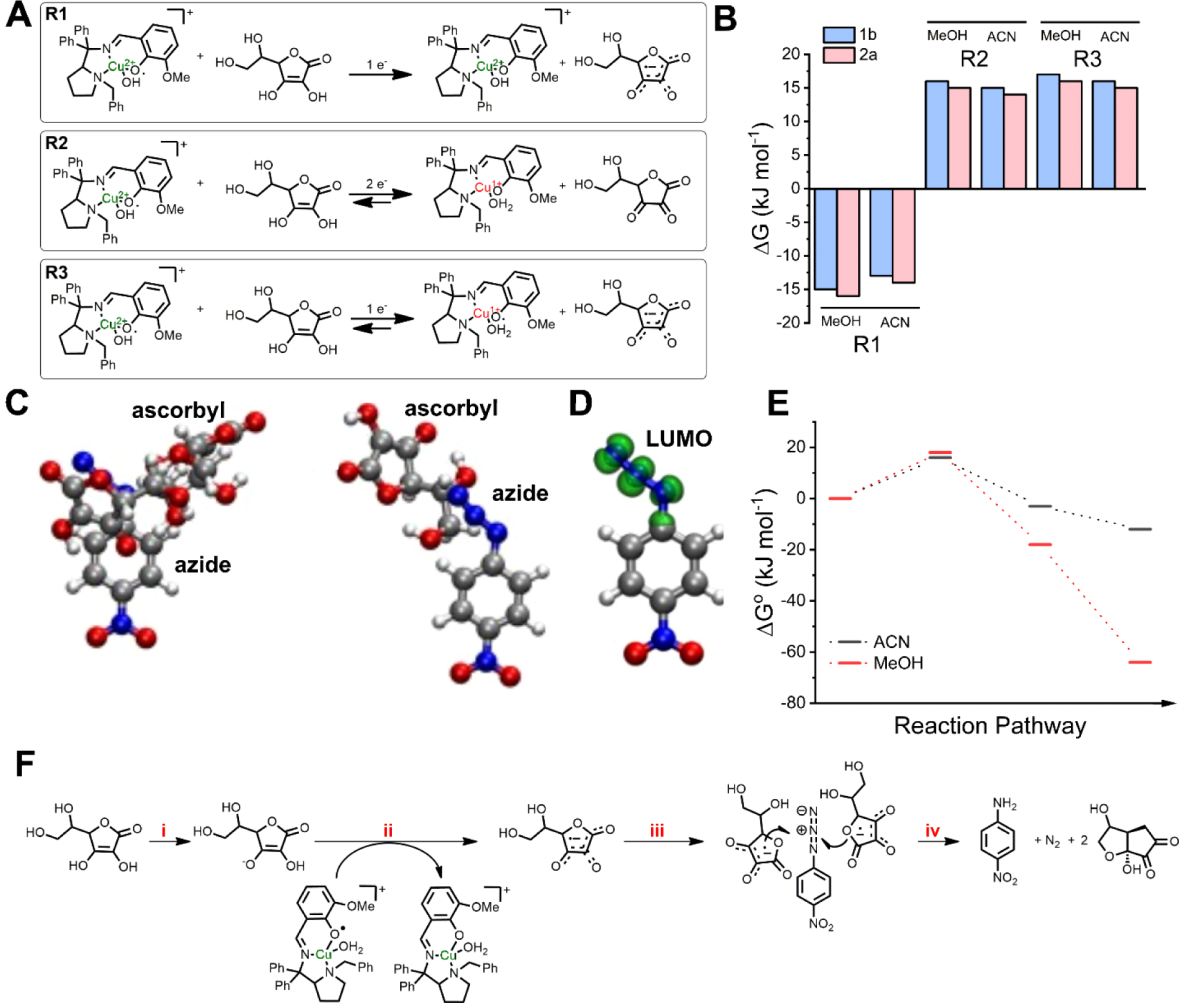
Reactions evaluated in theoretical calculations (A) and their relative free Gibbs energies (B). The ascorbyl radical interacts with the azide (C), where the LUMO of the *p*-nitrophenyl azide is localized (D). The relative Gibbs energies for each step were calculated in both solvents, and it is evident that the reaction in methanol is favored (E); the reaction steps depicted in Δ*G*° values are (i) ascorbate deprotonation, (ii) ascorbyl radical formation due to interaction with copper complexes, (iii) interaction of azide with two ascorbyl radicals, and (iv) interaction of azide with two ascorbyl radicals in the presence of copper complexes, forming amine and regenerating the phenoxyl-derived copper complex. (F) Depiction of the reaction steps considered in the calculations for obtaining the Δ*G*° values of shown in (E).

### Substrate Scope

Based on this mechanism, other azides and organic functionalities should also be reducible by the ascorbyl radical. To explore this, we tested four additional azides, four aldehydes, and three ketones under identical reaction conditions (methanol/water, 80 °C, 12 h, 2.2 equiv of ascorbate). An increase in reaction time and ascorbate load was performed to allow for a broader substrate scope, as these substrates are less reactive than *p*-nitrophenyl azide. We observed that all azides were reduced to their corresponding amines regardless of the aromatic substituent or whether the azide was aliphatic, as shown in Figure 10 (see raw NMR spectra in Figures S39–S46). This indicates that our methodology can be applied to different azides and differs from other existing azide-to-amine reaction approaches. For example, the classical methodology, known as the Staudinger reaction,^91^ typically involves the use of stoichiometric amounts of phosphites or phosphines under reflux for several hours. Therefore, an advantage of our methodology over the classical methodology is the removal of phosphites or phosphines from the reaction. In copper-catalyzed reduction of azides, these processes may involve the use of either stoichiometric amounts of Cu­(I) complexes, the reduction of a Cu­(II) complex to Cu­(I),^27,31,32,38,40^ or the use of hydrosilanes.^41^ Particularly against other copper-mediated approaches, our methodology has the ability to operate with catalytic amounts of copper complexes under nonstrict anaerobic conditions and in the presence of water.

**Figure 10:**
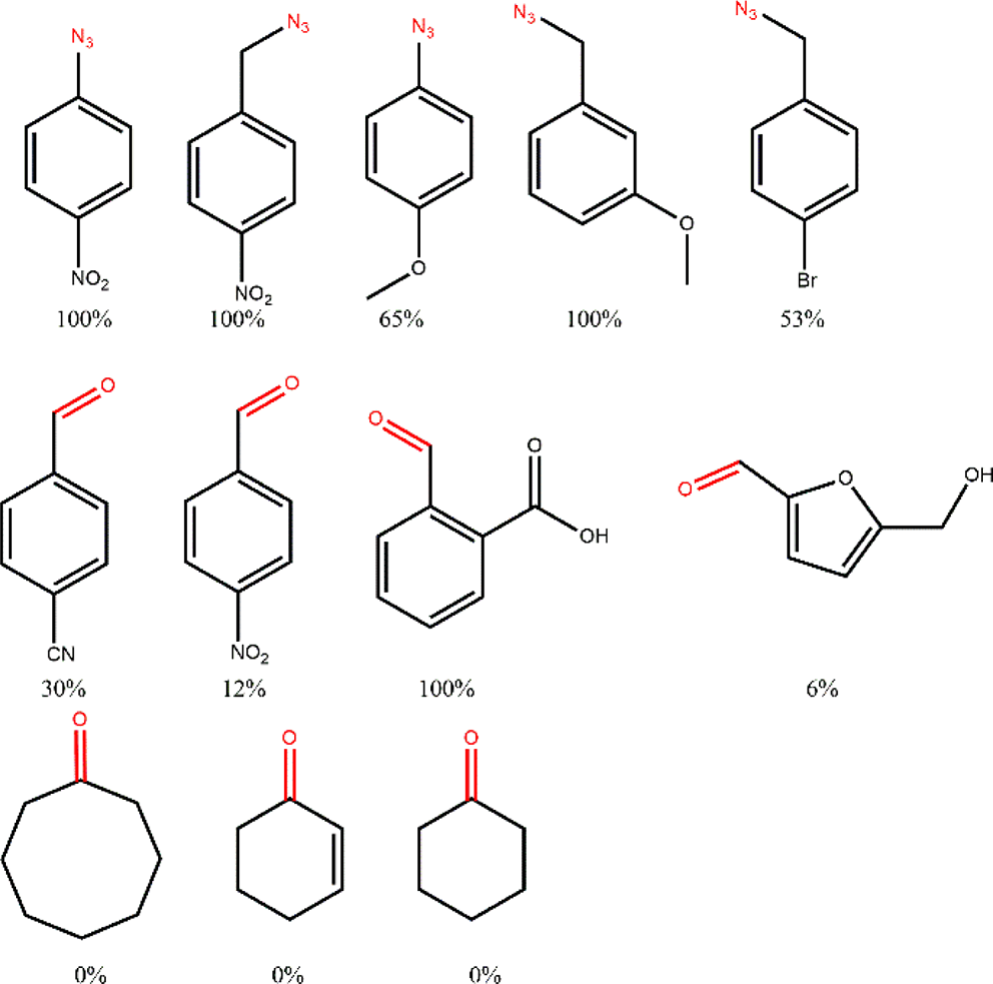
Compounds tested as substrates for the reaction and their conversions into their corresponding amine and alcohol. The conditions used were: 4 mL of methanol, 0.18 mmol of substrate, 2.2 equiv of ascorbate dissolved in 1.2 mL of water, 1 mol % of 2a, 80 °C, 12 h. All conversions were calculated based on ^1^H NMR.

In addition to azides, some aldehydes were also reduced to their corresponding alcohols using this methodology (Figures S47–S50), with conversions ranging from low (e.g., hydroxymethylfurfural, HMF, 6%) to high (e.g., 2-carboxybenzaldehyde, 100%). In contrast, ketones were not reduced under these conditions. These results are not surprising, as the carbon in ketones is less electrophilic than the carbon of aldehydes. Typically, reducing agents for ketones have a higher potential for reduction. For instance, sodium borohydride (NaBH_4_) has a redox potential of −1.24 V versus the standard hydrogen electrode (SHE), whereas ascorbate/ascorbate has a redox potential of −0.32 V vs. SHE.

These findings suggest that this method can be applied to a variety of substrates, and we believe that this is the first observation of the stabilization of an ascorbyl radical playing a role as a reducing agent.

## Discussion

The insights gained from our study on the role of solvents and radicals in azide reduction have broader implications across various chemical reactions. Ascorbic acid is commonly used as a sacrificial electron donor in numerous reactions, such as CO_2_ reductions into methanol,^92^ H_2_ generation,^93^ and various organic transformations.^94^ The solvent effect on the active species in these reactions might be overlooked, affecting their efficiency and selectivity.

Moreover, azide is a substrate of the nitrogenase enzyme that is responsible for the reduction of dinitrogen into ammonia, a crucial step in the nitrogen cycle. However, the mechanism of nitrogenase remains enigmatic.^95,96^ Our findings highlight the role of ascorbyl radicals as reductants in catalysis, suggesting that other radical-based reactions might occur in chemical reductions. For example, recent research has shown that a citrate radical acts as the reducing agent in the synthesis of metallic nanoparticles via the Turkevich methodology. Similarly, the first oxidation step of citrate involves H-atom abstraction, yielding Cit­(−H)•, a potent reducing agent.^97^ Considering the similarities between homocitrate and citrate, and the crucial role of homocitrate protonation in the nitrogenase mechanism, it is plausible that a homocitrate radical may function as a reducing agent in nitrogenase, shedding light on its elusive mechanism.^98^

## Experimental Section

### Synthesis of Copper Complexes (**1a**–**2b**)

A round-bottom flask was charged with Cu­(ClO_4_)_2_·6H_2_O (0.23 mmol, 1.1 equiv) and 6.00 mL of methanol, and the contents were stirred at 40 °C for 10 min until complete dissolution of the salt. Then, **L1H–L4H** (0.21 mmol, 1 equiv) was added to the reaction. After 4 h at 40 °C under stirring, the reaction was cooled to room temperature and filtered. To the solution, distilled water was added to precipitate the compounds. The solid was isolated via centrifugation at 5000 rpm for 5 min. The solid was washed with water and dried under vacuum for 3 days.

Complex **1a**: brown solid, 63% yield. Conductivity (MeOH, μS cm^–1^): 60.8 ± 0.2. HRMS isotopic cluster found 538.1677 (CuL–H_2_O) (calcd 538.1676). FTIR in KBr (cm^–1^) 3412, 1655, 1620, 1319, 1279, 1234, 1120, 1109, 1001. Anl. Calc. for C_31_H_31_ClCuN_2_O_7_: C 57.94, H: 4.86, N: 4.36. Found: C: 58.14; H: 4.94; N: 4.45

Complex **1b**: dark-green solid, 73% yield. Conductivity (MeOH, μS cm^–1^): 79.2 ± 0.2. HRMS isotopic cluster found 524.1522 (CuL–H_2_O) (calcd 524.1520). FTIR in KBr (cm^–1^) 3442, 1655, 1620, 1319, 1279, 1246, 1122, 1109, 1005. Anl. Calc. for C_32_H_33_ClCuN_2_O_7_: C 58.53, H: 5.07, N: 4.27. Found: C: 58.65; H: 5.16; N: 4.62

Complex **2a**: light green solid, 70% yield. Conductivity (MeOH, μS cm^–1^): 88 ± 0.2. HRMS isotopic cluster found 539.1631 (CuL) (calcd 539.1639). FTIR in KBr (cm^–1^) 3464, 1618, 1319, 1265, 1230, 1146, 1032, 1001. Anl. Calc. for C_30_H_28_ClCuN_3_O_6_: C 57.60, H: 4.51, N: 6.72. Found: C: 57.95; H: 4.91; N: 6.82

Complex **2b**: dark green solid, 86% yield. Conductivity (MeOH, μS cm^–1^): 112.9 ± 0.2. HRMS isotopic cluster found 525.1476 (CuL) (calcd 525.1423). FTIR in KBr (cm^–1^) 3444, 1604, 1340, 1296, 1248, 1167, 1142, 1032, 1005. Anl. Calc. for C_31_H_30_ClCuN_3_O_6_: C: 58.21, H: 4.73, N: 6.57. Found: C: 58.18; H: 4.78; N: 6.73

### Synthesis of Azides

**HAZARDS:** Azides are hazardous compounds due to their toxicity, explosiveness, and chemical reactivity. Sodium azide is highly sensitive to shock, heat, and friction. Organic azides can also be thermally unstable, particularly those with a low molecular weight or multiple azide groups. Use caution when handling them in their dry form or when using them in larger quantities.

### Alkyl Azides

The synthesis of 1-(azidomethyl)-4-bromobenzene, 1-(azidomethyl)-4-nitrobenzene, and 1-(azidomethyl)-3-methoxybenzene was performed following an established protocol.^99^ To a solution of sodium azide (130 mg, 2 mmol) in acetone/water (3/1, v/v, 5 mL), 1 mmol of an alkyl bromide was added. The reaction was stirred for 2 h at room temperature. Then, 5 mL of H_2_O was added and extracted with ethyl acetate (3 × 20 mL). The organic layers were dried with anhydrous Na_2_SO_4_, filtered, and solvent-evaporated, and the product was obtained in quantitative yields (>99%).

### Aryl Azides

The synthesis of *p*-nitrophenylazide and *p*-methoxyphenylazide was performed following an established protocol.^100^ To a mixture containing arylamine (2.5 mmol), ethyl acetate (5 mL), and water (2.5 mL) was added concentrated hydrochloric acid (1.4 mL) at 0 °C for 10 min. Then, a solution of NaNO_2_ (0.85 equiv) in water (1.5 mL) was dropwise added for 5 min. Upon completion of the addition, the reaction mixture was stirred for 30 min at 0 °C. A solution of sodium azide (0.85 equiv) in water (1.5 mL) was subsequently added over a period of 5 min. After stirring at 0 °C for 30 min, the reaction mixture was diluted with water (8 mL) and extracted with ethyl acetate (3 × 8 mL). The combined organic layer was washed with dilute sodium hydroxide solution 1 M (3 × 10 mL), with water, dried over anhydrous sodium sulfate, and concentrated on a rotary evaporator. The crude compound was purified by column chromatography on silica gel, eluting with ethyl acetate/*n*-hexane as the eluent (9:1 vv), obtaining yields of 92% for *p*-nitrophenylazide and 60% for *p*-methoxyphenylazide.

### Catalysis Protocol for *p*-Nitrophenylazide Reduction

The solvent, acetonitrile or methanol (4.00 mL), containing 1 mol % (mol % of catalyst = (no. of moles of catalyst × 100)/(number of moles of catalyst + number of moles of limiting reagent)) of the copper complex, was deaerated in a 50 mL Schlenk flask for 30 min. After that, 28.56 mg of *p*-nitrophenylazide (0.17 mmol, 1 equiv) was added, followed by the addition of sodium ascorbate (17.23 mg, 0.5 equiv). After that period, 2,2,2-trifluoroethanol (TFE) or distilled water was added. The flask was heated at 80 °C for 1 h, and after that, the reaction was extracted with ethyl acetate/water. The organic phase was dried with Na_2_SO_4_(*s*), filtered, and the solvent was removed through rotary evaporation. The product conversion was analyzed via ^1^H NMR. All reactions were performed at least twice (Table 1).

### Catalysis Protocol for Reduction of Aldehydes and Other Azides

A 4 mL portion of methanol containing 1 mol % of the copper complex was deaerated in a 50 mL Schlenk flask for 30 min. After that, 0.18 mmol of the substrate (1 equiv) was added, followed by the addition of 2.2 equiv of sodium ascorbate dissolved in 1.2 mL of deaerated water. The flask was heated at 80 °C for 12 h, and after that, the reaction was extracted with ethyl acetate/water. The organic phase was dried with Na_2_SO_4_(*s*) and filtered, and the solvent was removed through rotary evaporation. The product conversion was analyzed via ^1^H NMR. All reactions were performed at least twice.

### Calculation Protocol

All quantum chemical calculations were conducted applying the xtb 6.6 (GFNn-xTB) and ORCA 5.0.4 (DFT) program packages. Initial guess geometries from the experimental data were used. These molecules have numerous degrees of rotation, and standard geometry optimization algorithms may yield a relatively high-energy conformer. We use the metadynamics package Conformer Rotamer Ensemble Sampling Tool (CREST), driven by the semiempirical density functional tight binding theory GFN1-xTB to find out the lowest-energy conformer. The lowest conformer found was used as the initial structure for the DFT method.

DFT geometry optimizations were conducted using a composite scheme with default convergence criteria and the SlowConv keyword, as implemented in ORCA, employing the Ahlrichs’ triple-ζ def2-TZVP basis set and PBE0 exchange-correlation functional. The resolution of the identity approximation for Coulomb (RI-J)­[] and exchange (RI-JK)­[] integrals in conjunction with matching auxiliary basis sets (def2/JK option) was applied to speed up the DFT calculations. The D4 London dispersion correction scheme was applied.

Solvation effects were considered by the implicit solvation Conductor-Like Polarizable Continuum Model at the DFT level­[] and the generalized born (GB) model with a solvent-accessible surface area (SASA) termed as GBSA,[] as implemented in xtb for GFN1-xTB. In this context, the solvents are the acetonitrile (ε = 36.6) and methanol (ε = 32.6).

### Characterization Methods

**FTIR:** Fourier transform infrared (FTIR) spectra were obtained in KBr pellets using a Bomen-Michelson FT spectrometer model MB-102. All measurements were obtained in intervals of 400 and 4000 cm^–1^.

**UV–vis:** Electronic spectra were recorded on an HP – Hewlett-Packard 8452 A spectrophotometer. The samples were analyzed in solution using a quartz cell with a maximum volume of 1 mL and an optical path of 1.0 cm. Values of molar absorptivity, ε, were calculated using the maximum absorbance value of the bands from the Lambert–Beer law (ε = *A*/*bC*), in which *A* = absorbance, *b* = optical path, and *C* = concentration in mol L^–1^.

**EPR:** Measurements were recorded at room temperature (296 K) and liquid nitrogen temperature (77 K) for the Cu complexes and only at room temperature (296 K) for DMPO adducts. For the measurements, an EPR equipment model Varian E109, X band, was used, featuring a rectangular cavity and modulation at 100 kHz. The parameters for the measurements were as follows: microwave power of 20 mW, modulation amplitude of 0.4 mT (0.1 mT) peak-to-peak with an automatic gain for each sample, field scanning of 160 mT (10 mT), and 0.064 s, and scanning time for 3 min (or 1 min). To calibrate the magnetic field, an EPR standard was employed (MgO:Cr­(III) g = 1.9797 crystal), and the resonance frequency was measured with a microwave frequency meter.

**Microanalysis:** All microanalyses were performed by the Analytical Central from the Department of Chemistry at UFSCar using EAGER 200 CE equipment.

**Conductivity:** All conductivity measurements were obtained from 1 mM methanolic solutions in the conductivity meter Meter Lab model CDM230. The obtained values were compared with blank solutions (solvent) and the standard electrolytic region.

**Electrochemical measurements:** The electrochemical measurements were performed using an EG&G potentiostat Princeton Applied Research Model 273A/A conventional glass cell with three electrodes was used. The electrodes used were vitreous carbon (0.071 cm^2^), platin and Ag_(s)_/AgCl_(s)_|KCl^–^ (3.5 M) as working, auxiliar, and reference electrodes, respectively. All measurements were performed in methanol (or acetonitrile) containing 0.1 M tetrabutylammonium perchlorate as the electrolyte. To remove dissolved oxygen, argon was purged into the cell for 15 min prior to each scan. The working electrode was polished with 0.05 μm alumina before the experiments, followed by washing with water. The auxiliar and reference electrodes were washed with methanol prior to their addition to the cell.

**NMR:** All ^1^H, ^13^C, and 2D NMR spectra were obtained on a 400 MHz Bruker ARX 9.4 T.

NMR spectra were obtained from solutions of deuterated chloroform (CDCl_3_) or acetone (CD_3_)_2_CO with the residual solvent serving as an internal standard. NMR shifts were reported in parts per million (ppm). Abbreviations for signal multiplicity are as follows: s = singlet, d = doublet, *t* = triplet, q = quartet, *m* = multiplet, dd = doublet of doublet, etc. Coupling constants (*J* values) were calculated directly from the spectra. ^15^N NMR spectra were recorded in methanol-D_4_ and referenced to liquid ammonia. The ^15^N NMR spectra were recorded at 600 MHz using a Bruker 600 spectrometer.

**Mass spectrometry:** The complexes were ionized by electrospray (ESI) and analyzed at the Mass Spectroscopy Emory Core.

**XPS**: XPS analysis was performed at the Brazilian Nanotechnology National Laboratory (LNNano), part of the Brazilian Centre for Research in Energy and Materials (CNPEM), a private nonprofit organization under the supervision of the Brazilian Ministry for Science, Technology, and Innovations (MCTI).

## Conclusions

Four copper complexes were synthesized based on derivatizations of l-proline to obtain complexes bearing Schiff base ligands. These complexes were employed in the reduction reaction of 4-nitrophenylazide using methanol and acetonitrile as solvents and sodium ascorbate as an electron donor. We observed that the best reaction yields were obtained when the copper complexes were in the Cu­(II) oxidation state, which contradicted the copper-nitrene formation. Moreover, EPR experiments backed up by computational calculations evidenced that neither the azide nor the amine could coordinate to the copper complexes. A more in-depth analysis of the EPR using DMPO as a radical scavenger, evidenced that the copper complexes present Cu-phenoxyl radicals. These radicals are easily reduced by ascorbate, generating the ascorbyl radical in solution, which was calculated to be the reductant of the azide. The ascorbyl formation was strongly dominated by a solvent influence, in which methanol was the best solvent for the reduction of the azide. The ascorbyl radicals generated in such reactions were shown to reduce a broader scope of azides and some aldehydes, evidencing a new mechanism of copper/ascorbate reducing reactions. Thus, in this study, we evidenced the influence of the solvent in substrate reduction, evidencing the ascorbyl radical as an important species in the mechanism.

## Supplementary Material



## Data Availability

The data that support the findings of this study are available in the manuscript and SI. Other data not available in the manuscript can be obtained by contacting the corresponding author, CGCMN, upon reasonable request.

## Notes

The methods section with the ligand synthesis protocol, ^1^H NMR and their spectroscopic characterizations (Figures S1–S15). FTIR of complexes (Figure S16), UV–vis (Figure S17), HRMS (Figures S18–S21), CV scans (Figures S22–S24), spectroelectrochemical experiment (Figure S25), EPR (Figures S26, S30, S31, and S34; Table S1); ^1^H NMR of the reaction under different solvents (Figure S27); HRMS of the reaction (Figures S28 and S29); reaction with ferrocyanide (Figure S32); XPS (Figure S33); 1H NMR of the reaction between the complex and ascorbate in acetonitrile (Figure S35); theoretical calculations (Figure S36, Table S2); X-ray crystal structure data (Table S3); 1H NMR of the reduction of different substrates (Figures S37–S48) (PDF)
