# Supplementary material for: Ascorbyl Radicals as Reducing Agents in Copper-Catalyzed Redox Reactions
Source: Inorg Chem. 2025 Sep 12;64(46):22563–80. doi: 10.1021/acs.inorgchem.5c00934 (PMC12648647; doi:10.1021/acs.inorgchem.5c00934)
Supplement: Supplementary file 1 [file ic5c00934_si_001.pdf]

Ascorbyl radicals as reducing agents in copper-catalyzed redox reactions

Caio Bezerra de Castro, Gabrielle Conciani, Everton M. da Silva, Joao Honorato, Walber Gonçalves. Guimarães Júnior, André Farias de Moura , Javier Ellena, Arlene G. Corrêa, Otaciro Rangel Nascimento, Caterina G.C. Marques Netto

Supporting Information

1.Methods 3
1.2 Synthesis 3
1.2.1 Synthesis of the ligands and characterization 3
1.2.2 Synthesis of p_4L_2 4
1.2.3 Synthesis of the ligands L_3H and L_4H 5
2. Spectra 6
Figure S1. 6
Figure S2 7
Figure S3. 7
Figure S4. 8
Figure S5. 8
Figure S6. 9
Figure S7. 10
Figure S8. 11
Figure S9. 11
Figure S10. 12
Figure S11. 13

Figure S12. 13
Figure S13. 14
Figure S14. 14
Figure S15. 15
Figure S16. 16
Figure S17. 17
Figure S18. 18
Figure S19. 19
Figure S20. 20
Figure S21. 21
Figure S22. 22
Figure S23. 22
Figure S24. 23
Figure S25. 23
Figure S26. 25
Table S1: 26
Figure S27...27
Figure S28. 27
Figure S29. 28
Figure S30. 29
Figure S31. 29
Figure S32. 30
Figure S33. 31
Figure S34. 32
Figure S35. 33
Figure S36 34
Table S2 – 34
Table S3. 35
Figure S37 37
Figure S38 37
Figure S39 38
Figure S40 38
Figure S41 39

Figure S42 39
Figure S43 40
Figure S44. 40
Figure S45 41
Figure S46 41
Figure S47 42
Figure S48 42

1.Methods

1.2 Synthesis

1.2.1 Synthesis of the ligands and characterization

Ligands L_1H, L_2H, L_3H and L_4H were synthesized following Scheme S1. The synthesis and characterization of p_3L_1, p_4L_1 [1] and p_3L_2 [2]were already described by us. The synthesis of ligands L_1H and L_2H were described elsewhere. [1]. Despite this, the characterization data for L_1H will be described as it is the first report of this molecule in the literature.

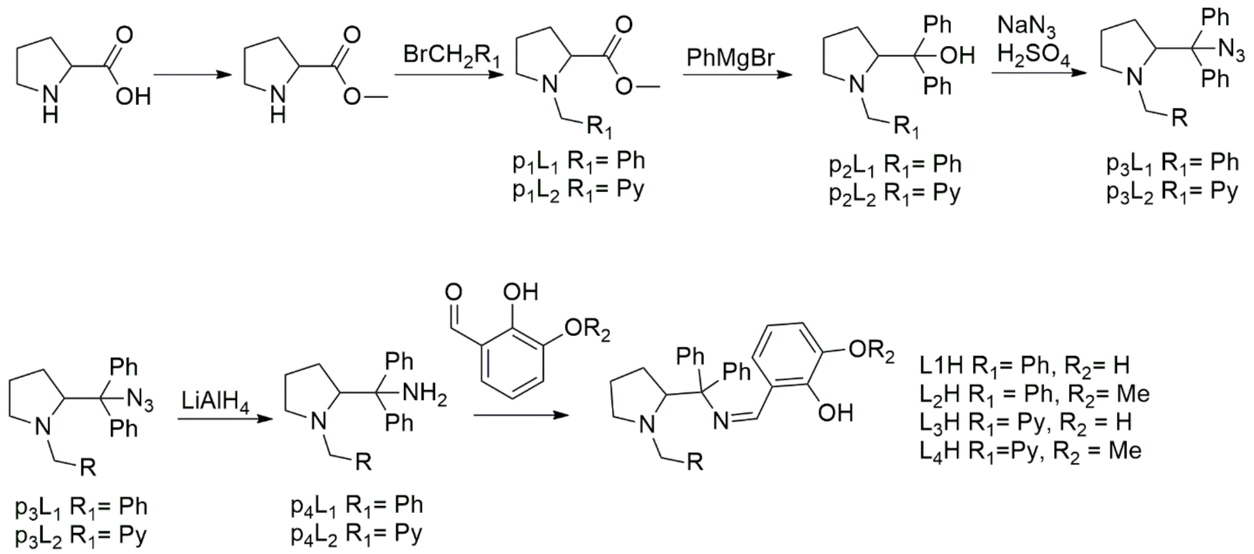

Scheme S1. Scheme of the synthesis of the ligands. All ligands were synthesized starting from L-Proline esterification with $SOCl_2$ in methanol, followed by alkylation with a bromo-derived alkane, generating the precursors p_1L_1 and p_1L_2. These precursors were used in a Grignard reaction to obtain p_2L_1 and p_2L_2. The reaction with sodium azide in acidic media generated their corresponding azides (p_3L_1 and p_3L_2), which upon reduction with $LiAlH_4$ resulted in the amines p_4L_1 and p_4L_2. The reaction between these amines with an aldehyde resulted in the ligands L_1H, L_2H, L_3H and L_4H.

1.2.2 Synthesis of p_4L_2

Compound p_4L_2 was synthesized from 544 mg of p_3L_2 in 5 mL of anhydrous THF in a Schlenk flask under argon atmosphere. After solubilization of the azide, the solution was cooled to 0 °C and 233 mg of $LiAlH_{4(s)}$ (5,89 mmol) were added. The reaction proceeded under reflux for 8 hours and quenched with a saturated solution of $Na_2SO_{4(aq)}$ until complete consumption of the hydride. The mixture was filtrated under vacum and the solution was extracted with dichloromethane (3x 10 mL). The organic phases were combined, dried with $MgSO_{4(s)}$, filtered and dried under vacuum and rotatory evaporation. To the obtained compound a 1:1 hexane:diethyl ether solution was added and a white solid formed. Yield: 40%.

^{1}H NMR (400 MHz, $CDCl_3$): δ 8,42 (d, J = 4,8 Hz, 2H); 7,62 (t, J = 15,6, 7,6 Hz, 1H); 7,54 (t, J = 18,4, 8,4 Hz, 2H); 7,32 (t, J = 15,2, 7,6 Hz, 3H); 7,23 (t, J = 15,6, 8,0 Hz, 2H); 7,18 (t, J = 14,8, 7,2 Hz, 2H), 7,13 – 7,03 (m, 2H); 4,04 (dd, J = 10,0, 2,8 Hz, 1H); 3,32 (d, J = 14,4 Hz, 1H); 3,12 (d, J = 13,6 Hz, 1H); 2,99 – 2,91 (m, 1H); 2,53 (bs, 2H); 2,47 – 2,37 (m, 1H); 2,28 – 2,15 (m, 1H); 1,86 – 1,76 (m, 1H); 1,76 – 1,67 (m, 1H) e 1,63 – 1,48 ppm (m, 1H).

1.2.3 Synthesis of the ligands L_3H and L_4H

In a round bottom flask 45 mg of p_4L_2 (0.13 mmol) and 0.5 mg of $NaOH_{(s)}$ (10 mol%) were dissolved into 2 mL of ethanol. After 30 minutes of sturring at room temperature, 1.05 equivalents of the salicylaldehyde derivative for each ligand was added (19 mg of 2,3-dihydroxibenzaldehyde for L_3H and 22 mg of 2-hydroxi-3- methoxylbenzaldehyde for L_4H). Then, 9 mg of $Na_2SO_{4(s)}$ (0.5 eq.) were added and the reaction was stirred at 40 °C for 24 hours. After that period, the reaction mixture was extensively washed with ethanol to obtain a yellow solid, which was purified by silica gel chromatographic column using methanol: dichloromethane (2.5:97.5) as eluent. Yield L_3H: 45% and L_4H: 40%.

L_3H

FTIR (cm^{-1}, KBr): 3553, 3476, 3412, 3238, 3057, 3028, 2964, 2872, 2804, 1626, 1595, 1545, 1491, 1466, 1446, 1358, 1273, 1235, 1207, 1190, 1030, 1005, 995, 850, 748, 703, 623, 472, 405 cm^{-1}.

UV-Vis (nm, methanol): 254 (10826 mol^{-1} L cm^{-1}); 262 (10604 mol^{-1} L cm^{-1}); 298 (5480 mol^{-1} L cm^{-1}) e 430nm (2283 mol^{-1} L cm^{-1}).

^{1}H NMR (400 MHz, $CDCl_3$): 8.28 (d, *J* = 4.4 Hz, 1H), 7.82 (s, 1H), 7.33 – 7.23 (m, 9H), 7.15 – 6.96 (m, 5H), 6.84 (dd, *J* = 7.4, 1.6 Hz, 1H), 6.49 (dd, *J* = 8.2, 1.6 Hz, 1H), 6.37 (t, *J* = 15.6, 7.6 Hz, 1H), 4.12 (dd, *J* = 9.4, 2.4 Hz, 1H), 3.39 (d, *J* = 14.8 Hz, 1H), 3.18 (d, *J* = 14.8 Hz, 1H), 2.89 (t, *J* = 14.4, 8.0 Hz, 1H), 2.29 – 2.16 (m, 2H), 1.74 – 1.63 (m, 1H), 1.52 – 1.41 (m, 1H), 1.20 – 1.05 ppm (m, 1H).

^{13}C NMR (100 MHz, $CDCl_3$): 163.02, 162.73, 160.25, 148.19, 130.20, 130.01, 128.80, 128.40, 128.13, 127.98, 127.40, 122.89, 122.79, 121.70, 114.76, 77.36, 75.94, 71.56, 63.18, 55.43, 30.77, 24.44 ppm.

L_4H

FT-IR (cm^{-1}, KBr): 3547, 3477, 3412, 3236, 3055, 2964, 2872, 2804, 1620, 1593, 1568, 1491, 1474, 1468, 1460, 1446, 1271, 1252, 1080, 1007, 993, 984, 839, 781, 748, 738, 703, 621, 476, 401 cm^{-1}.

UV-Vis (nm, methanol): 244 (12148 mol^{-1} L cm^{-1}); 262 (9187 mol^{-1} L cm^{-1}); 296 (6645 mol^{-1} L cm^{-1}) e 424 nm (1295 mol^{-1} L cm^{-1}).

^{1}H NMR (400 MHz, $CDCl_3$): 15.25 (bs, 1H), 8.27 (d, *J* = 4.8 Hz, 1H), 7,91 (s, 1H), 7.41 – 7.19 (m, 9H), 7.08 (t, J = 15.2, 7.6 Hz, 2H), 7.04 – 6.95 (m, 2H), 6.84 (dd, *J* = 6.6, 2.8 Hz, 1H), 6.67 – 6.60 (m, 2H), 4.09 (dd, *J* = 9,0, 1,2 Hz, 1H), 3.87 (s, 3H), 3.44 (d, *J* = 14,4 Hz, 1H), 3.30 (d, *J* = 14.4 Hz, 1H), 2.90 – 2.82 (m, 1H), 2.27 – 2.15 (m, 2H), 1.72 – 1.63 (m, 1H), 1.52 – 1.42 (m, 1H), 1.26 – 1.16 ppm (m, 1H).

^{13}C NMR (100 MHz, $CDCl_3$): 164.25, 160.19, 155.15, 149.32, 147.70, 144.55, 142.24, 137.07, 130.33, 128.75, 128.43, 128.04, 127.56, 126.95, 123.86, 123.20, 121.75, 118.05, 117.01, 113.65, 77.69, 72.15, 63.65, 56.07, 55.49, 30.71, 24.59 ppm.

2. Spectra

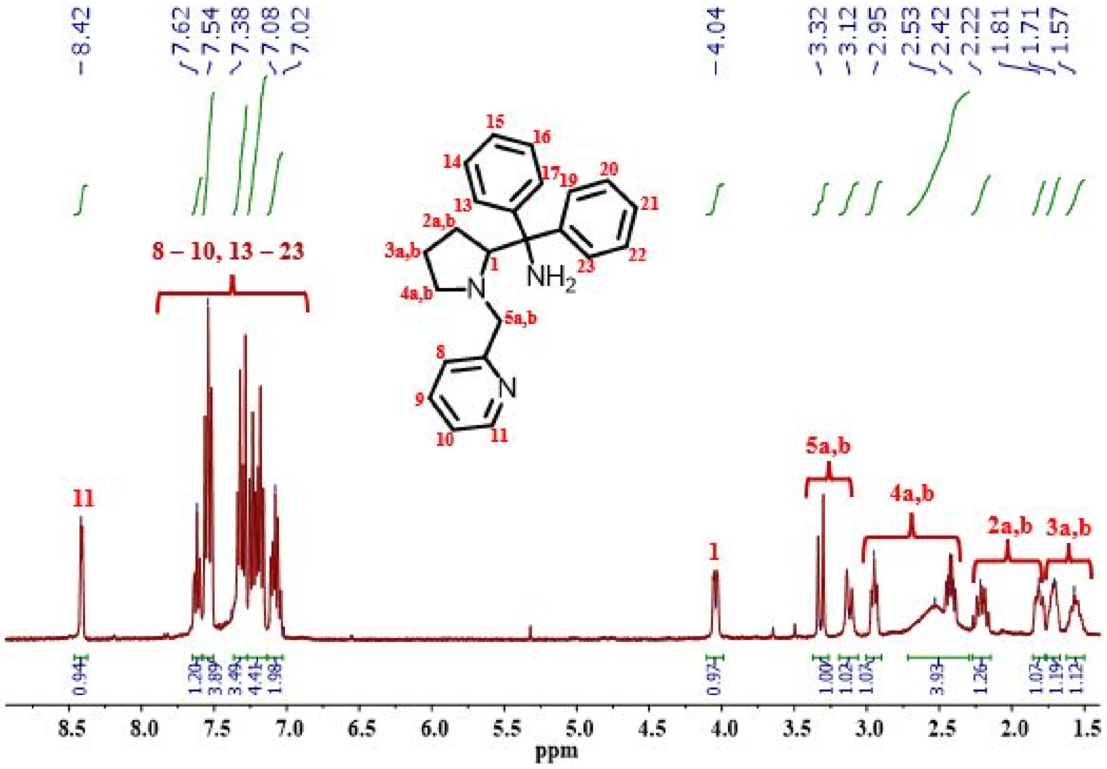

Figure S1. ^{1}H NMR spectrum of p_4L_2 ($CDCl_3$, 400 MHz)

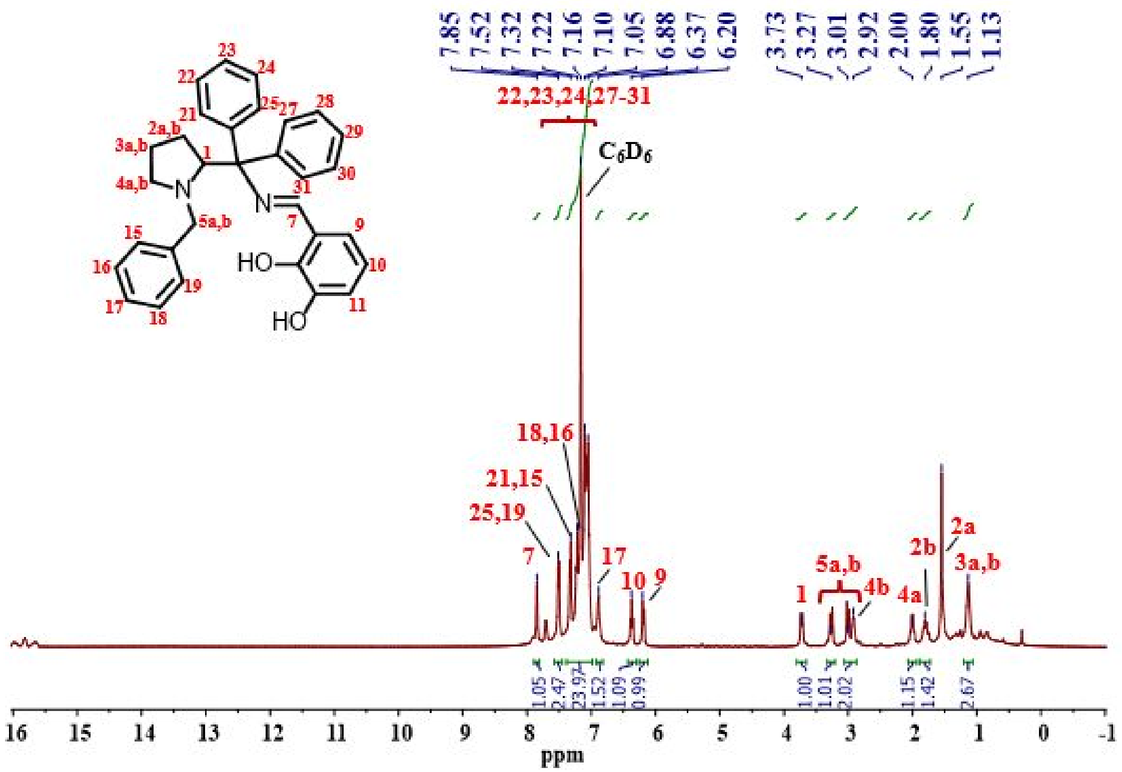

Figure S2. ^{1}H NMR spectrum of L_1H (C_6D_6, 400 MHz)

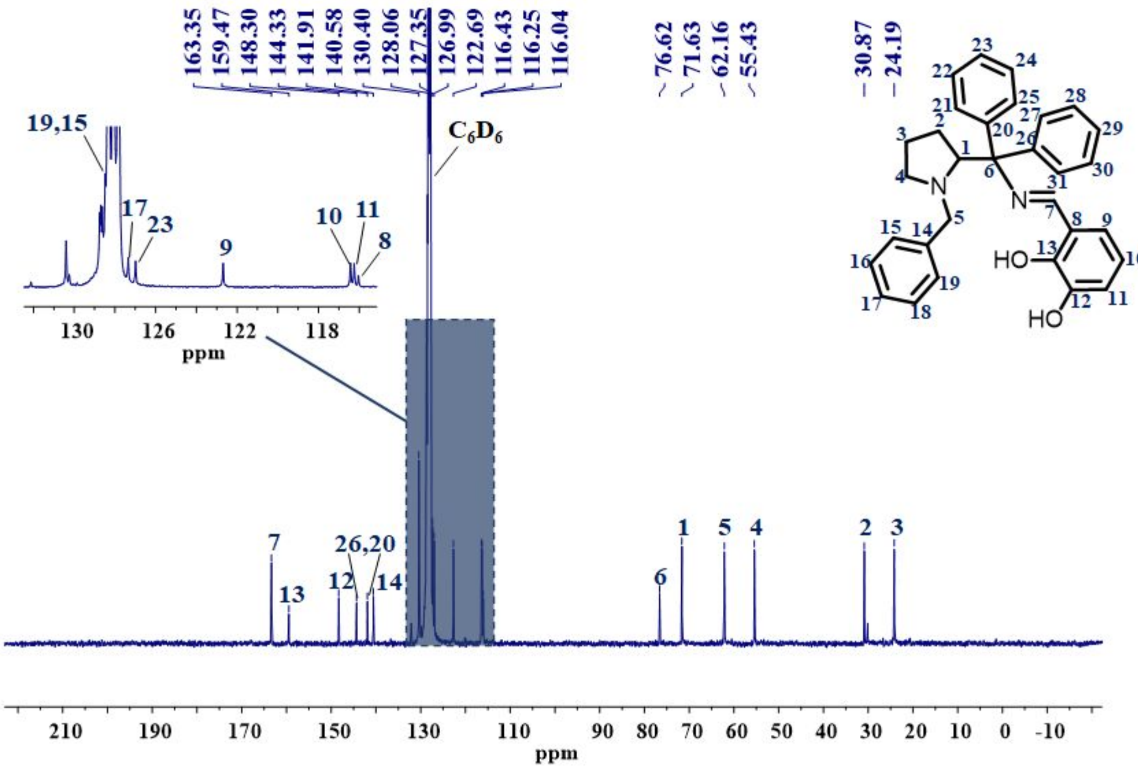

Figure S3. ^{13}C NMR spectrum of L_1H (C_6D_6, 100 MHz).

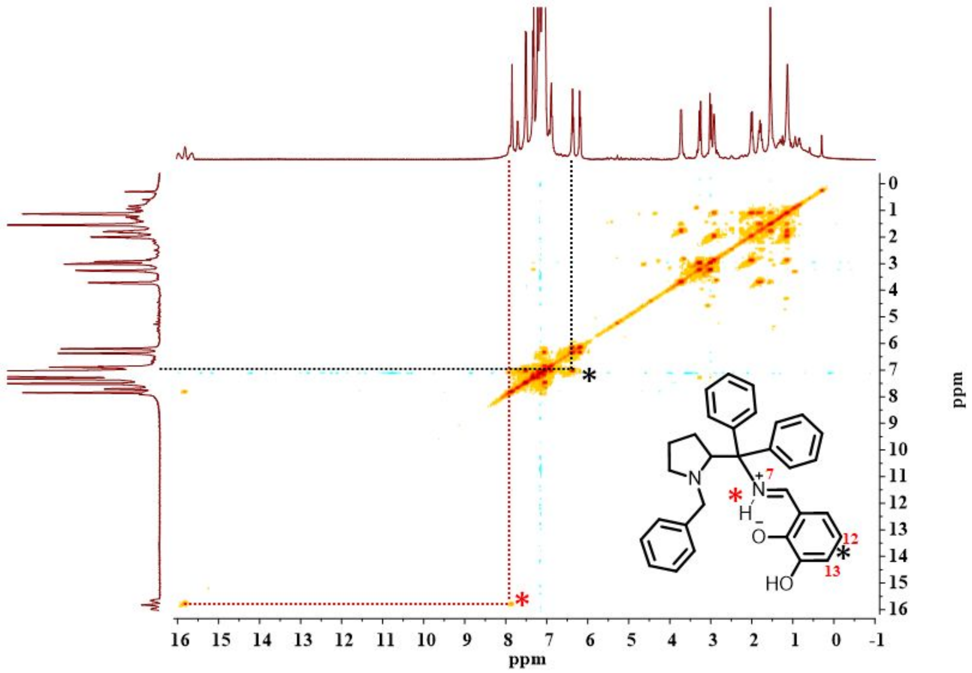

Figure S4. COSY NMR spectrum of L_1H (C_6D_6, 100 MHz). (*) evidences the correlation between proton 10 and the protonated imine and (*) evidences the correlation between hydrogens 12 and 13.

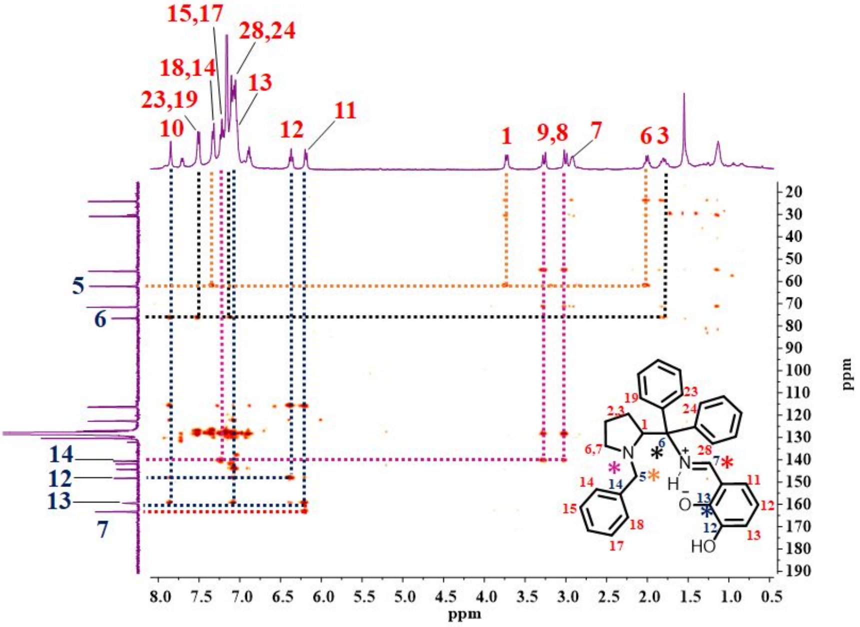

Figure S5. HMBC NMR of L_1H in C_6D_6 400 MHz.

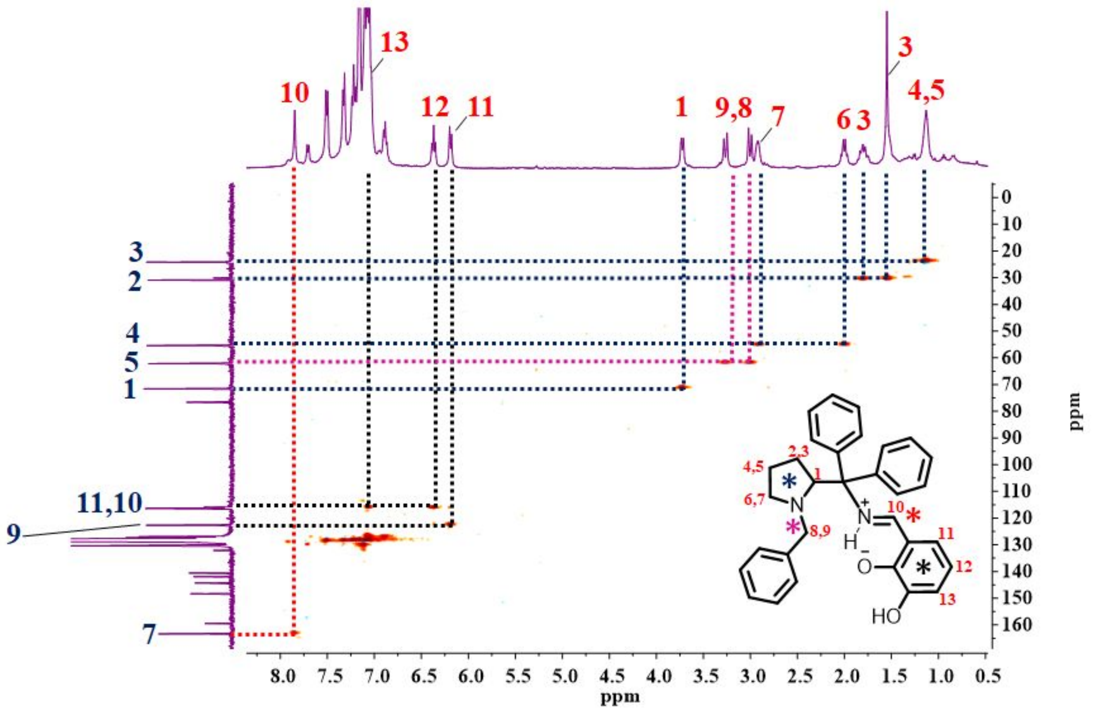

Figure S6. -HSQC of ligand L_1H in C_6D_6 400 MHz.

L_3H

FTIR (cm^{-1}, KBr): 3553, 3476, 3412, 3238, 3057, 3028, 2964, 2872, 2804, 1626, 1595, 1545, 1491, 1466, 1446, 1358, 1273, 1235, 1207, 1190, 1030, 1005, 995, 850, 748, 703, 623, 472, 405 cm^{-1}.

UV-Vis (nm, methanol): 254 (10826 mol^{-1} L cm^{-1}); 262 (10604 mol^{-1} L cm^{-1}); 298 (5480 mol^{-1} L cm^{-1}) e 430nm (2283 mol^{-1} L cm^{-1}).

^{1}H NMR (400 MHz, $CDCl_3$): 8,28 (d, 1H), 7,82 (s, 1H), 7,33 – 7,23 (m, 9H), 7,15 – 6,96 (m, 5H), 6,84 (dd, 1H), 6,49 (dd, 1H), 6,37 (t, 1H), 4,12 (dd, 1H), 3,39 (d, 1H), 3,18 (d, 1H), 2,89 (t, 1 H), 2,29 – 2,16 (m, 2H), 1,74 – 1,63 (m, 1H), 1,52 – 1,41 (m, 1H), 1,20 – 1,05 ppm (m, 1H).

^{13}C NMR (100 MHz, $CDCl_3$): 163,02, 162,73, 160,25, 148,19, 130,20, 130,01, 128,80, 128,40, 128,13, 127,98, 127,40, 122,89, 122,79, 121,70, 114,76, 77,36, 75,94, 71,56, 63,18, 55,43, 30,77, 24,44 ppm.

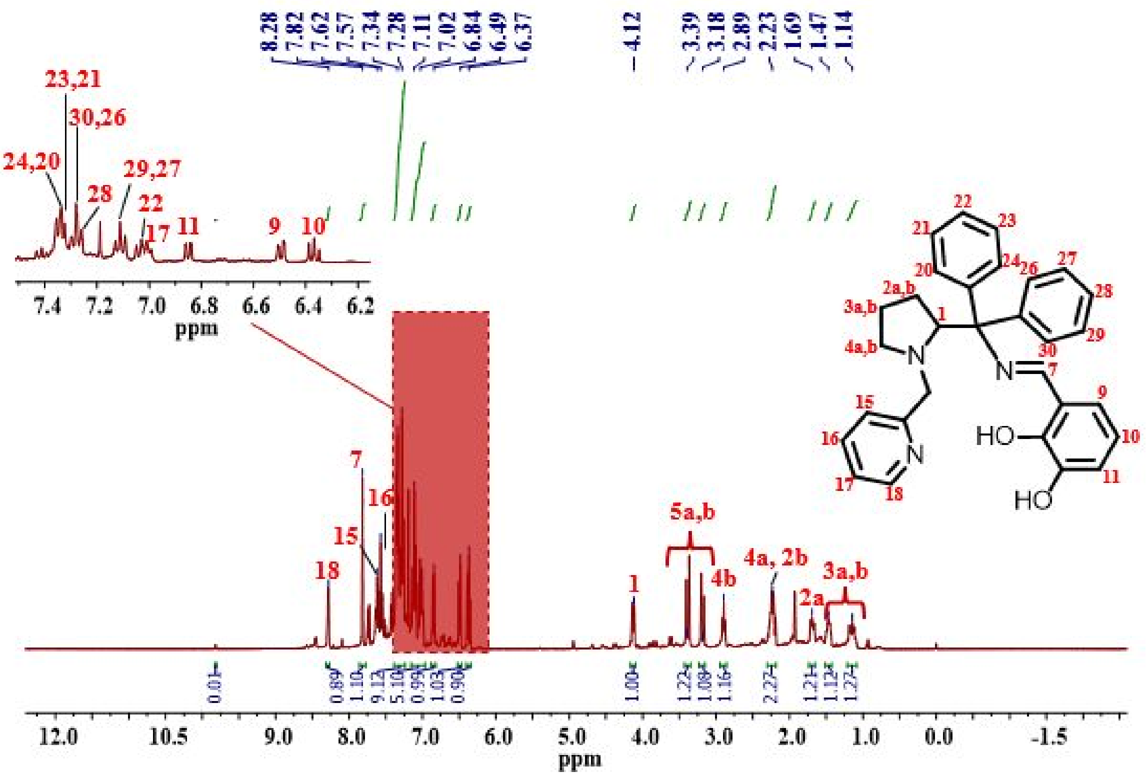

Figure S7. ^{1}H NMR spectrum of L_3H ($CDCl_3$, 400 MHz).

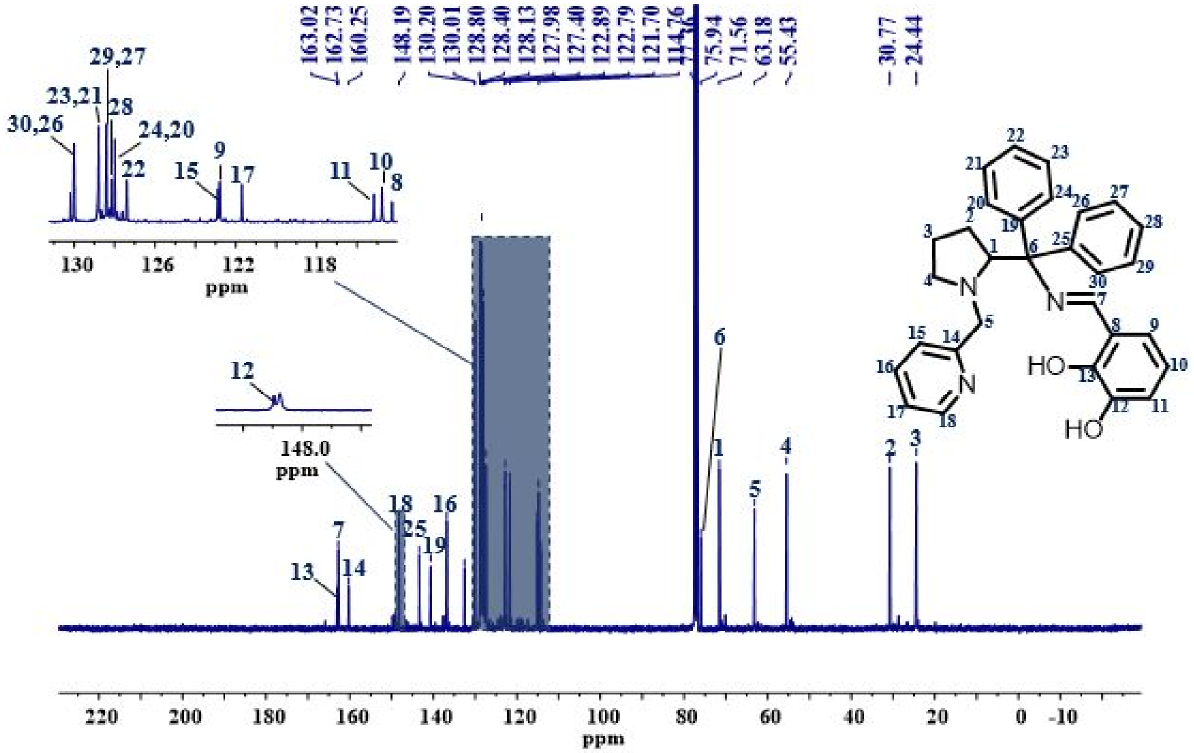

Figure S8. ^{13}C NMR spectrum of L_3H ($CDCl_3$, 100 MHz).

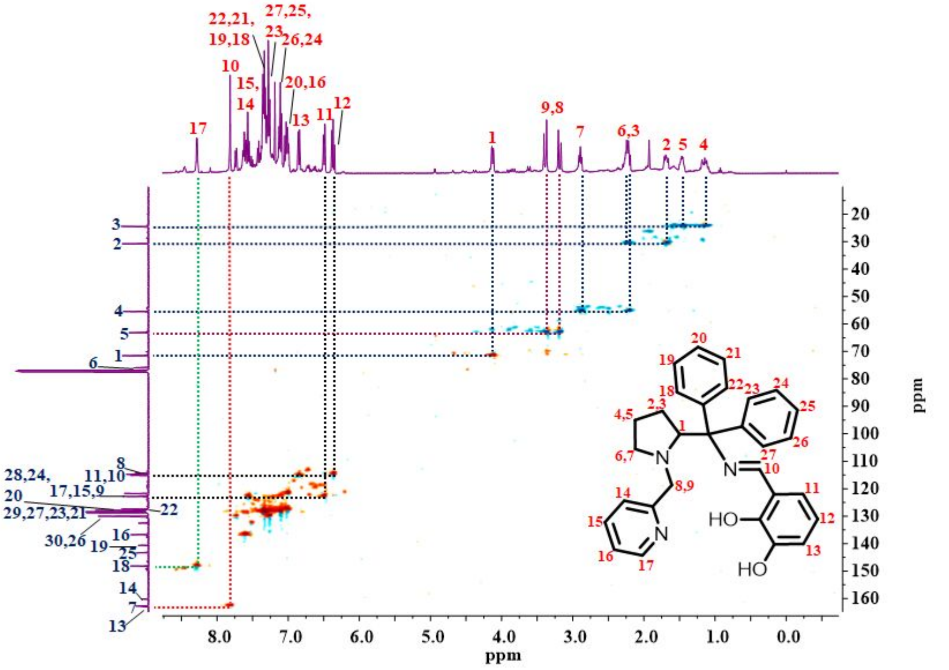

Figure S9. HSQC NMR spectrum of L_3H ($CDCl_3$, 100 MHz).

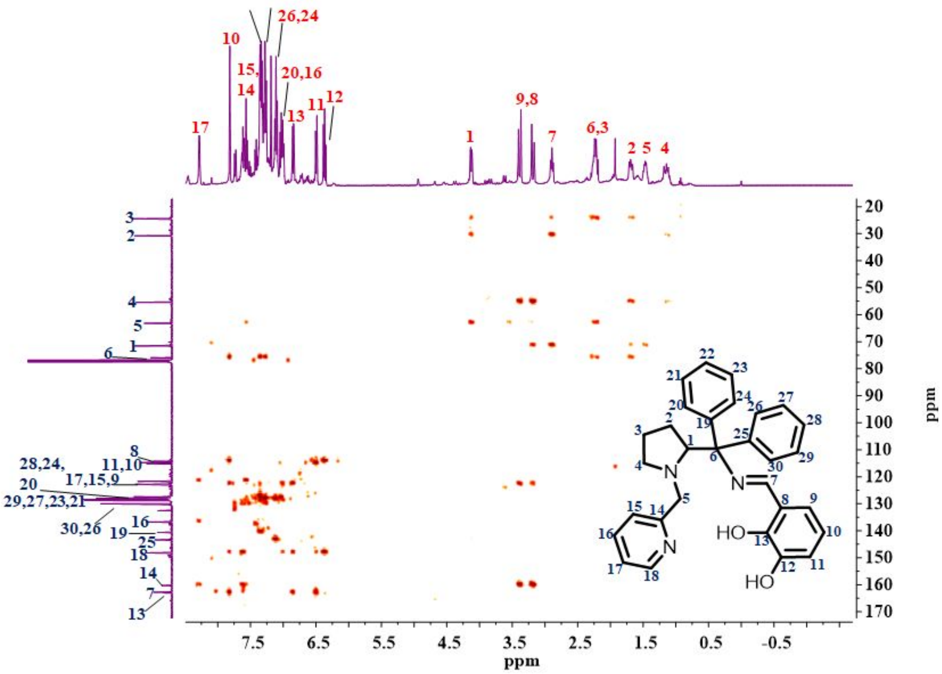

Figure S10. HMBC NMR spectrum of L_3H ($CDCl_3$, 100 MHz).

L_4H

FT-IR (cm^{-1}, KBr): 3547, 3477, 3412, 3236, 3055, 2964, 2872, 2804, 1620, 1593, 1568, 1491, 1474, 1468, 1460, 1446, 1271, 1252, 1080, 1007, 993, 984, 839, 781, 748, 738, 703, 621, 476, 401 cm^{-1}.

UV-Vis (nm, methanol): 244 (12148 mol^{-1} L cm^{-1}); 262 (9187 mol^{-1} L cm^{-1}); 296 (6645 mol^{-1} L cm^{-1}) e 424 nm (1295 mol^{-1} L cm^{-1}).

^{1}H NMR (400 MHz, $CDCl_3$): 15,25 (bs, 1H), 8,27 (d, 1H), 7,91 (s, 1H), 7,41 – 7,19 (m, 9H), 7,08 (t, 2H), 7,04 – 6,95 (m, 2H), 6,84 (dd, 1H), 6,67 – 6,60 (m, 2H), 4,09 (dd, 1H), 3,87 (s, 3H), 3,44 (d, 1H), 3,30 (d, 1H), 2,90 – 2,82 (m, 1H), 2,27 – 2,15 (m, 2H), 1,72 – 1,63 (m, 1H), 1,52 – 1,42 (m, 1H), 1,26 – 1,16 ppm (m, 1H).

^{13}C NMR (100 MHz, $CDCl_3$): 164,25, 160,19, 155,15, 149,32, 147,70, 144,55, 142,24, 137,07, 130,33, 128,75, 128,43, 128,04, 127,56, 126,95, 123,86, 123,20, 121,75, 118,05, 117,01, 113,65, 77,69, 72,15, 63,65, 56,07, 55,49, 30,71, 24,59 ppm.

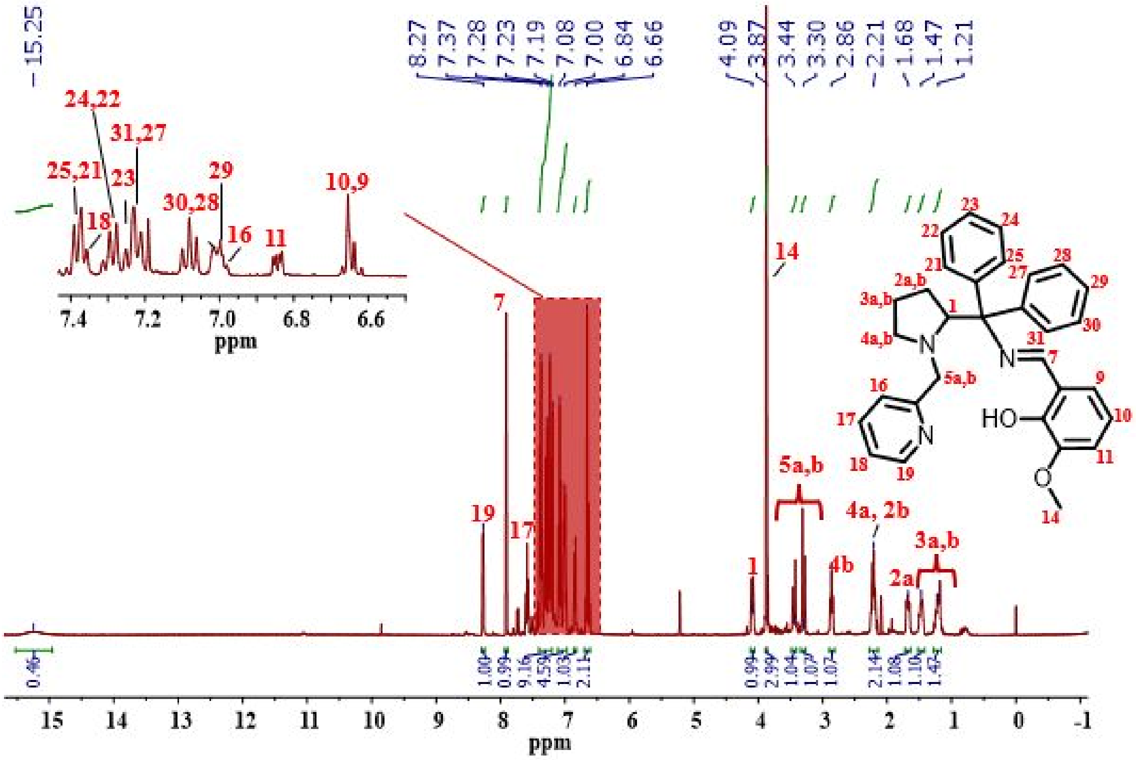

Figure S11. ^{1}H NMR spectrum of L_4H ($CDCl_3$, 400 MHz).

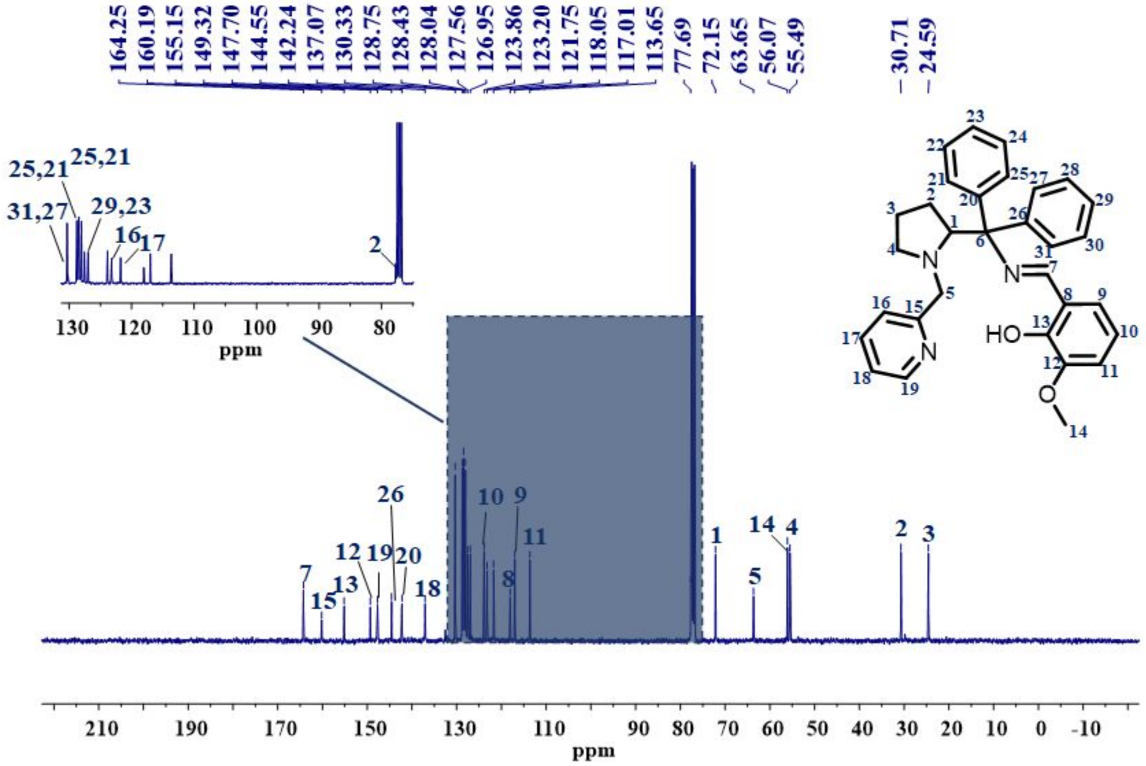

Figure S12. ^{13}C NMR spectrum of L_4H ($CDCl_3$, 400 MHz).

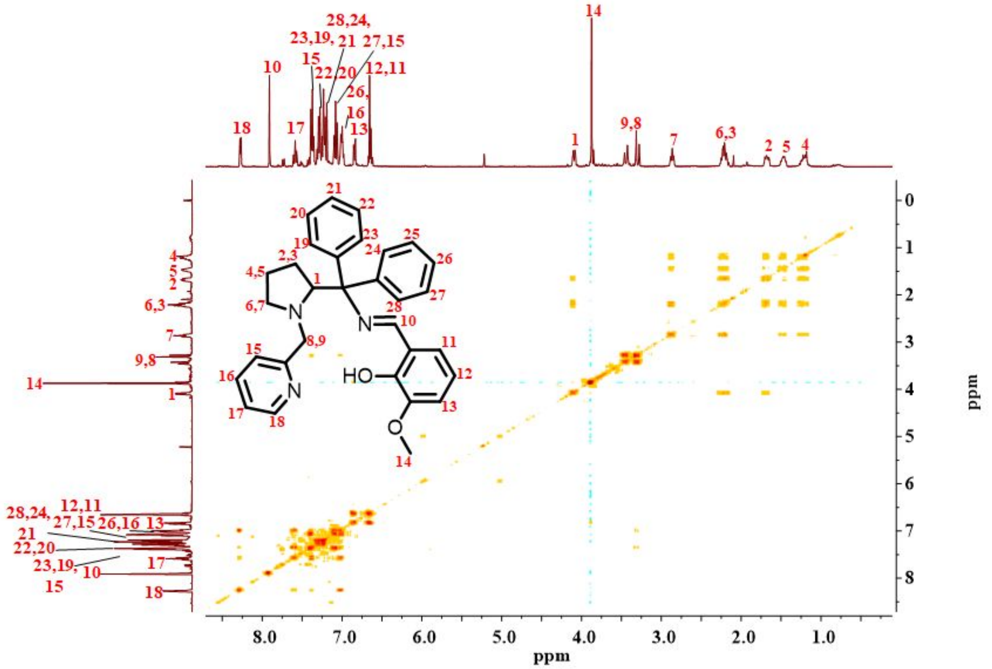

Figure S13. COSY NMR spectrum of L_4H ($CDCl_3$, 400 MHz).

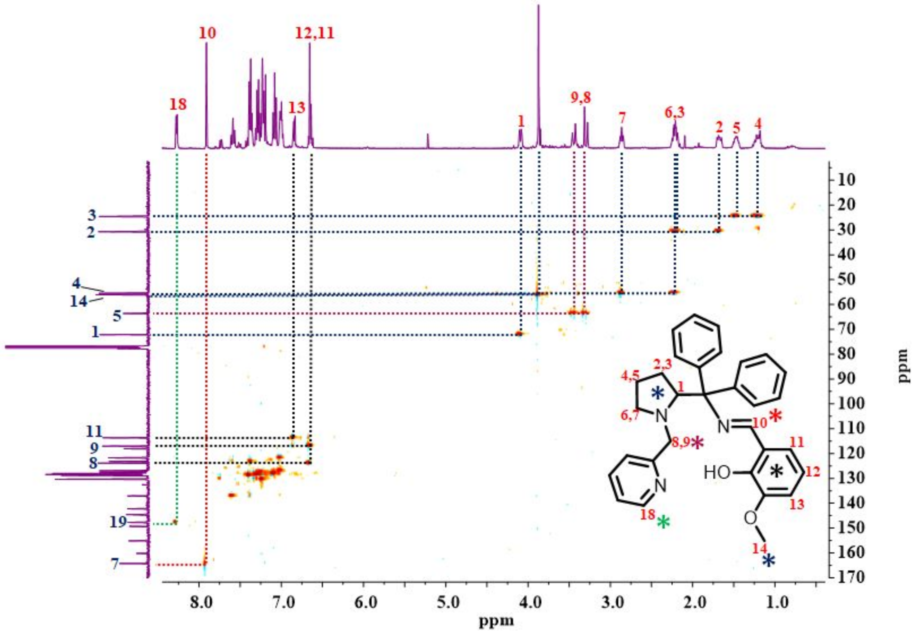

Figure S14. HSQC NMR spectrum of L_4H ($CDCl_3$, 400 MHz).

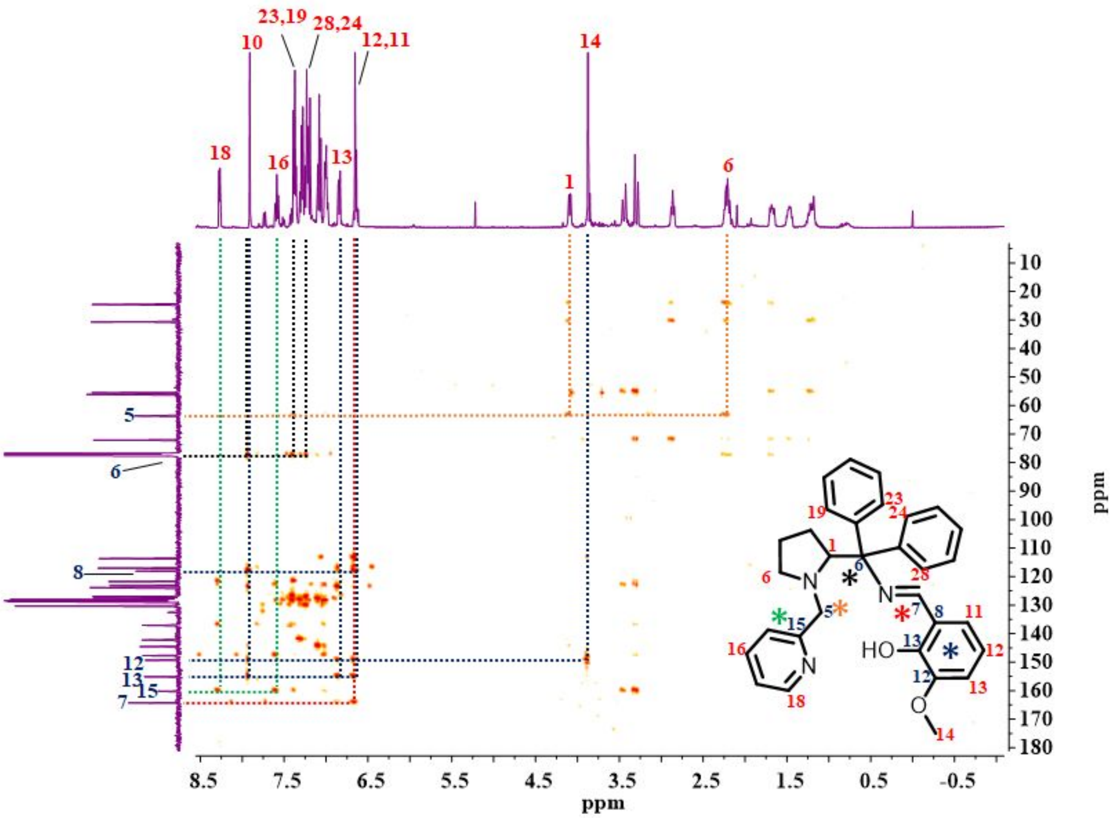

Figure S15. HMBC NMR spectrum of L_4H ($CDCl_3$, 400 MHz).

Characterization of copper complexes (1a – 2b)

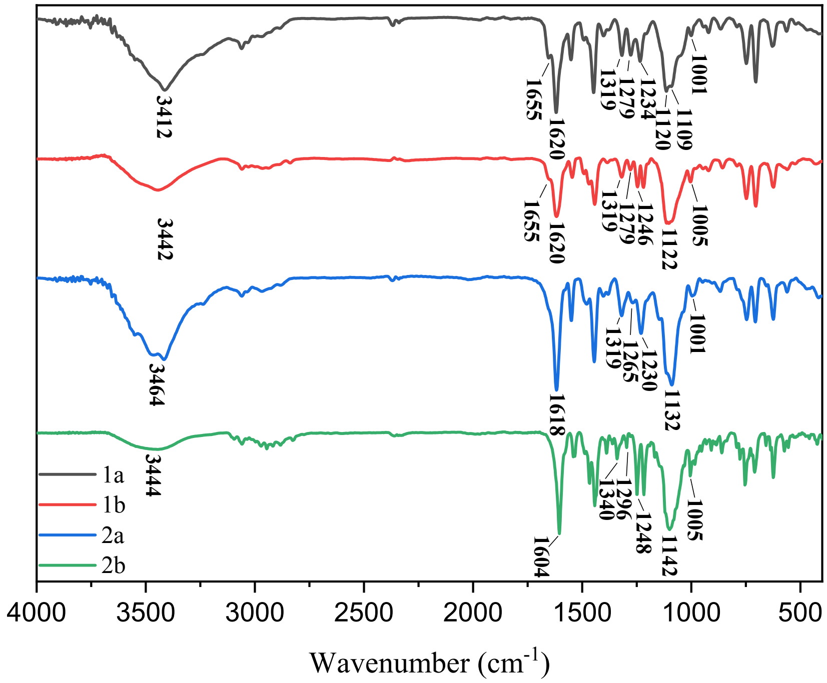

Figure S16. FTIR spectra of copper complexes (**1a** – **2b**) in KBr disk.

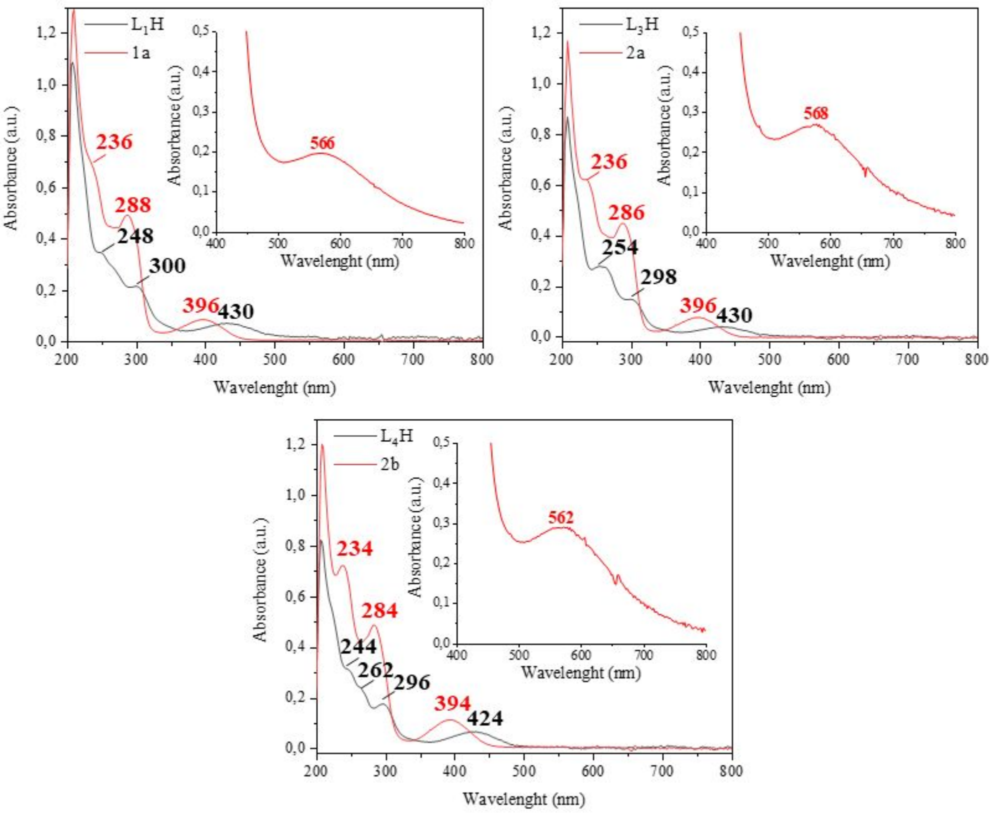

Figure S17. UV-Vis spectra of **1a**, **2a** and **2b** and ligands at same concentration. Inset graph: UV-Vis spectrum of transition d-d band at 1,0x10^{-3} mol L^{-1}.

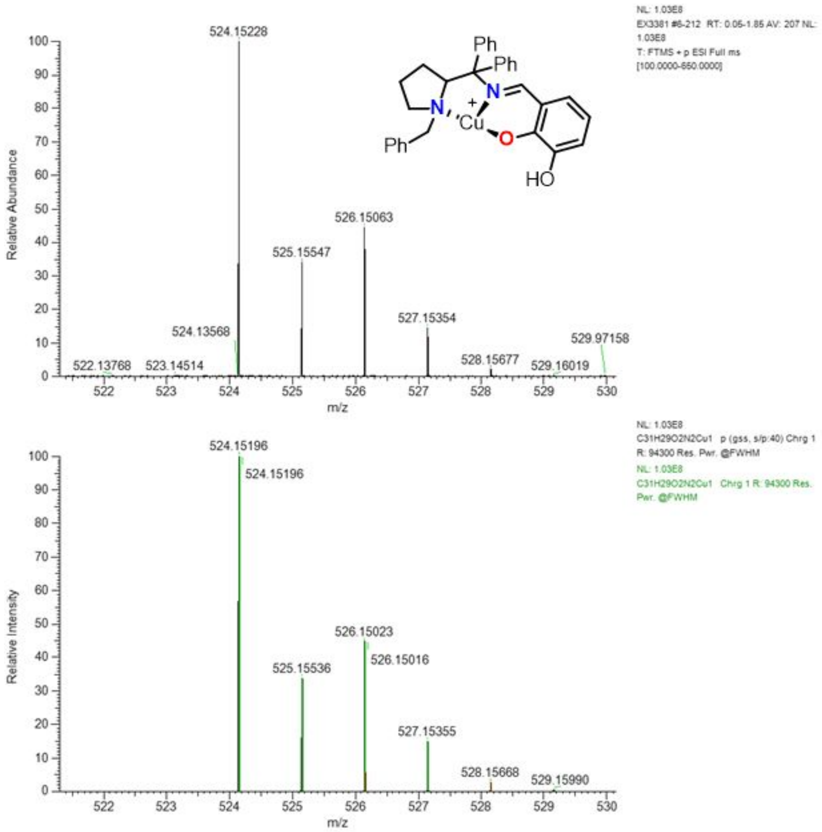

Figure S18. ESI-MS of **1a** complex in positive mode.

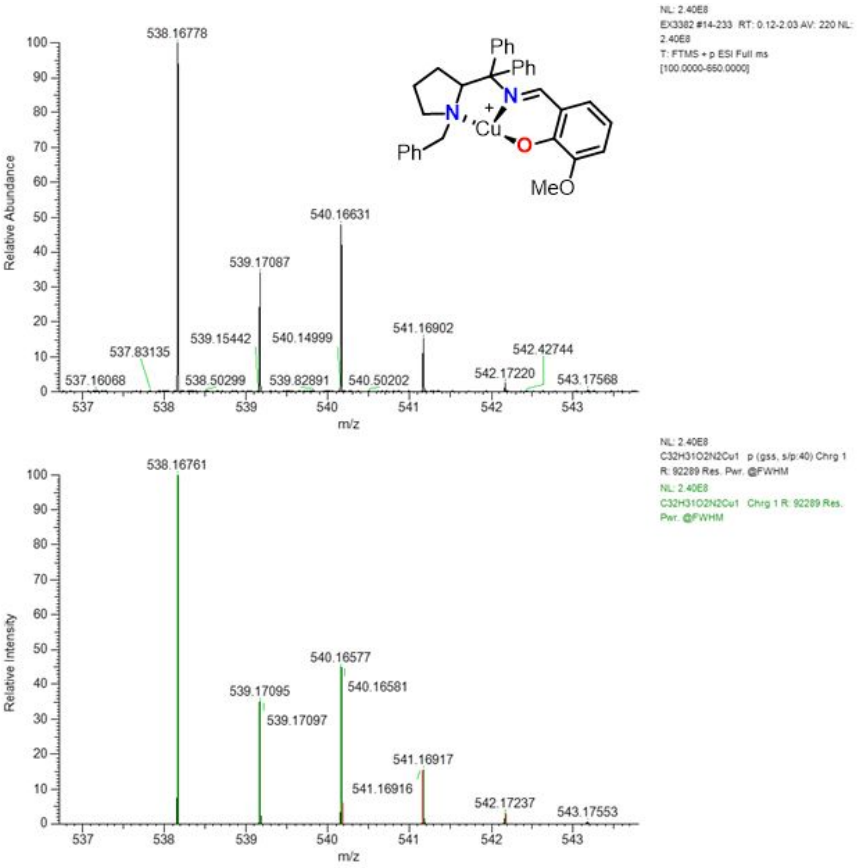

Figure S19. ESI-MS of **1b** complex in positive mode.

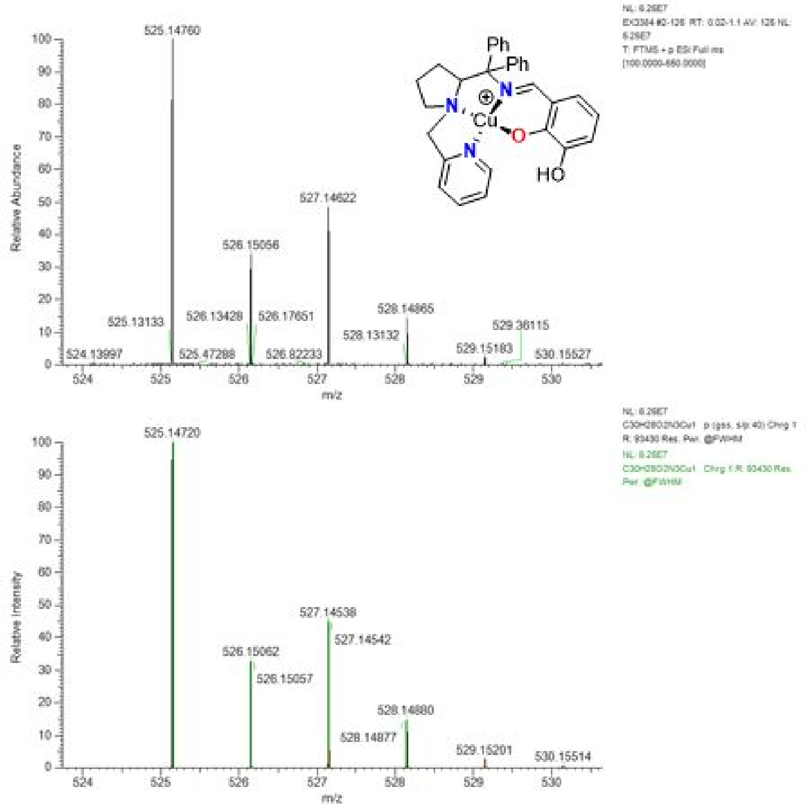

Figure S20. ESI-MS of **2a** complex in positive mode.

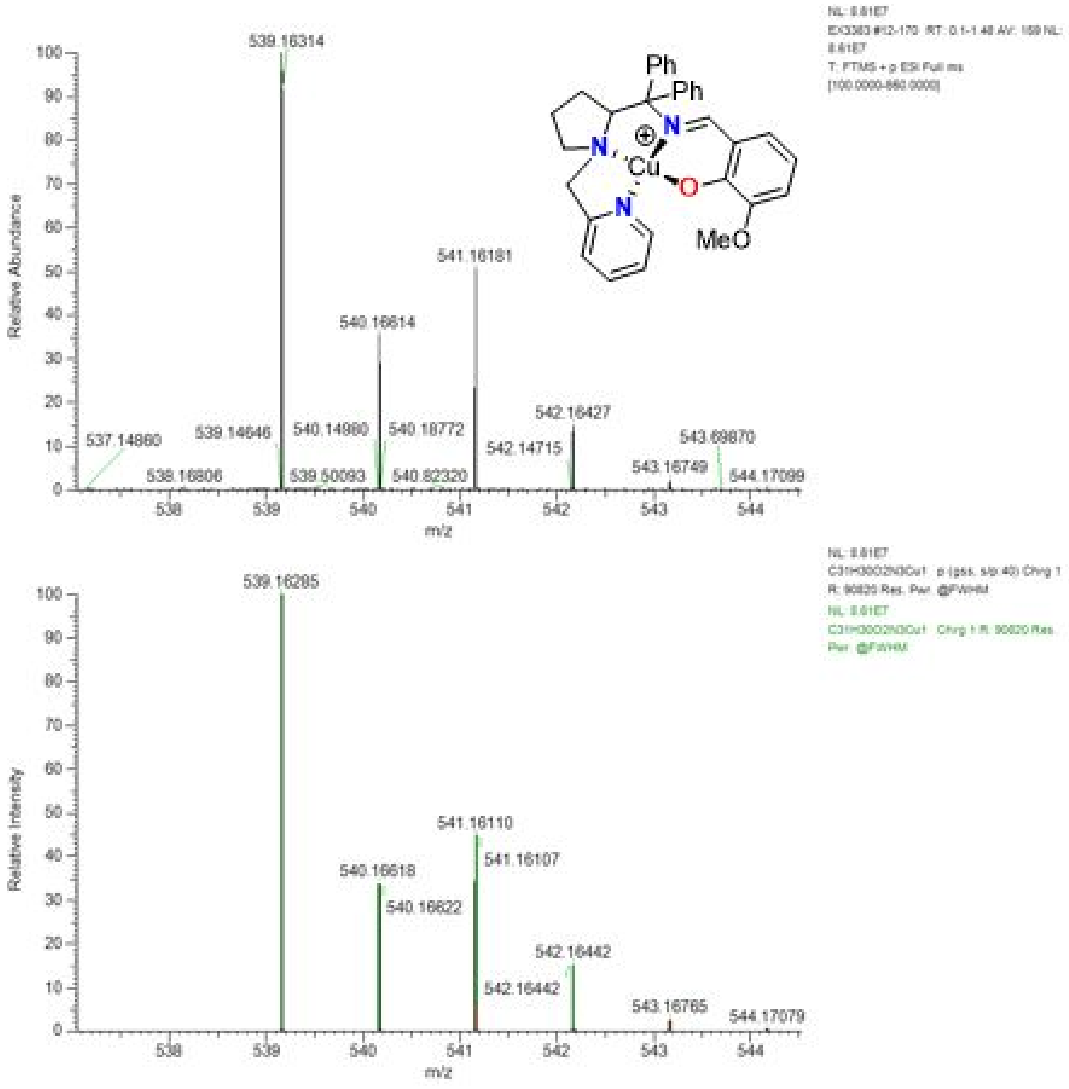

Figure S214. ESI-MS of 2b complex in positive mode.

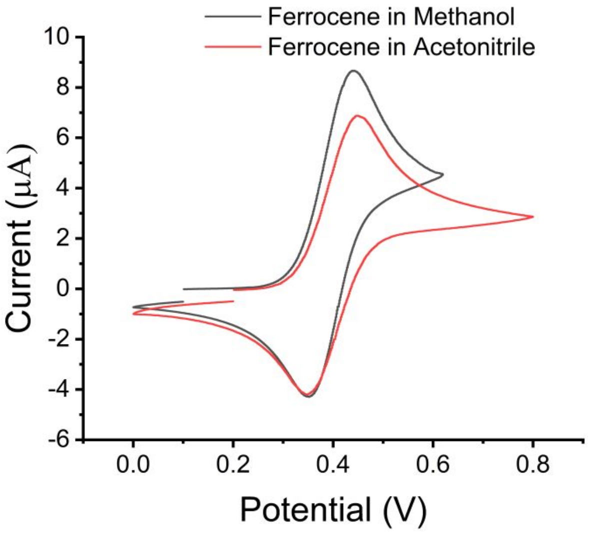

Figure S22. Cyclic voltammetry of ferrocene in acetonitrile and methanol carried out at 100 mV s^{-1} using 0.1 mol L^{-1} of tetrabutylammonium perchlorate (TBAP) as electrolyte. Solutions of the complexes of concentration $1x10^{-3}$ mol L^{-1} were prepared for the measurements. Glassy electrode was used as a working electrode, Pt as counter electrode and Ag/AgCl (3.5 molL^{-1}) was used as a reference.

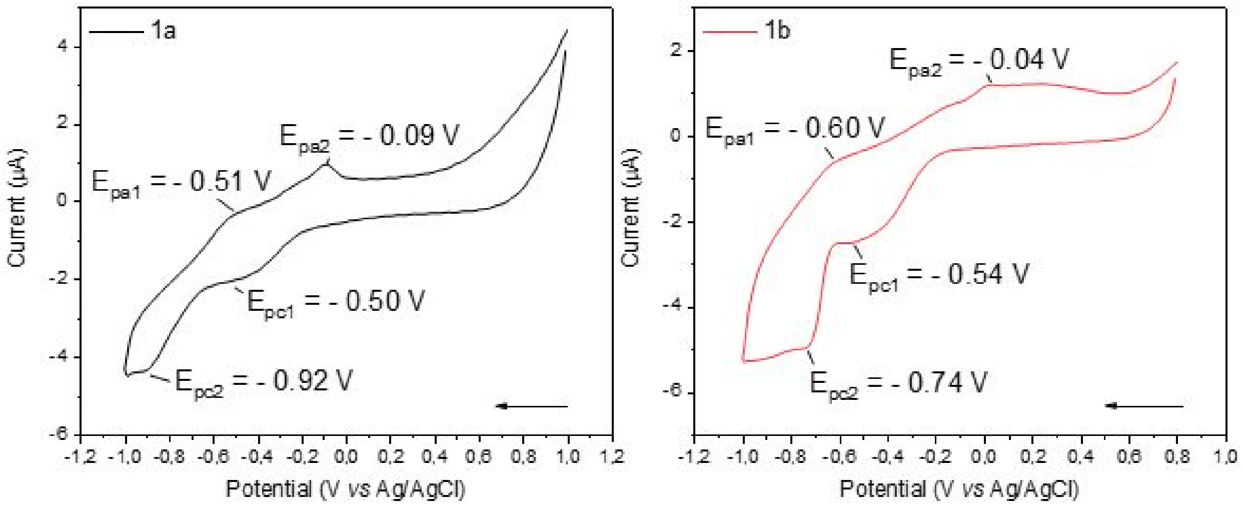

Figure S23. Cyclic voltammetry of 1a and 1b complexes in acetonitrile carried out at 100 mV s^{-1} using 0.1 mol L^{-1} of tetrabutylammonium perchlorate (TBAP) as electrolyte. Solutions of the complexes of concentration $1x10^{-3}$ mol L^{-1} were prepared for the measurements.

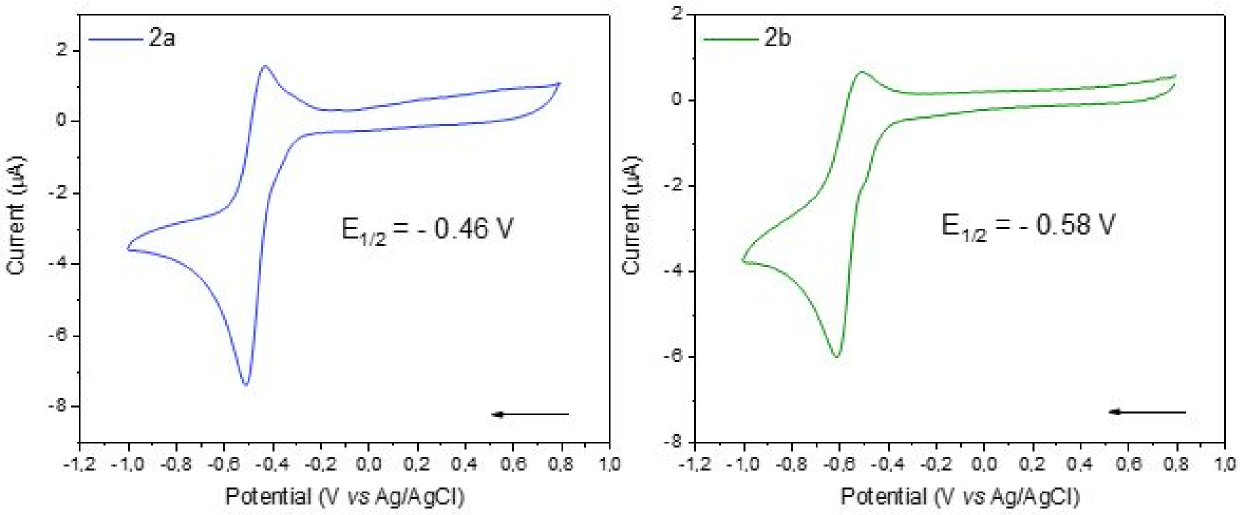

Figure S24. Cyclic voltammetry of 2a and 2b complexes in acetonitrile carried out at 100 mV s^{-1} using 0.1 mol L^{-1} of tetrabutylammonium perchlorate (TBAP) as electrolyte. Solutions of the complexes of concentration $1x10^{-3}$ mol L^{-1} were prepared for the measurements.

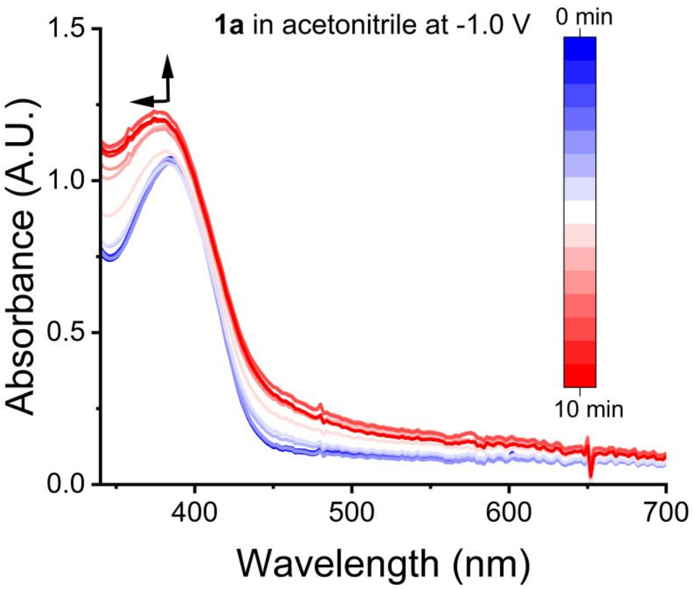

Figure S25. Spectroelectrochemical experiment of **1a** in acetonitrile. The applied potential was -1.0 V (vs Ag/AgCl, 3.5 molL^{-1}). Experiment was carried out in a solution containing 0.1 mol L^{-1} of tetrabutylammonium perchlorate (TBAP) as electrolyte.

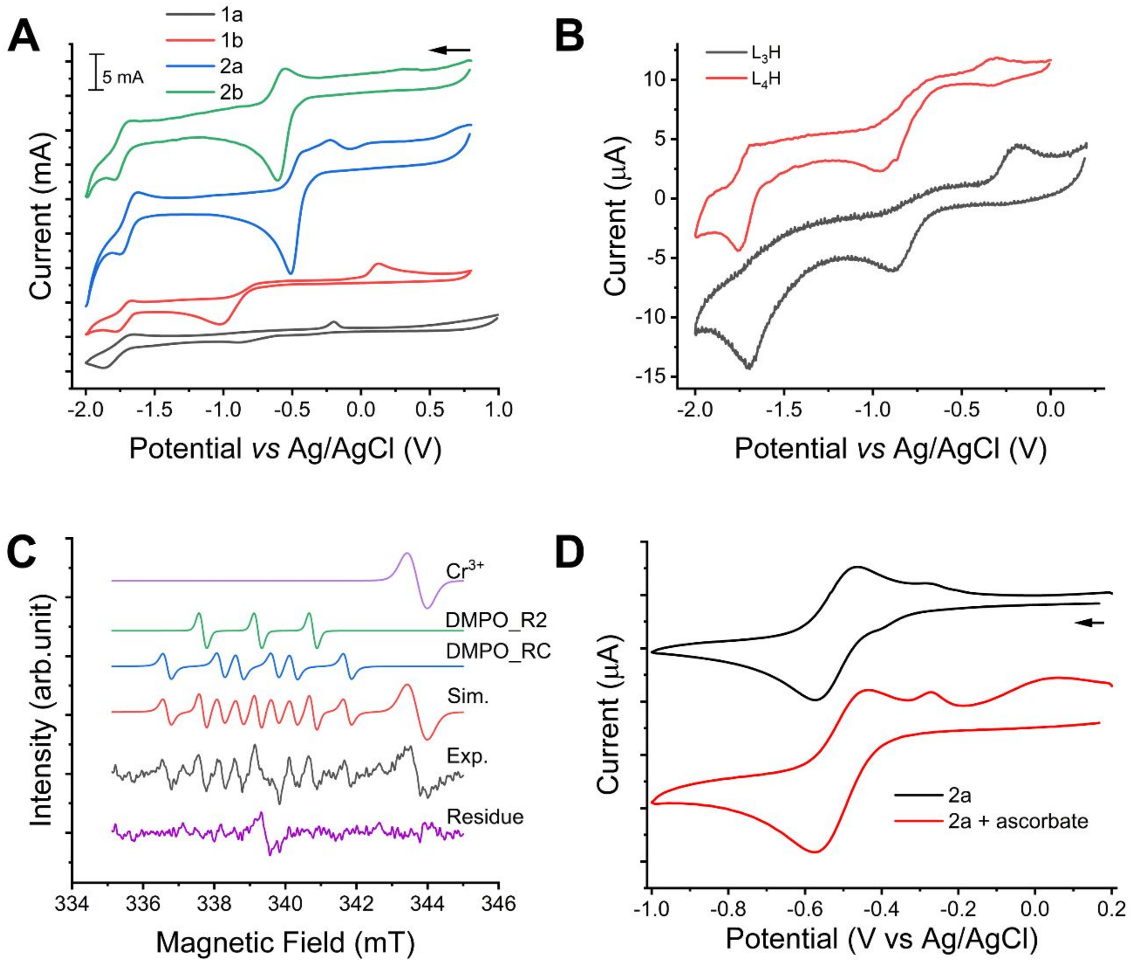

Figure S26. Cyclic voltammetry experiments in acetonitrile of (A) all complexes over the range of -2.0 V to +0.8 V; (B) ligands L3H (black line) and L4H (red line) over the range of -2.0 V to +0.2 V; (C) EPR spectra of the reaction of ligand L3H with ferricyanide in alkaline acetonitrile medium. Two different radicals were trapped with DMPO, the hydroxyl and a carbon centered one. (D) complex **2a** in the absence and presence of 1 equivalent of ascorbate (range -1.0 V to +0.2V). All experiments were carried out in a solution containing 0.1 mol L^{-1} of tetrabutylammonium perchlorate (TBAP) as electrolyte.

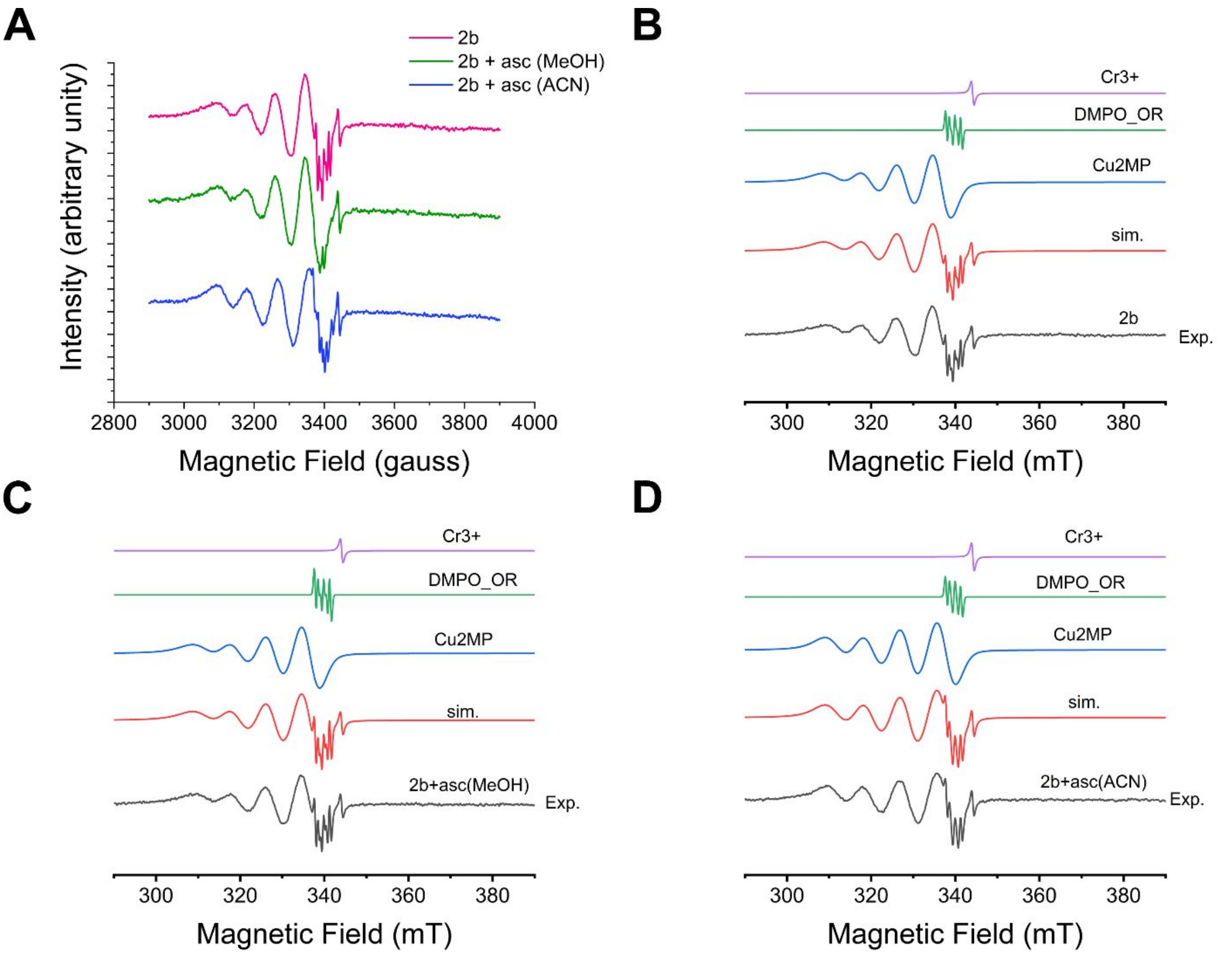

Figure S27. EPR spectroscopy of **2b** before and after the addition of sodium ascorbate in methanol and in acetonitrile(A) and simulation of the EPR spectra of **2b** (B), **2b** in the presence of ascorbate in methanol (C) and **2b** in the presence of ascorbate in acetonitrile. Spin quantification of **2b** reveal that Cu(II) is relative to 85% of the solution in all cases. Spin quantification of the radicals present in these experiments add to 1%. The error of spin quantification is 10%.

Table S1: EPR Parameters for 1b and 2b in methanol at T = 77 Kelvin before and after addition of azide and ascorbate.

Compound	1b	+Azide+Asc.	2b	+Azide+Asc.
g-values	2.0364<br>2.0563<br>2.2340	2.0396<br>2.0425<br>2.2207	2.0320<br>2.0697<br>2.2068	2.0330<br>2.0608<br>2.2065
Linewidth (mT)	0.791<br>0.725	0.865<br>0.453	3.320<br>0.701	3.213<br>0.527
ACu (MHz)	51.65<br>57.52<br>550.96	53.24<br>82.83<br>561.52	10.02<br>29.80<br>565.10	22.78<br>35.66<br>567.15
AN1 (MHz)	46.18<br>42.93<br>3.94	37.83<br>48.63<br>23.74	- - - - - -<br>- - - - - -<br>- - - - - -	- - - - - -<br>- - - - - -<br>- - - - - -
AN2 (MHz)	35.66<br>31.24<br>5.15	51.59<br>28.26<br>5.54	- - - - - -<br>- - - - - -<br>- - - - - -	- - - - - -<br>- - - - - -<br>- - - - - -
Q – values (MHz)	22.08<br>-8.73	28.88<br>-5.08	32.87<br>-7.76	26.40<br>-7.37

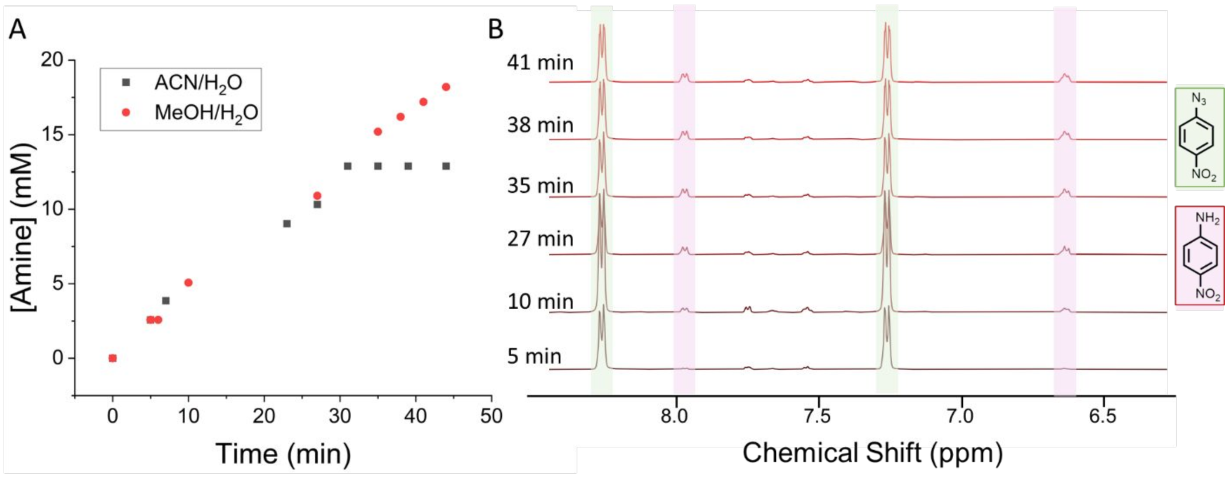

Figure S28. Concentration of amine over time in a reaction performed by complex 1b in acetonitrile/water and methanol/water mixture (A) and ^{1}H NMR spectra at different reaction times of the reaction of azide reduction in methanol/water by complex 1b (B).

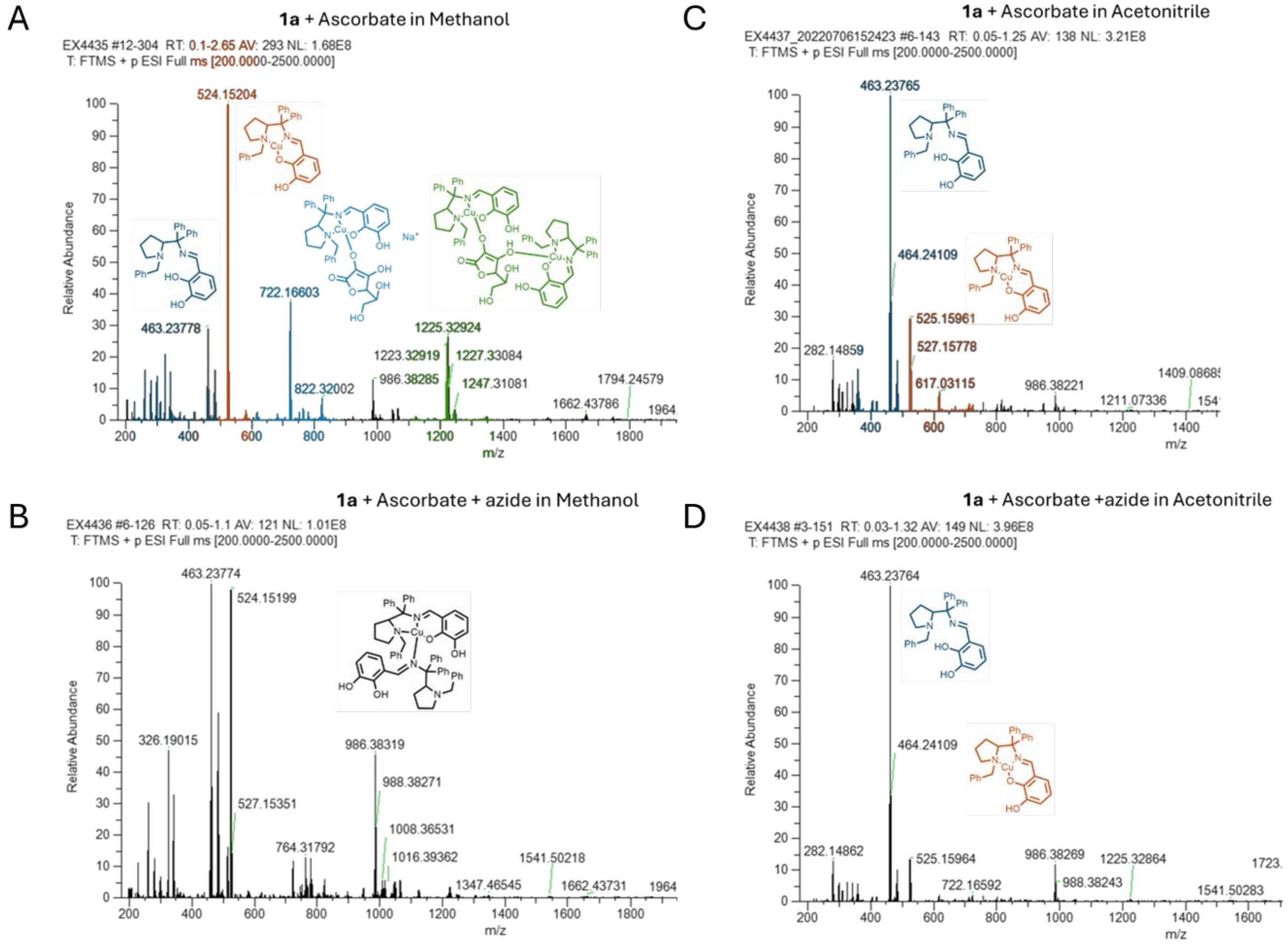

Figure S29. HRMS of **1a** at different reaction conditions. (A) **1a** was dissolved in methanol in the presence of ascorbate; (B) **1a** was dissolved in methanol in the presence of sodium ascorbate and

p-nitrophenylazide; (C) **1a** was dissolved in acetonitrile in the presence of ascorbate and (D) **1a** was dissolved in acetonitrile in the presence of ascorbate and p-nitrophenylazide.

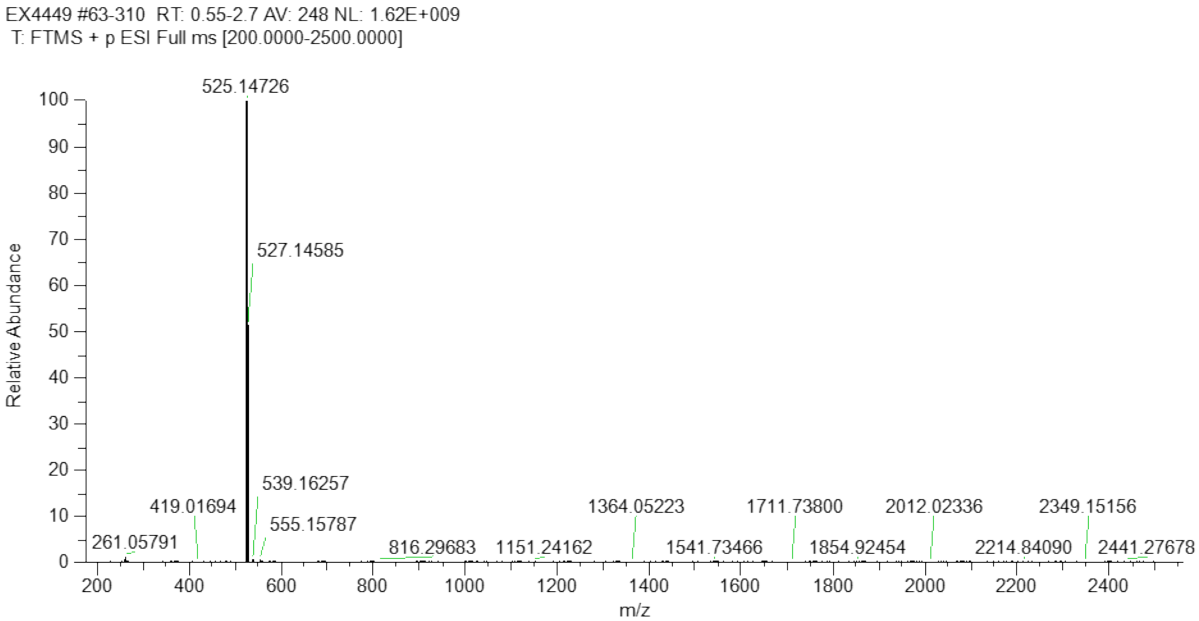

Figure S30. HRMS of **2a** in methanolic solution after the addition of ascorbate in the presence of water (A) revealing the intact complex.

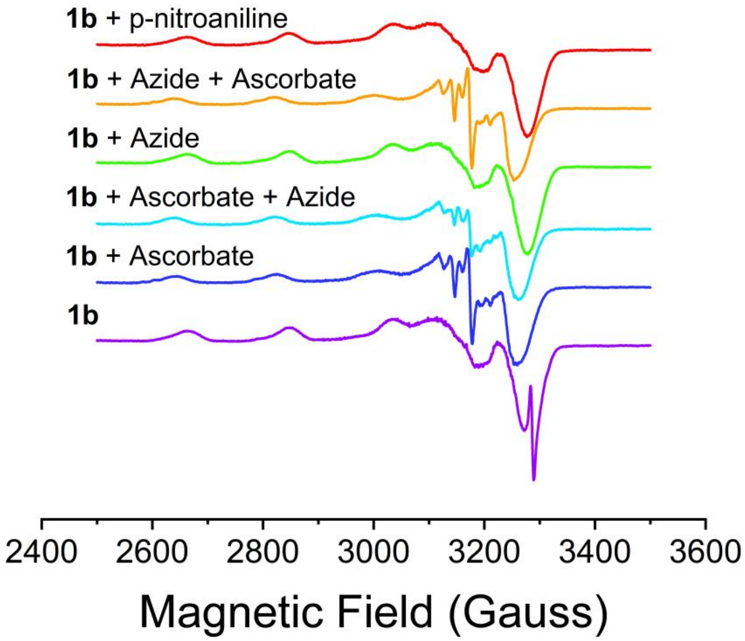

Figure S31. EPR spectroscopy of **1b** in methanol before and after the addition of ascorbate and azide at different order of addition. The spectrum of **1b** after addition of p-nitroaniline is also shown. These EPR spectra were recorded at 77K.

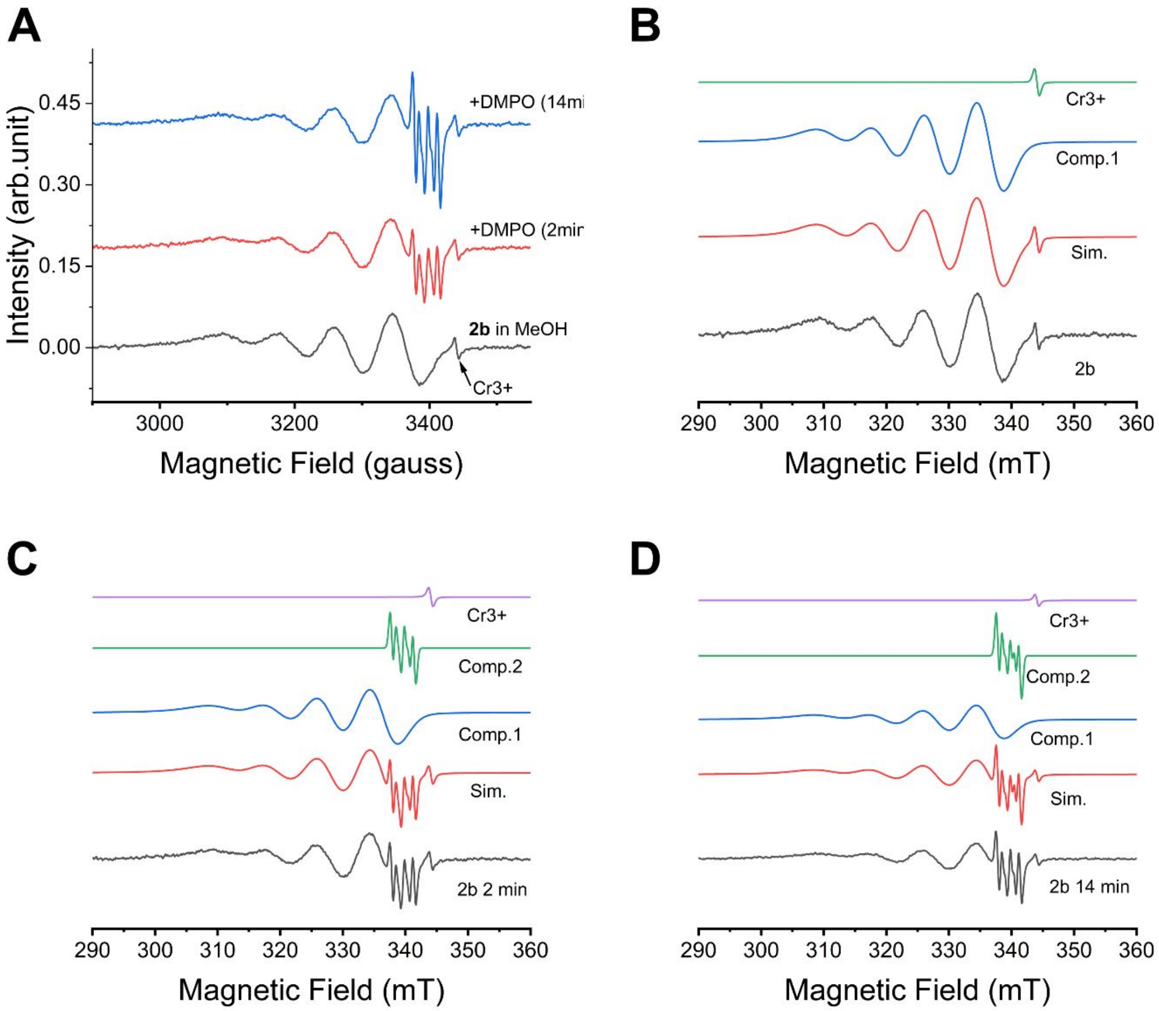

Figure S32. EPR spectroscopy of 2**b** in methanol before and after the addition of DMPO, evidencing that the copper center is intact in its structure and geometry after DMPO addition(A). Detailed simulation of the EPR spectra of **2b** before the addition of DMPO (B) and in the presence of DMPO at 2 minutes (C) and at 14 minutes of reaction (D). These EPR spectra were recorded at room temperature. Spin quantification at 0 min reveals 81% of Cu(II) before addition of DMPO. After the addition of DMPO, the Cu(II) remained the same and 2.5% of O-radical were detectable. At 14 minutes quantification indicates 83% of Cu(II) and 4.2% of O-radical.

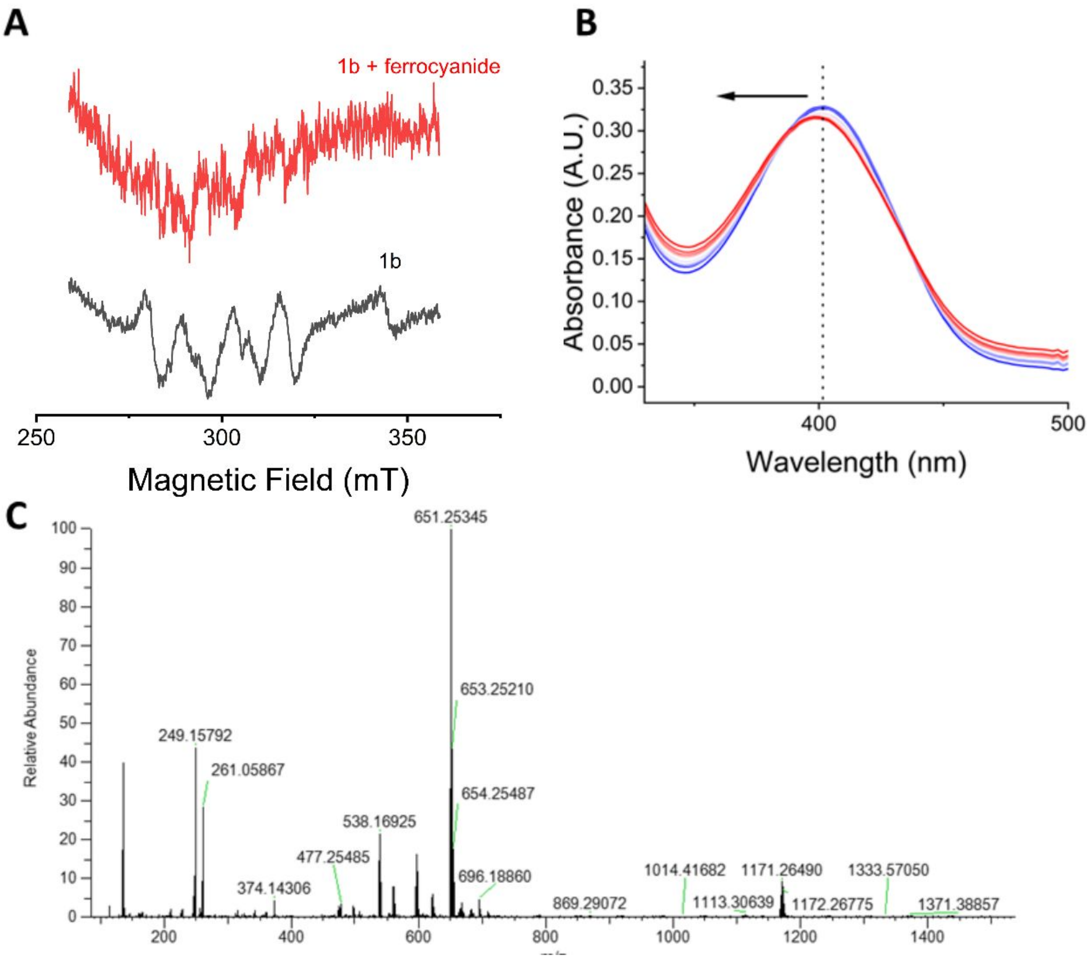

Figure S33. Interaction between **1b** and potassium ferrocyanide. (A) EPR spectroscopy of **1b** in methanolic solution before and after the addition of ferrocyanide; (B) UV-Vis spectroscopy of 1b in methanolic solution before (blue) and after the addition of ferrocyanide (red) and (C) HRMS of DMPO-trapped **1b** radical.

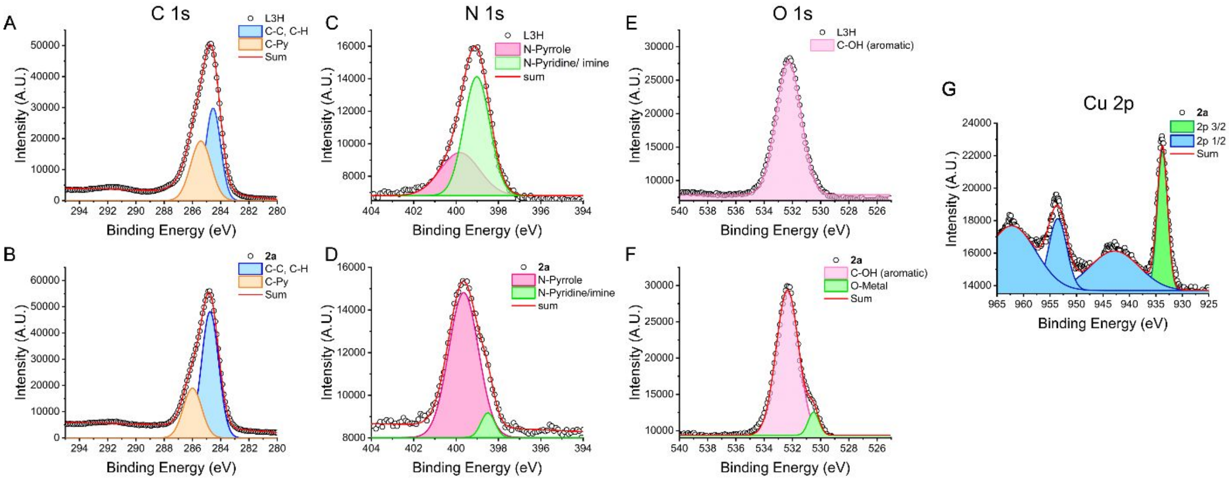

Figure S34. XPS spectra and their deconvolutions of ligand **L3H** and complex **2a**. a) C1s binding energies of **L3H**, b) C1s binding energies of **2a**, c) N1s binding energies of **L3H**, d) N1s binding energies of **2a**, e) O1s binding energies of **L3H**, f) O1s binding energies of **2a** and g) Cu2p binding energies of **2a**.

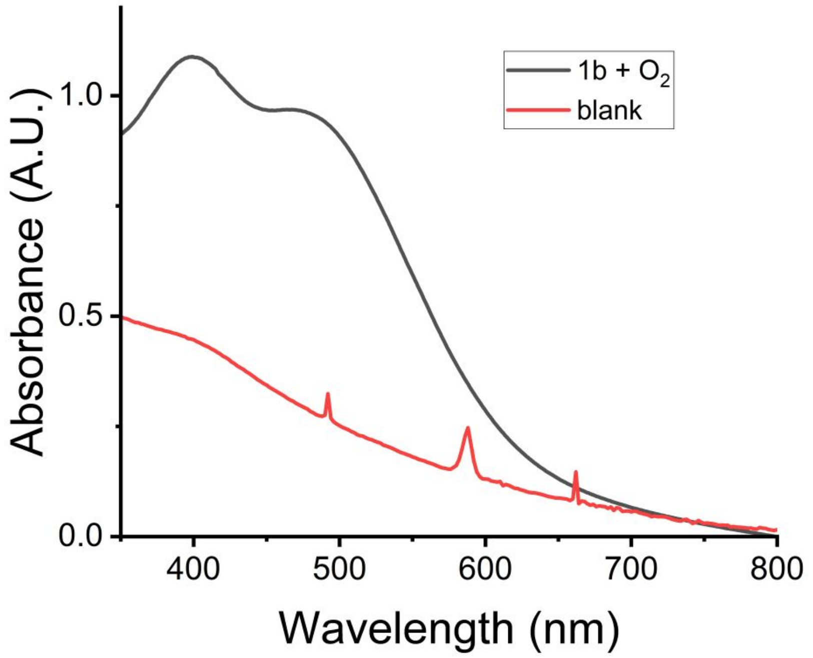

Figure S35. Colorimetric detection of hydrogen peroxide formation upon O_2 bubbling. A methanolic solution of complex 1b was bubbled with O_2 for 20 minutes and then 1 mL of this reaction was reacted with 0.2 mL of 10 mM ammonium sulfate $[Fe(NH_4)_2SO_4]$ and 0.1 ml of 2.5 M potassium thiocyanate (KSCN). The reaction was incubated for 5 minutes at room temperature before being stopped by addition of 0.2 mL of trichloroacetic acid. The purple reading ferrithiocyanate complex formation is observed by the presence of a band at 480 nm (ε= 3860 $molL^{-1}$ cm^{-1}). Blank experiments were performed with a copper complex that does not form Cu(II)-phenoxyl species in solution.

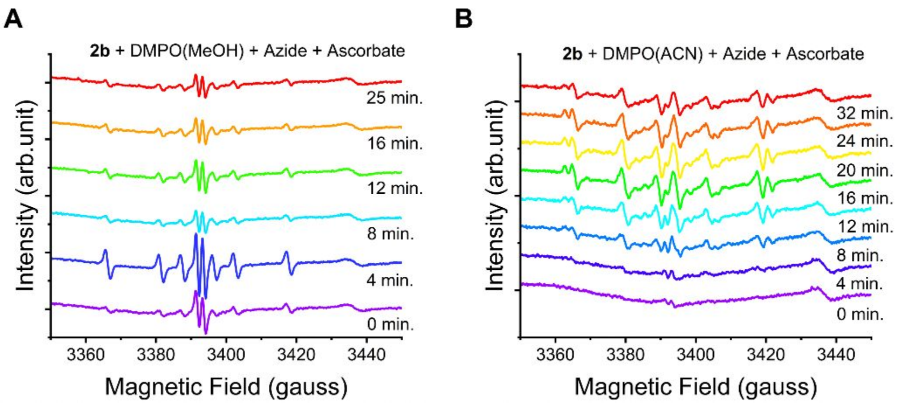

Figure S36. Time-resolved EPR spectra recorded at room temperature of the reaction between **2b**, azide and ascorbate in the presence of DMPO as a radical trapping agent. (A) reaction performed in methanol and (B) reaction performed in acetonitrile.

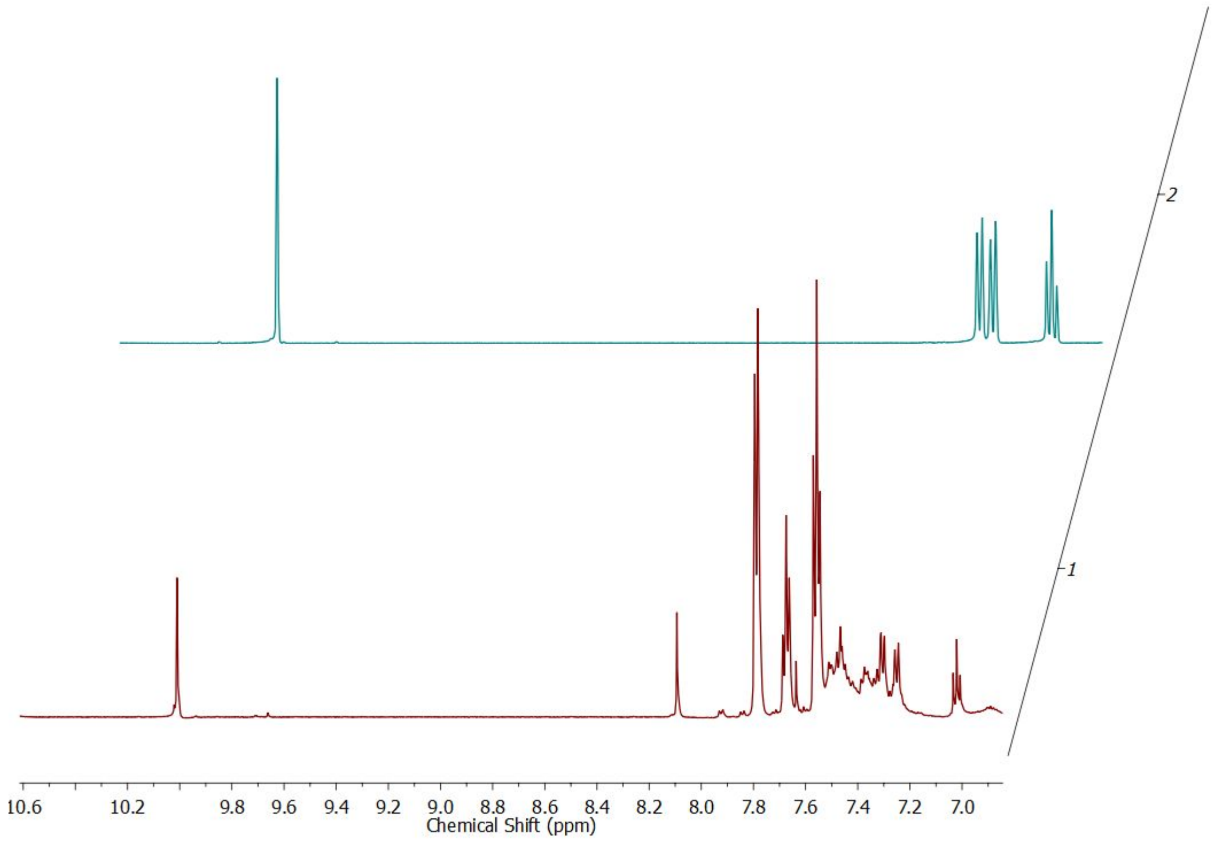

Figure S37. ^{1}H NMR spectra of O-vaniline (top) and of the reaction between **1b** and sodium ascorbate in acetonitrile/water medium (bottom), evidencing the formation of O-vaniline in the reaction.

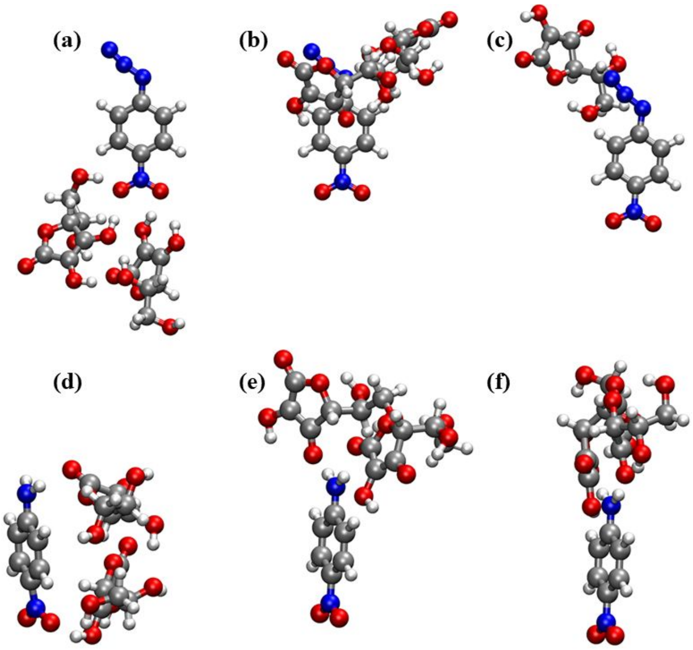

Figure S38 – Structures most stable for (a) Ascorbic Acid/Nitrophenyl-Az (b) Ascorbate/Nitrophenyl-Az (c) Ascorbyl/Nitrophenyl-Az, (d) Ascorbic Acid/Nitrophenyl-Am (e) Ascorbate/Nitrophenyl-Am and (f) Ascorbyl/Nitrophenyl-Am. Color-code: O (red), N (blue), C (silver) and H (white).

Table S2 – Gibbs Free energies at 298.15 K for equation **Figure 8F** as a function from solvent.

**Cu-System (Solvent)**	**ΔG° / kJ mol^{-1}**	
	**Chain**	**Bicyclic**
**1c (CH_3OH)**	-65.3	-72.9
**1c (CH_3CN)**	-12.1	-14.2
**2a (CH_3OH)**	-44.6	-48.2
**2a**	-8.5	-8.9

**(CH_3CN)**		
**2c (CH_3OH)**	-28.9	-31.1
**2c (CH_3CN)**	-10.3	-11.2

Table S3. Crystal data and structure refinement for **1c**, **2c** and **2a**.

	**1a**	**2c**	**2a**
**CCDC code**	2359023	2359024	2359022
**Empirical formula**	$C_{33}H_{35}CuN_3O_6$	$C_{31}H_{30}CuN_4O_5$	$C_{32}H_{36}ClCuN_3O_8$
**Formula weight**	633.208	602.753	689.63
**Temperature/K**	293	293	293
**Crystal system**	monoclinic	orthorhombic	triclinic
**Space group**	$P2_1$	$P2_12_12_1$	P1
**a/Å**	9.5579(7)	9.1808(7)	8.6508(2)
**b/Å**	14.3928(11)	11.8976(11)	9.47520(10)
**c/Å**	10.692(2)	25.375(2)	10.6778(3)
**α/°**	90	90	98.727(2)
**β/°**	96.525(13)	90	113.352(2)
**γ/°**	90	90	90.4310(10)
**Volume/Å^3**	1461.3(4)	2771.7(4)	792.03(3)
**Z**	2	4	1
**ρ_{calc}g/cm^3**	1.439	1.444	1.446
**μ/mm^{-1}**	0.799	0.837	2.217
**F(000)**	663.1	1255.4	359
**Crystal size/mm^3**	0.332 × 0.31 × 0.183	0.254 × 0.2 × 0.191	0.138 × 0.095 × 0.039
**Radiation**	Mo Kα (λ = 0.71073)	Mo Kα (λ = 0.71073)	Cu Kα (λ = 1.54184)
**2Θ range for data collection/°**	5.42 to 51.5	5.48 to 54	9.152 to 140.15
**Index ranges**	$-15 \le h \le 15$, $-23 \le k \le 22$, $-16 \le l \le 17$	$-14 \le h \le 13$, $-14 \le k \le 17$, $-29 \le l \le 39$	$-10 \le h \le 10$, $-11 \le k \le 11$, $-13 \le l \le 13$
**Reflections collected**	44748	22023	16370
**Independent reflections**	5571 [R_{int} = 0.1102, R_{sigma} = 0.0775]	5930 [R_{int} = 0.0350, R_{sigma} = 0.0551]	4517 [R_{int} = 0.0637, R_{sigma} = 0.0704]
**Data/restraints/parameters**	5571/73/335	5930/82/399	4517/6/413

**Goodness-of-fit on F^2**	1.046	1.063	1.054
**Final R indexes [I>=2σ (I)]**	R_1 = 0.0609, wR_2 = 0.1500	R_1 = 0.0315, wR_2 = 0.0704	R_1 = 0.0373, wR_2 = 0.0849
**Final R indexes [all data]**	R_1 = 0.0711, wR_2 = 0.1670	R_1 = 0.0409, wR_2 = 0.0764	R_1 = 0.0384, wR_2 = 0.0859
**Largest diff. peak/hole / e Å^{-3}**	0.55/-0.56	0.25/-0.21	0.61/-0.54
**Flack parameter**	-0.026(8)	-0.016(6)	0.018(16)

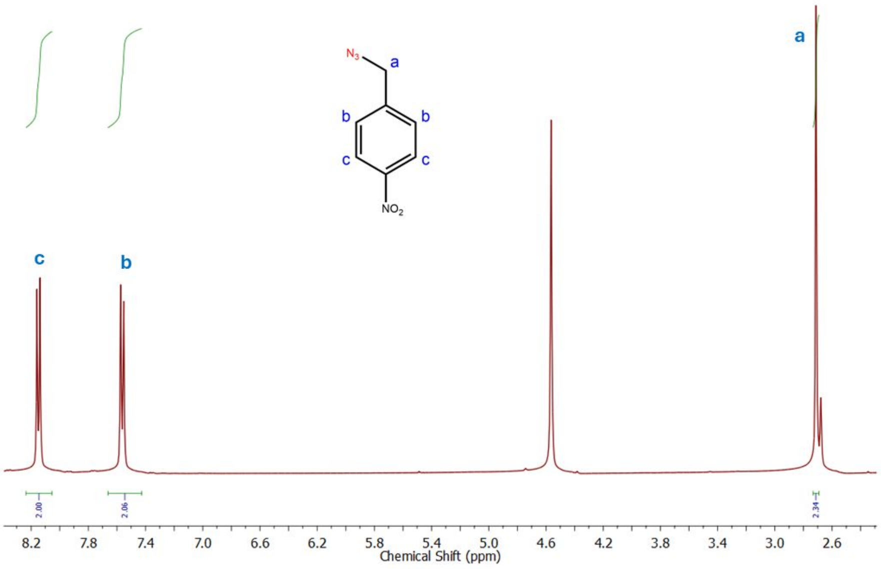

Figure S39. ^{1}H NMR of 1-(azidomethyl)-4-nitrobenzene.

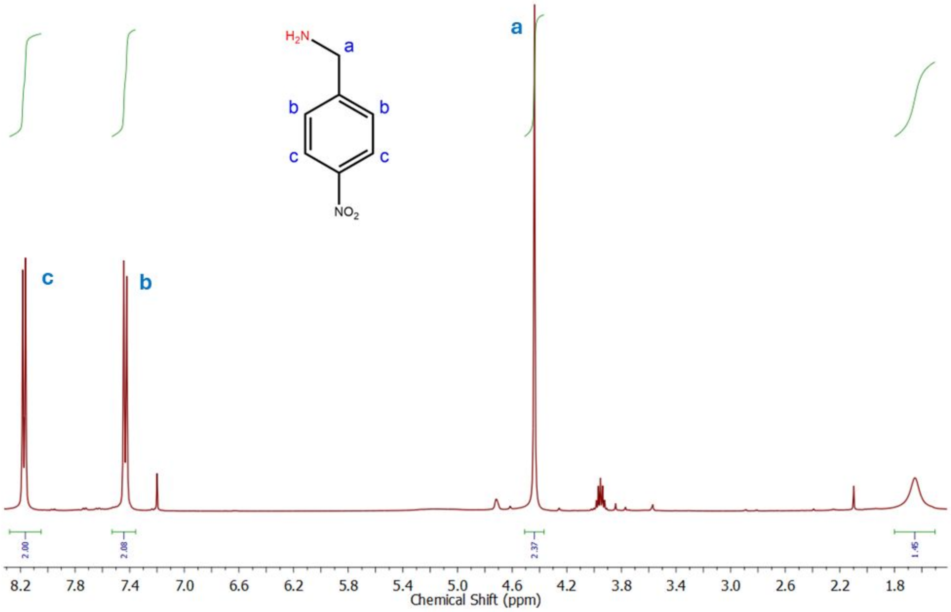

Figure S40. ^{1}H NMR of the reaction product of the reduction of 1-(azidomethyl)-4-nitrobenzene evidencing the formation of 1-(aminomethyl)-4-nitrobenzene.

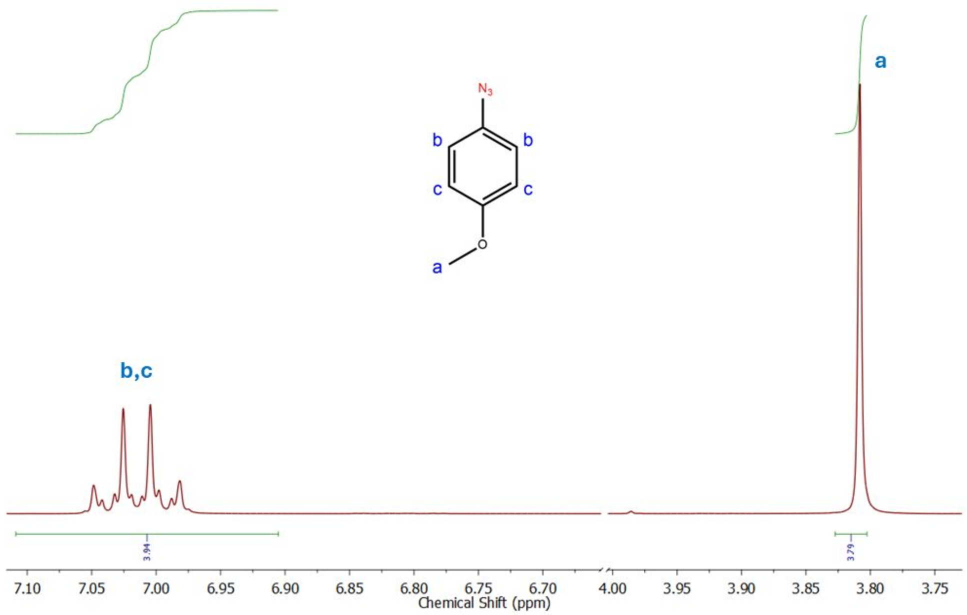

Figure S41. ^{1}H NMR of 1-azido-4-methoxybenzene.

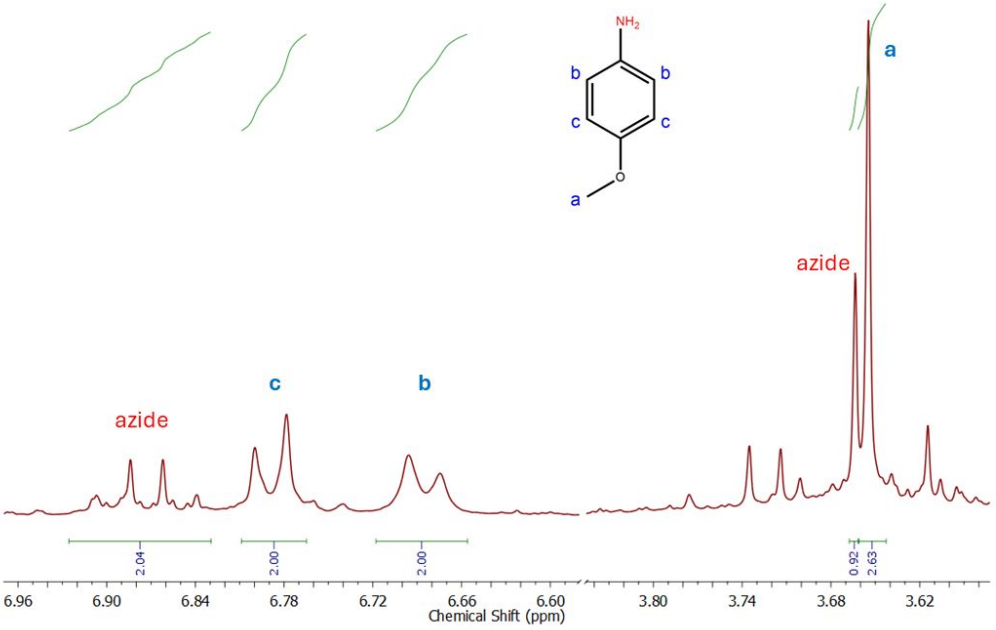

Figure S42. ^{1}H NMR of the reaction product of the reduction of 1-azido-4-methoxybenzene evidencing the formation of 1-amino-4-methoxybenzene.

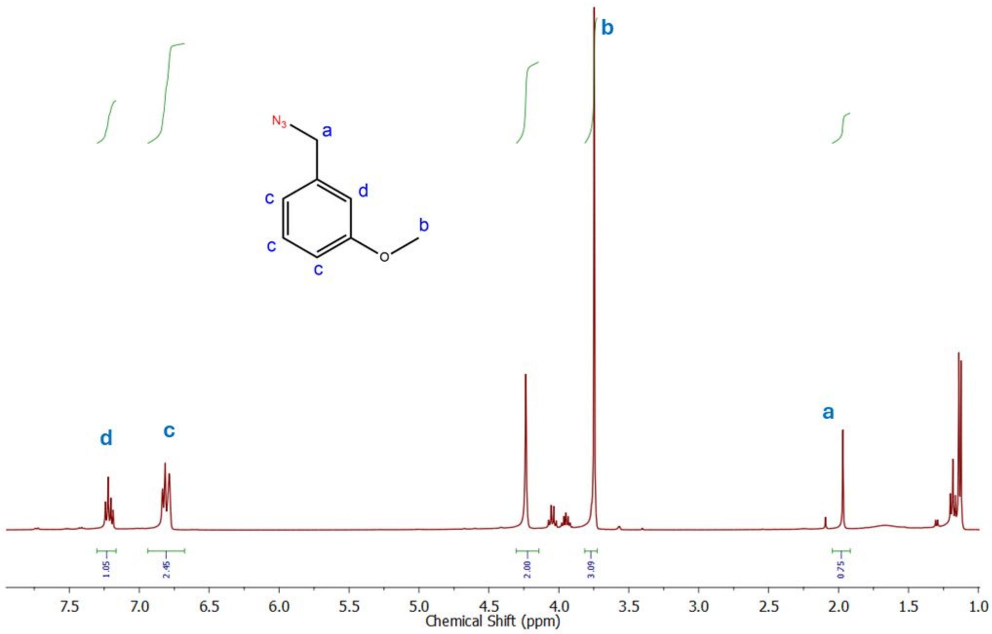

Figure S43. ^{1}H NMR of 1-(azidomethyl)-3-methoxybenzene.

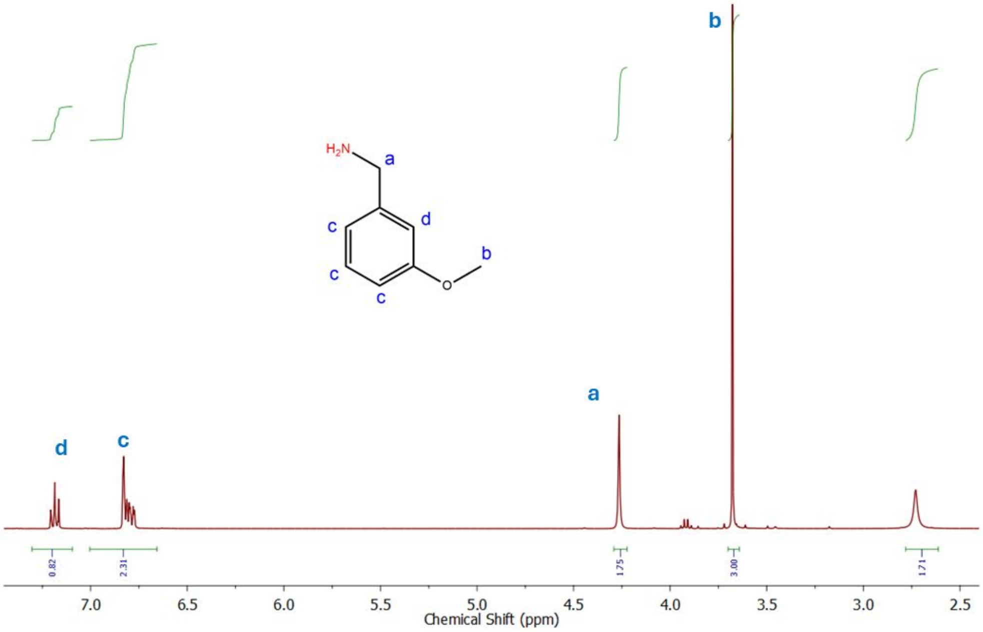

Figure S44. ^{1}H NMR of the reaction product of the reduction of 1-(azidomethyl)-3-methoxybenzene evidencing the formation of 1-(aminomethyl)-3-methoxybenzene.

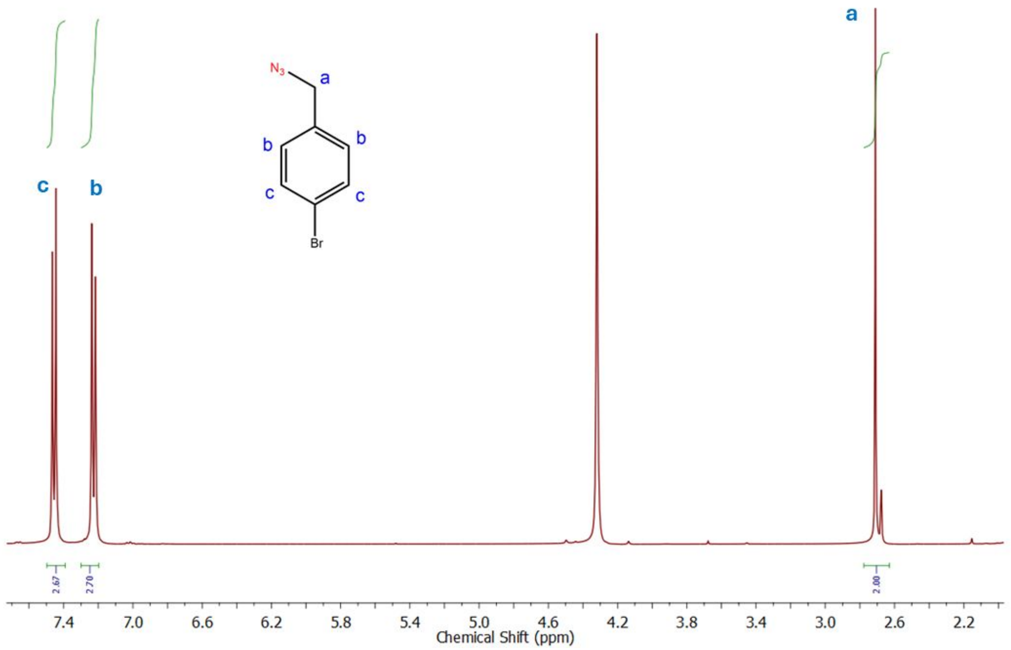

Figure S45. ^{1}H NMR of 1-(azidomethyl)-4-bromobenzene.

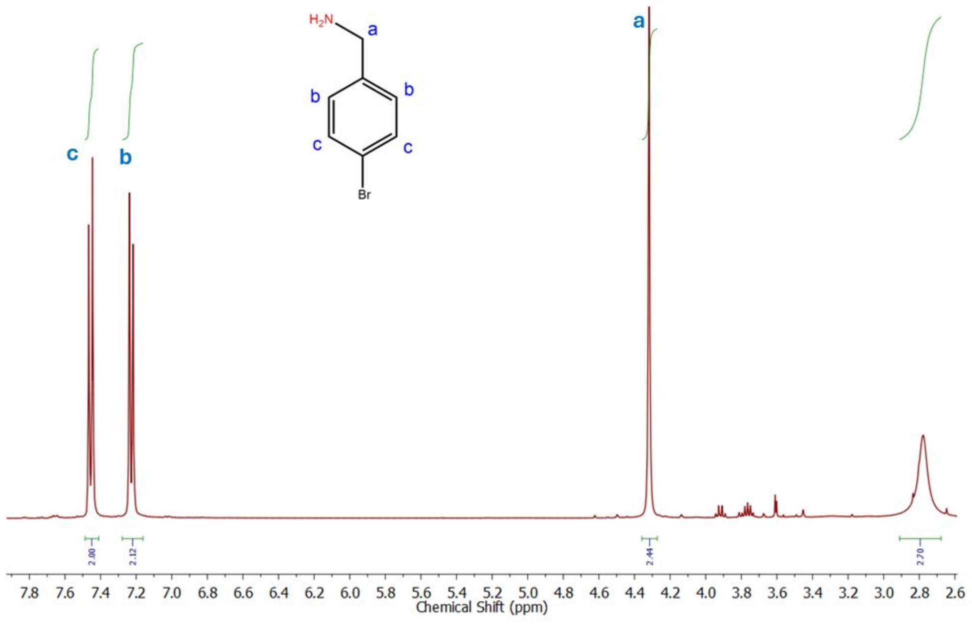

Figure S46. ^{1}H NMR of the reaction product of the reduction of 1-(azidomethyl)-4-bromobenzene, evidencing the formation of 1-(aminomethyl)-4-bromobenzene.

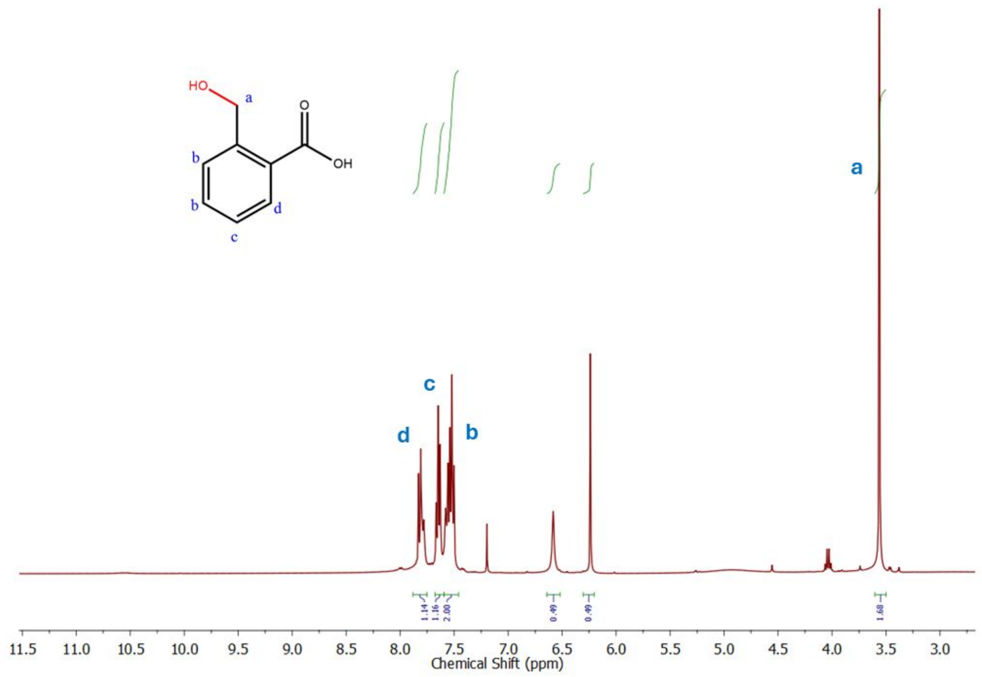

Figure S47. ^{1}H NMR of the reaction product of the reduction of 2-formylbenzoic acid, evidencing the formation of 2-(hydroxymethyl)benzoic acid.

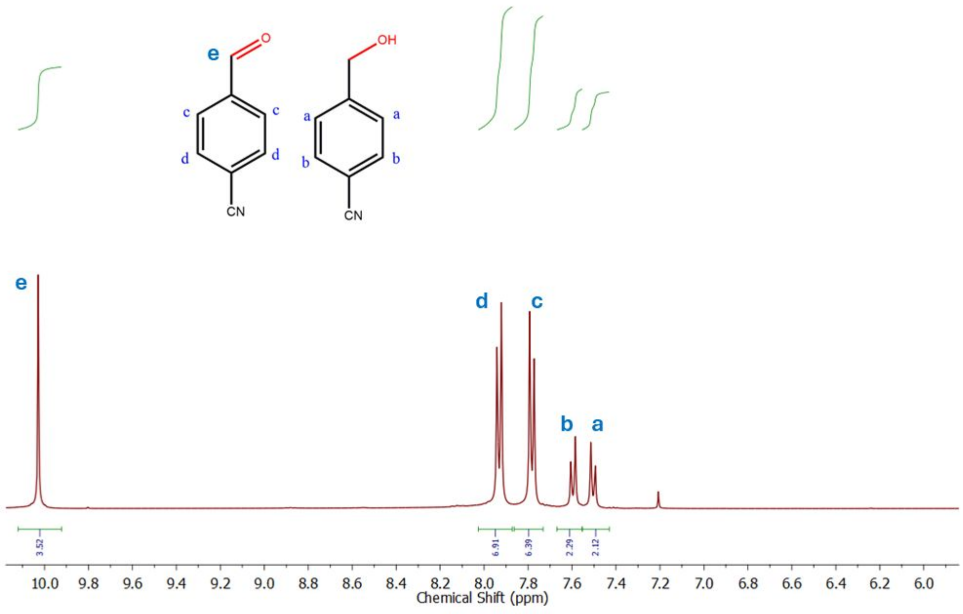

Figure S48. ^{1}H NMR of the reaction product of the reduction of 4-formylbenzonitrile, evidencing the formation of 4-(hydroxymethyl)benzonitrile.

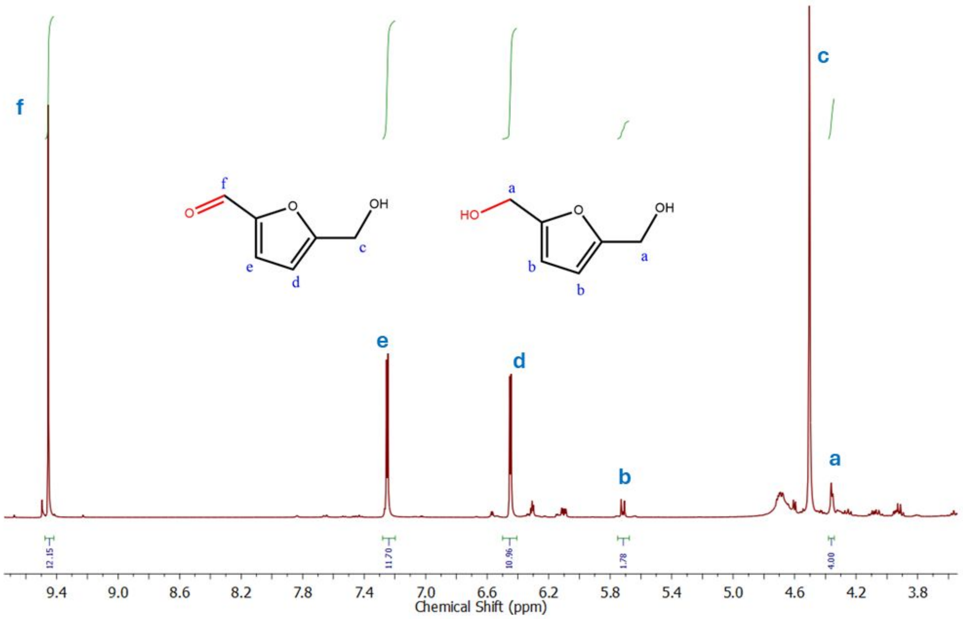

Figure S49. ^{1}H NMR of the reaction product of the reduction of 5-(hydroxymethyl)furan-2-carbaldehyde, evidencing the formation of furan-2,5-diyldimethanol.

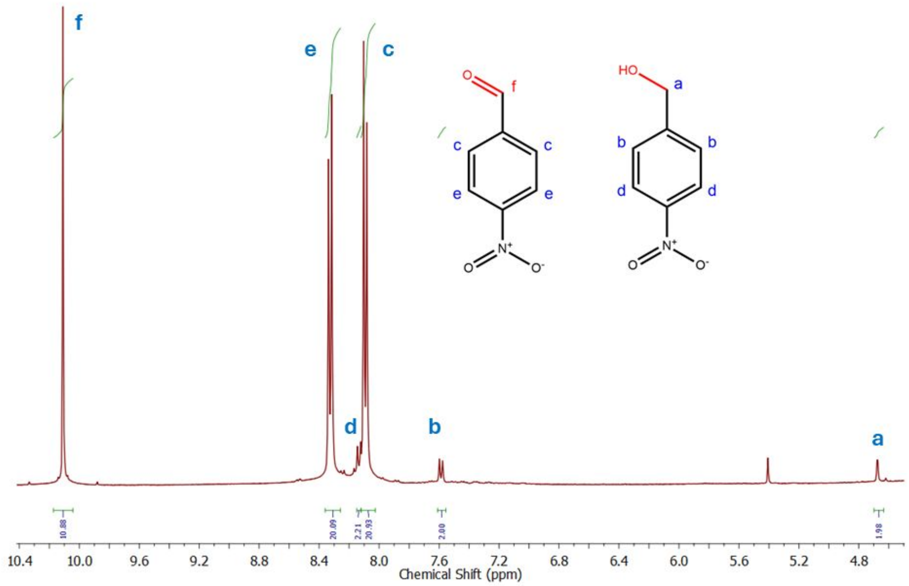

Figure S50. ^{1}H NMR of the reaction product of the reduction of 4-nitrobenzaldehyde, evidencing the formation of (4-nitrophenyl)methanol.

1. C. B. Castro, R. G. Silveira, F. M. Colombari, A. F. d. Moura, O. R. Nascimento and C. G. C. M. Netto, Solvent Effect on the Regulation of Urea Hydrolysis Reactions by Copper Complexes *Chemistry*, 2020, **2**, 525-544.
2. M. P. Ferreira, C. B. Castro, J. Honorato, S. He, W. G. G. Júnior, C. Esmieu, E. E. Castellano, A. F. d. Moura, D. R. Truzzi, O. R. Nascimento, A. Simonneau and C. G. C. M. Netto, Biomimetic catalysis of nitrite reductase enzyme using copper complexes in chemical and electrochemical reduction of nitrite, *Dalton Trans.*, 2023, **52**, 11254-11264.
